# Protective effects and mechanism of resveratrol in animal models of pulmonary fibrosis: a preclinical systematic review and meta-analysis

**DOI:** 10.3389/fphar.2025.1666698

**Published:** 2025-09-29

**Authors:** Yajie Yin, Nan Jia, Hui Luo, Xinglin Feng, Xin He, Ju Huang, Fei Wang

**Affiliations:** ^1^ School of Clinical Medicine, Chengdu University of Traditional Chinese Medicine, Affiliated Hospital of Chengdu University of Traditional Chinese Medicine, Chengdu, Sichuan, China; ^2^ Chengdu Shuangliu Area Gongxing Community Health Service Centers, Chengdu, Sichuan, China; ^3^ Chengdu Integrated TCM&Western Medicine Hospital, Chengdu, Sichuan, China; ^4^ Department of Respiratory Medicine, Hospital of Chengdu University of Traditional Chinese Medicine, Chengdu, Sichuan, China; ^5^ Department of Geriatrics, Hospital of Chengdu University of Traditional Chinese Medicine, Chengdu, Sichuan, China

**Keywords:** pulmonary fibrosis, resveratrol, animal models, mechanism, meta-analysis

## Abstract

**Background:**

Pulmonary fibrosis (PF) is a chronic lung disease characterized by ongoing interstitial scarring. Current treatments can only slow the progression of the disease. Resveratrol (RES), a natural polyphenolic compound, has become a potential therapy for PF because of its multiple biological effects, including anti-fibrotic, anti-inflammatory, and antioxidant properties.

**Objectives:**

To clarify RES’s efficacy, safety, and mechanism of action in treating PF through a preclinical systematic review.

**Methods:**

A computerized search of eight databases (up to 6 March 2025) was conducted to identify *in vivo* animal experiments on RES treatment for PF. The SYRCLE tool was used to assess the risk of bias, and meta-analysis was performed using RevMan 5.4 and Stata 17.0. The outcome measures included two main aspects: core pathological processes and molecular mechanisms. Heterogeneity was assessed with the *I* ^*2*^ test, and publication bias was evaluated using funnel plots and Egger’s test.

**Results:**

A total of 25 studies were included, involving 628 animals in the experimental groups and 357 animals in the control groups. Meta-analysis of selected outcome measures showed: 1. Improved fibrosis: significant reduction in pulmonary fibrosis score (SMD = −2.30, 95% CI [−2.80, −1.79], *p* < 0.00001, *I* ^*2*^ = 76%) and decreased Hyp content (SMD = −2.16, 95% CI [−2.69, −1.63], *p* < 0.00001, *I* ^*2*^ = 85%); 2. Inhibited inflammation: reduced TNF-α content (SMD = −1.58, 95% CI [−2.18, −0.99], *p* < 0.00001, *I* ^*2*^ = 70%) and decreased IL-6 content (SMD = −2.16, 95% CI [−2.74, −1.59], *p* = 0.007, *I* ^*2*^ = 57%); 3. Restored oxidative balance: decreased MDA content (SMD = −2.22, 95% CI [−3.09, −1.35], *p* = 0.06, *I* ^*2*^ = 55%) and increased SOD content (SMD = 1.67, 95% CI [1.05, 2.30], *p* < 0.0001, *I* ^*2*^ = 76%).

**Conclusion:**

RES significantly enhances the pathological process in PF animal models by regulating the TGF-β/Smad and NF-κB pathways. Future efforts should focus on optimizing preclinical study designs to decrease heterogeneity and improve clinical translation.

**Systematic Review Registration:**

https://www.crd.york.ac.uk/PROSPERO/view/, Identifier CRD420251009847.

## 1 Introduction

Pulmonary fibrosis (PF) is a chronic lung disease characterized by progressive scarring of the lung interstitium (Koudstaal et al., 2023). Its pathological features mainly include abnormal activation of fibroblasts and excessive extracellular matrix (ECM) deposition, which lead to decreased lung compliance and impaired diffusion (Raghu et al., 2018), ultimately causing respiratory failure and even death. PF shows a significant age-related correlation and is more common in middle-aged and older men (Mortimer et al., 2020); the incidence rate in men is 1.5–2 times higher than in women (Hutchinson et al., 2015). Clinical data indicate that the median survival time for untreated PF patients is only 3–5 years, with a 5-year mortality rate exceeding 80% (Hutchinson et al., 2015; Raghu et al., 2016; Jo et al., 2017; Wijsenbeek and Cottin, 2020; Raghu et al., 2022; Gupta et al., 2024). Its prognosis is even worse than that of many malignant tumors.

The pathogenesis of PF involves the synergistic action of multiple factors, primarily including alveolar epithelial cell damage, abnormal repair responses, and excessive activation of fibroblasts (Cannito et al., 2010; Lebel et al., 2022; Jannini-Sá et al., 2024). Research has shown that repeated damage to alveolar epithelial cells is the initiating step in PF. Following damage, epithelial cells display abnormal activation of the TGF-β/Smad and Wnt/β-catenin signaling pathways (Inui et al., 2021; Wu J. et al., 2022), leading to dysregulated epithelial-mesenchymal transition (EMT) and promoting the transformation of fibroblasts into myofibroblasts (Selman and Pardo, 2020). Single-cell sequencing technology further revealed that lung tissue from PF patients contains a unique subpopulation of pro-fibrotic epithelial cells that highly express pro-fibrotic factors and directly drive pathological ECM deposition (Adams et al., 2020). Additionally, oxidative stress imbalance is critical in PF progression (Otoupalova et al., 2020). Mitochondrial dysfunction-induced excessive accumulation of reactive oxygen species (ROS) damages alveolar epithelial cells, causes abnormal repair, and activates the TGF-β1 signaling pathway to accelerate fibrosis (Allen et al., 2017; Mora et al., 2017). The chronic inflammatory microenvironment and Th2-type immune response synergistically promote fibroblast proliferation, with activated macrophages forming a pro-fibrotic network by secreting inflammatory factors such as IL-1β, TNF-α, and IL-6 (Bringardner et al., 2008; Henderson et al., 2020).

Currently, PF cannot be reversed or completely cured (Karampitsakos et al., 2023). Although lung transplantation is a primary treatment method, it is limited by donor shortages and transplant rejection reactions, so only a small number of patients benefit from it, and the average survival period after surgery is only 4 years (Glass et al., 2022). Although the frontline drugs pirfenidone and nintedanib can slow disease progression (Mei et al., 2021; Podolanczuk et al., 2023), there are serious side effects (Galli et al., 2017; Liu et al., 2017). Therefore, it is crucial to find new treatment methods to prevent and treat PF.

**FIGURE 1:**
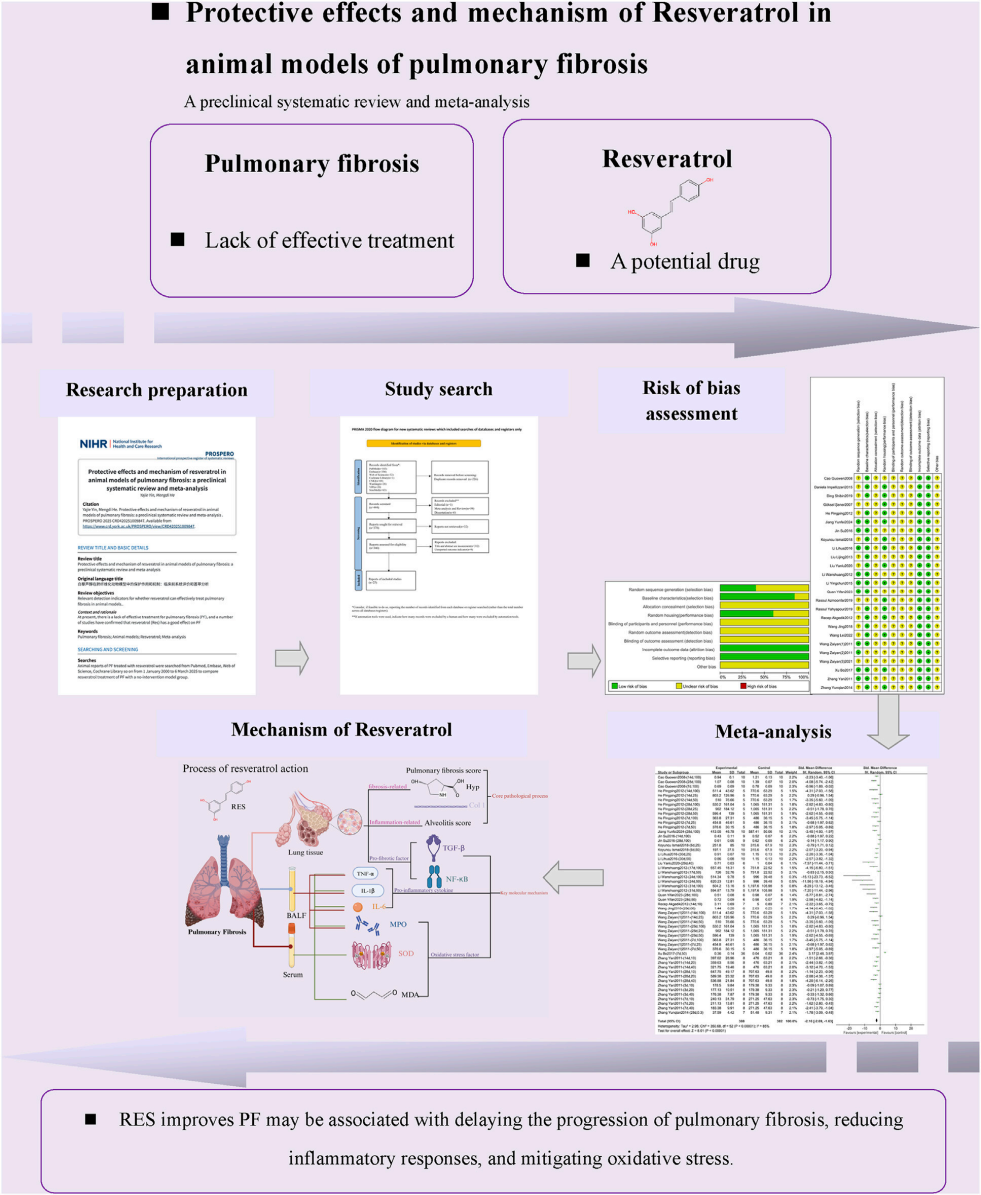
Research roadmap.

In recent years, substantial progress has been made in research on the prevention and treatment of PF using traditional Chinese medicine (Huang et al., 2022). Resveratrol (RES), whose chemical name is 3,5,4′-trihydroxy-trans-dibenzyl, is a non-flavonoid polyphenol found widely in plants such as grapes, giant knotweed, and peanuts (Tian and Liu, 2020). It has multiple biological effects, including anti-fibrotic, anti-inflammatory, and antioxidant properties (Koushki et al., 2020; Meng et al., 2021; Bao et al., 2022). Experimental studies indicate that RES can inhibit the progression of pulmonary fibrosis through multiple pathways: it targets and inhibits the core pro-fibrotic factor TGF-β1, blocking its mediation of abnormal α-SMA expression and ECM deposition (Willis and Borok, 2007); additionally, it directly inhibits fibroblast proliferation and their transformation into myofibroblasts (Chuliá-Peris et al., 2022). Notably, RES can also regulate mitochondrial function to reduce oxidative stress damage, thereby intervening in the fibrosis process at its source (Ramli et al., 2023). Although animal experiments have systematically validated RES’s efficacy, gaps remain in clinical translation, and reports of specific efficacy indicators across studies show heterogeneity. Preclinical research is fundamental to translational medicine, allowing for systematic assessment of drug mechanisms and helping shape clinical trial design (Zordoky et al., 2015). Specifically, animal models can mimic the disease’s progression in pulmonary fibrosis, offering essential evidence for target validation (Dludla et al., 2020). Therefore, systematically evaluating RES’s protective effects in PF animal models and its mechanisms will provide reliable evidence-based support for clinical application. A research roadmap is shown in Figure 1.

## 2 Materials and methods

This study was conducted per the Preferred Reporting Items for Systematic Reviews and Meta-Analyses (PRISMA) guidelines (Page et al., 2021). The protocol was pre-registered on the PROSPERO platform on 12 March 2025 (CRD 420251009847). This study strictly followed the Preferred Reporting Items for Systematic Reviews and Meta-Analyses (PRISMA) guidelines. The protocol was pre-registered on the PROSPERO platform on 12 March 2025. The pre-registered protocol specified Hyp content as the primary outcome measure, with secondary outcomes including TGF-β content, MPO content, TNF-α content, and MDA content. The study added additional outcome measures such as pulmonary fibrosis score, Col 1 content, and alveolitis score, further clarifying the core pathological processes and molecular mechanisms underlying RES therapy for PF (CRD 420251009847).

### 2.1 Inclusion and exclusion criteria

This study’s inclusion and exclusion criteria follow the population, intervention, comparison, outcome, and study design (PICOS) framework (Table 1).

**TABLE 1:** Inclusion and exclusion criteria.

	Inclusion criteria	Exclusion criteria
Population (P)	There are no restrictions on the species, weight, age or sex of the animals selected. Any animal model of pulmonary fibrosis (PF) will be included.	Exclude non-PF animal models and other disease-related PF animal experiments.
Intervention (I)	The experimental and control groups of the PF animal model were established using bleomycin, lipopolysaccharide, environmental particulate matter, radiation or silica. The route of administration, dosage, timing, source of the drug, and the purity of the drug are not restricted.	No use of bleomycin, lipopolysaccharide, environmental particulate matter, radiation or silica. Treatment with resveratrol analogues or in combination with other drugs.
Comparison (C)	The experimental group received resveratrol treatment. The control group was administered saline, deionised water or no treatment measures.	No control group design.
Outcome (O)	1. Core pathological processes: 1) Fibrosis-related: pulmonary fibrosis score, Hydroxyproline (Hyp) content, Collagen 1 (Col 1) content; 2) Inflammation-related: alveolitis score. 2. Key molecular mechanisms: 1) Pro-fibrotic factor (PKF): Transforming growth factor-β(TGF-β) content, Nuclear factor kappa-B(NF-κB) content; 2) Pro-inflammatory cytokine: Tumor necrosis factor-α(TNF-α) content, Interleukin-1β(IL-1β) content, Interleukin-6(IL-6) content; 3) Oxidative stress factor: Malondialdehyde (MDA) content, Myeloperoxidase (MPO) content, Superoxide Dismutase (SOD) content.	Did not achieve the expected outcome measures. Outcome measures are non-quantitative data.
Study design (S)	Randomized controlled animal experiments (*in vivo* studies) are required	The study type is a non-randomized controlled experiment, such as case reports, clinical trial studies, editorials, reviews, meta-analyses, and incomplete texts. The study type is a randomized controlled experiment (*in vitro* study). Literature for which the full text is unavailable or has been duplicated.

### 2.2 Information source and search strategy

Two researchers conducted computer searches of eight databases from their inception to 6 March 2025: PubMed, Embase, Web of Science, Cochrane Library, China National Knowledge Infrastructure (CNKI), Wanfang Data (Wanfang), VIP Database (VIP), and SinoMed. The main search terms included Resveratrol, 3,5,4′-Trihydroxystilbene, trans Resveratrol; Pulmonary Fibrosis, Fibrosing Alveolitis, Idiopathic Pulmonary Fibrosis, Acute Lung Injury, Respiratory Distress Syndrome; Animals, Models, Animal, Animals, Laboratory, Animal Experimentation. A combination of subject terms and free-text keywords was used for systematic retrieval, with different search strategies applied based on each database’s characteristics. Additionally, the references of included studies were traced to supplement the retrieval of relevant literature, with the search limited to Chinese and English. A detailed search strategy for each database is provided in Supplementary Table S1.

### 2.3 Study selection

Two researchers independently conducted literature screening and data extraction. First, the retrieved literature titles were imported into EndNote 20 software for initial screening and deduplication. Further screening was conducted based on inclusion and exclusion criteria after reading the titles and abstracts of the literature. Finally, the RCTs included in the quantitative analysis were determined by reading the full texts. If there were differences in opinion between the two researchers, a third researcher was consulted to resolve the issue.

### 2.4 Extraction and analysis

Two researchers independently extracted detailed information from the included studies using Excel 2019 software. This included basic literature details (first author’s name, publication year, country of the first author’s affiliation), basic animal information (species, weight, age, gender, sample size), treatment details (modeling method, intervention measures, dosage, route of administration, administration time, drug source, drug purity), and outcome measures. Experimental data were recorded uniformly using mean values and standard deviation (SD). If only the standard error of the mean (SEM) was provided, the original data were converted to SD based on statistical principles. Data were extracted from the images using Origin 2021 when experimental results were presented graphically. If data were missing or reports were unclear, the first author of this paper attempted to contact the study’s corresponding author. If data could not be obtained or the original data were unavailable, the first author of this paper excluded the study. After completing all data extraction, two researchers cross-checked the results. If discrepancies arose, a third researcher mediated to resolve them.

### 2.5 Risk-of-bias assessment

Two researchers independently assessed the quality of the literature using the SYRCLE animal experiment bias risk assessment tool (Hooijmans et al., 2014) in Review Manager 5.4 software, evaluating 10 items, Selection bias: Sequence generation, Baseline characteristics, and Allocation concealment, Performance bias: Random housing, and Blinding, Detection bias: Random outcome assessment, and Blinding, Attrition bias: Incomplete outcome data, Reporting bias: Selective outcome reporting, and other sources of bias. Each item was assessed as low risk (low risk, method applied correctly), unclear risk (unclear risk, method application unclear) or high risk (high risk, method applied incorrectly or not used). This process was conducted independently by two researchers and cross-checked. In case of disagreement, the two researchers discussed and decided; if no consensus was reached, a third researcher assisted in making the decision.

### 2.6 Data synthesis and analysis

Data included in the study were analyzed and summarized using Review Manager 5.4 and Stata 17.0. The study designers had already set the sample sizes to be equal between the experimental groups with different doses and the control group with a fixed dose during the experimental design. For example, the RES intervention group in the included studies had three doses (Res 10/20/40 mg/kg/day, n = 32 for each dose). In contrast, the control group received physiological saline (NS 1 mL/kg/day, n = 32). Therefore, this study only needs to consider that the experimental group includes multiple time-based subgroups, which are treated as independent studies in this analysis. The sample size of the control group for these subgroups is equal to the total control group sample size divided by the number of subgroups in the experimental group (Wu X. et al., 2022), thus avoiding artificial inflation of the sample size and improving the study’s accuracy. For example, in the abovementioned study, the RES intervention group included three doses (Res 10/20/40 mg/kg/d, with n = 32 for each dose), while the control group received physiological saline (NS 1 mL/kg/d, n = 32). The experimenters divided the periods for both the RES experimental group and the control group into four segments (3, 7, 14, and 28 days). The control group (n = 32) was evenly distributed across each time segment as a sub-control group (n = 8), and each RES experimental group with the same dose (n = 32) was also evenly distributed across each time segment as a sub-experimental group (n = 8). Consequently, this study comprises 12 subgroups, with equal sample size in each experimental (n = 8) and control (n = 8) subgroup.

All results are continuous variables. If the effect size units or measurement methods are consistent across studies, the overall effect size is compared using the mean difference (MD) and 95% confidence interval (CI). The standardized mean difference (SMD) and 95% CI are used if the effect size units or measurement methods differ across studies. Effect sizes are deemed statistically significant if *p* < 0.05. The *I* ^*2*^ or Q test assesses study heterogeneity. If *I* ^*2*^ ≤ 50% or *p* ≥ 0.1, heterogeneity is considered small, and a fixed-effect model is used; if *I* ^*2*^ > 50% or *p* < 0.1, heterogeneity is considered significant, and a random-effects model is employed, along with sensitivity and subgroup analyses to explore heterogeneity sources. When more than 10 studies are included for each outcome measure, funnel plots are generated and analyzed using Stata 17.0, with an Egger test performed. If the funnel plot shows asymmetry and the Egger’s test *p* < 0.05, the difference is regarded as significant, indicating publication bias.

## 3 Results

### 3.1 Literature selection

**FIGURE 2:**
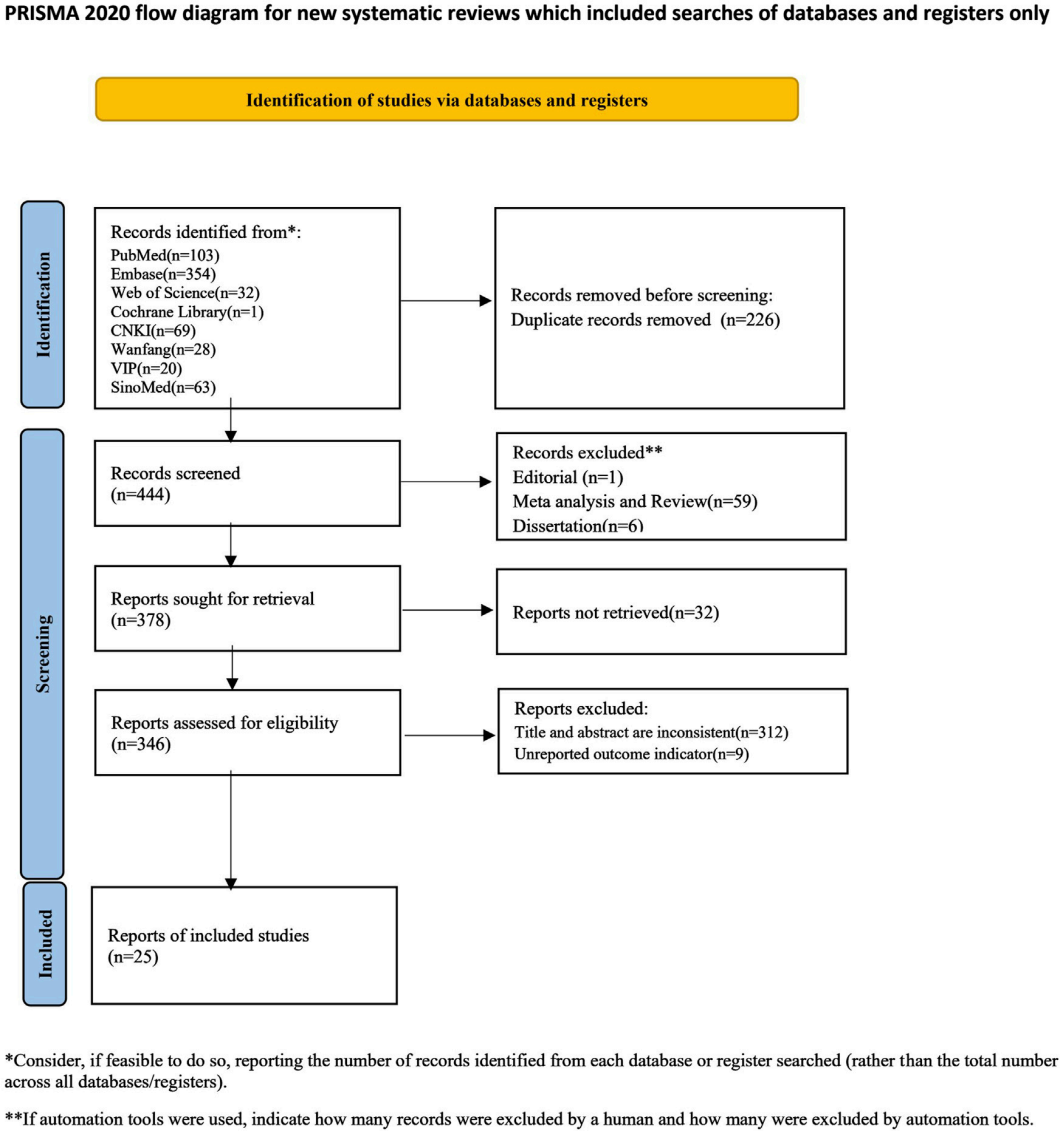
Flow diagram of the study-search process.

An initial search using the retrieval strategy yielded 670 articles, including 103 from PubMed, 354 from Embase, 32 from Web of Science, 1 from the Cochrane Library, 69 from CNKI, 28 from Wanfang, 20 from VIP, and 63 from SinoMed. After excluding 226 duplicate articles, 444 were retained. After excluding editorials, reviews, and theses, 378 articles remained. 32 articles were unavailable for full-text retrieval, 9 did not report outcome measures, and 312 were found to be inconsistent with the outcome measures after reviewing the titles, abstracts, and full texts. Ultimately, 25 articles were included, comprising 25 studies and 90 groups. Eleven studies included multiple dose groups, with six studies having three dose groups (Wang et al., 2011b; a; Zhang et al., 2011; He et al., 2012; Wang et al., 2021; Wang L. et al., 2022), and five studies having two dose groups. Eleven studies included multiple experimental time groups, with two studies including four experimental time groups (Zhang et al., 2011; Liu et al., 2013), seven studies including three experimental time groups (Cao and Mao, 2008; Wang et al., 2011b; a; He et al., 2012; Li et al., 2012; Li et al., 2015; Jin et al., 2016; Wang et al., 2021; Wang L. et al., 2022), and two studies including two experimental time groups (Jin et al., 2016; Wang L. et al., 2022). A flow chart of literature selection is shown in Figure 2.

### 3.2 Study characteristics

This meta-analysis included 628 experimental animals and 357 control animals, all rodents. In terms of animal species, all 25 studies reported on the species used. Among these, 16 studies used rats, including two types: SD and Wistar albino. Nine studies used mice, including six types: BALB/C, C57BL/6J, CD-1, ICR, Kunming, and NMRI. Regarding animal gender, 22 studies reported on gender, with 16 studies using male animals, 3 studies using female animals, and 3 studies using both male and female animals. Regarding animal age, 11 studies provided detailed reports, and 1 study reported that adult animals were selected.

The studies included in this review employed five different modeling methods. Twenty studies used bleomycin injection, three involving intraperitoneal bleomycin injection and seventeen involving tracheal bleomycin injection. In addition to bleomycin, two studies used radiation therapy, one used lipopolysaccharide tracheal injection, one used silica particle tracheal injection, and one involved exposure to fine environmental particles. Most of the RES treatment durations included in this study were within 4 weeks; however, three experimental groups exceeded this duration, with 56 days, 100 days, and 140 days. The RES used in the included studies came from 11 different manufacturers, including Xi’an Tianyi Biotechnology Co., China (n = 8), Sigma-Aldrich, United States of America (n = 4), NanoKimia Company, Iraq (n = 2), Mikrogen Pharmaceutical, Istanbul, Turkey, Shaanxi Saide Gaoke Biotechnology Co., China, Terraternal Pharmaceutical, London, England, PoliNat SL, Spain, Baoji Guokang Biotechnology Co., Ltd., China, Guangzhou Honsea Sunshine Biotech Co., Ltd., China, Chengdu Must Bio-Technology Co., Ltd., China, and Shanghai Aladdin Co., China (1 each). Three studies did not specify the manufacturers of the drugs used.

Drug dosage and administration route influence therapeutic efficacy (Wu et al., 2025). In the included studies, the RES dosage was ≤100 mg/kg/day. The 100 mg/kg/day dose was divided into three groups: low dose (0–30 mg/kg/day), medium dose (31–60 mg/kg/day), and high dose (61–100 mg/kg/day). There were four methods of RES administration: gavage (n = 16), intraperitoneal injection (n = 4), oral administration (n = 2), and tracheal injection (n = 1). The characteristics of all the included studies are summarized in Supplementary Table S2.

### 3.3 Risk of bias

**FIGURE 3:**
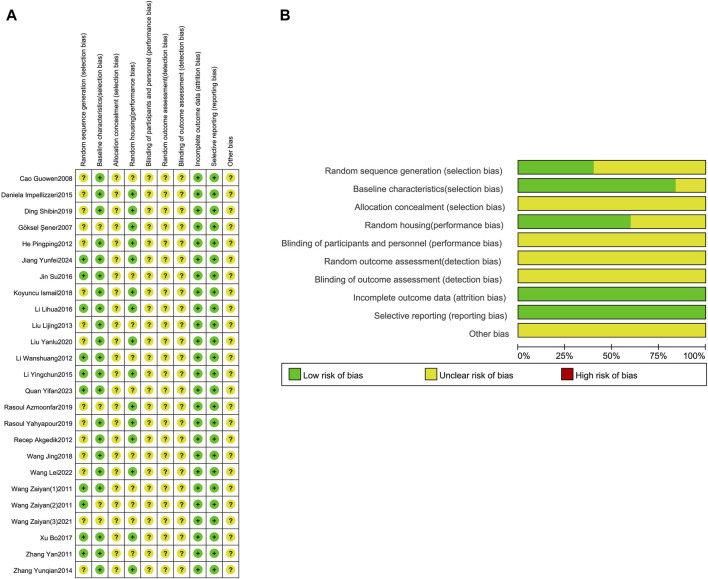
Risk of bias assessment table. Assessment of literature quality results obtained through the risk of bias by SYRCLE based on Cochrane tools. **(A)** Risk of bias summary diagram; review of authors’ judgments for each risk of bias item for each included study. **(B)** Risk of bias graph diagram; overview of authors’ judgments for each risk of bias item, expressed as a percentage of all included studies.

Assess the risk of bias and quality of all studies included based on SYRCLE’s risk of bias assessment (Figure 3). Regarding sequence generation, nine studies used random number tables to generate sequences and were rated as “low risk”; the remaining 16 studies only reported using randomization without specifying the randomization method or reporting on sequence generation and were rated as “unclear risk”. Regarding baseline characteristics, 21 studies had identical baseline characteristics between the experimental and control groups and were rated as “low risk”; the remaining four studies did not describe the characteristics of the animals included in the study and were rated as “unclear risk”. Regarding allocation concealment, 25 studies provided insufficient information, making it impossible to determine the risk, and were rated as “unclear risk”. Regarding animal placement randomization, 15 studies had identical housing conditions and environments for animals in the experimental and control groups, rated as “low risk”; 10 studies did not describe this, rated as “unclear risk”. Regarding blinding and randomization for animal caretakers, researchers, and outcome evaluators, 25 studies provided insufficient information to judge, rated as “unclear risk”. Regarding incomplete data reporting and selective reporting, none of the 25 studies had data loss or selective reporting, so both aspects were rated as “low risk”. In addition to the above bias risk assessments, it is unclear whether other biases exist, so all 25 studies were rated as “unclear risk” in terms of different biases.

### 3.4 Core outcome measures for reversing the fibrosis process

#### 3.4.1 Block the core pathological process

##### 3.4.1.1 Reversing the pathology of fibrosis

###### 3.4.1.1.1 Reduction of pulmonary fibrosis score

**FIGURE 4:**
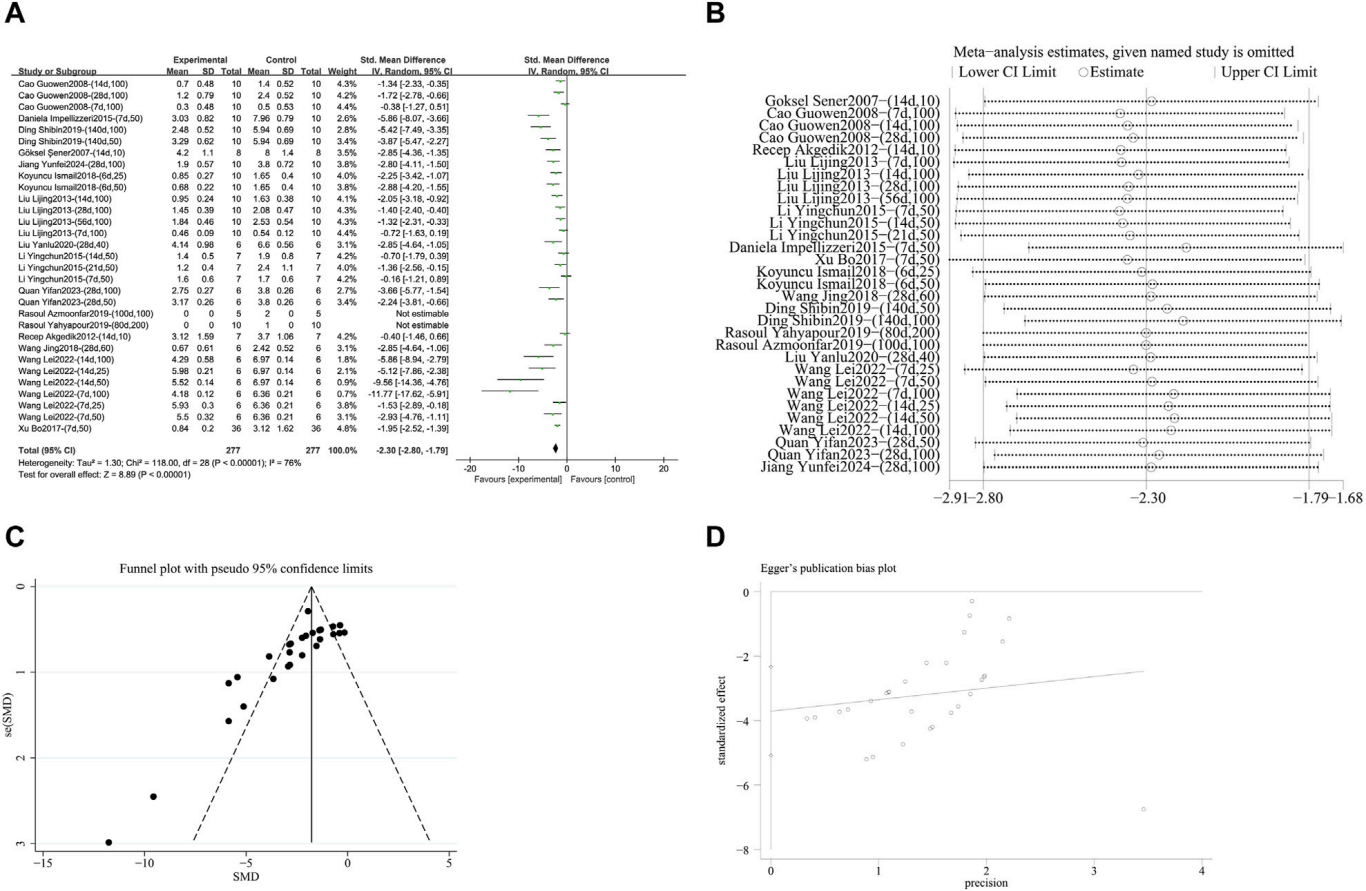
Effect of RES on Pulmonary fibrosis score in lung tissues of PF animals. **(A)** Forest plot of the Pulmonary fibrosis score. **(B)** Sensitivity analysis of the Pulmonary fibrosis score. **(C)** Funnel plot of the Pulmonary fibrosis score. **(D)** Egger test for the Pulmonary fibrosis score.

A meta-analysis was conducted on the effect sizes of pulmonary fibrosis histological scores in 31 studies from 16 literature sources. The study samples were obtained from bronchoalveolar lavage fluid, lung tissue or serum. Compared with the control group, RES significantly reduced the histological score of pulmonary fibrosis. There was significant heterogeneity among the included studies, and a random-effects model was used (SMD = −2.30, 95% CI [−2.80, −1.79], *p* < 0.00001, *I* ^*2*^ = 76%) (Figure 4A). Sensitivity analysis indicated that the results were stable (Figure 4B). Further subgroup analysis revealed that animal species (*p* = 0.86), RES dosage (*p* = 0.83), and RES intervention duration (*p* = 0.61) were not sources of heterogeneity. However, animal strain (*p* < 0.0002, *I* ^*2*^ = 79%) (Supplementary Figure S1A), RES drug source (*p* < 0.0001, *I* ^*2*^ = 86%) (Supplementary Figure S2B), pulmonary fibrosis modeling method (*p* < 0.009, *I* ^*2*^ = 79%) (Supplementary Figure S1C), and RES administration route (*p* < 0.004, *I* ^*2*^ = 77.8%) (Supplementary Figure S3D) were sources of heterogeneity in this study. Additionally, funnel plots (Figure 4C) and Egger’s test (*p* < 0.0001) (Figure 4D) indicated the presence of publication bias.

###### 3.4.1.1.2 Reduciton of hydroxyproline (hyp) deposition

**FIGURE 5:**
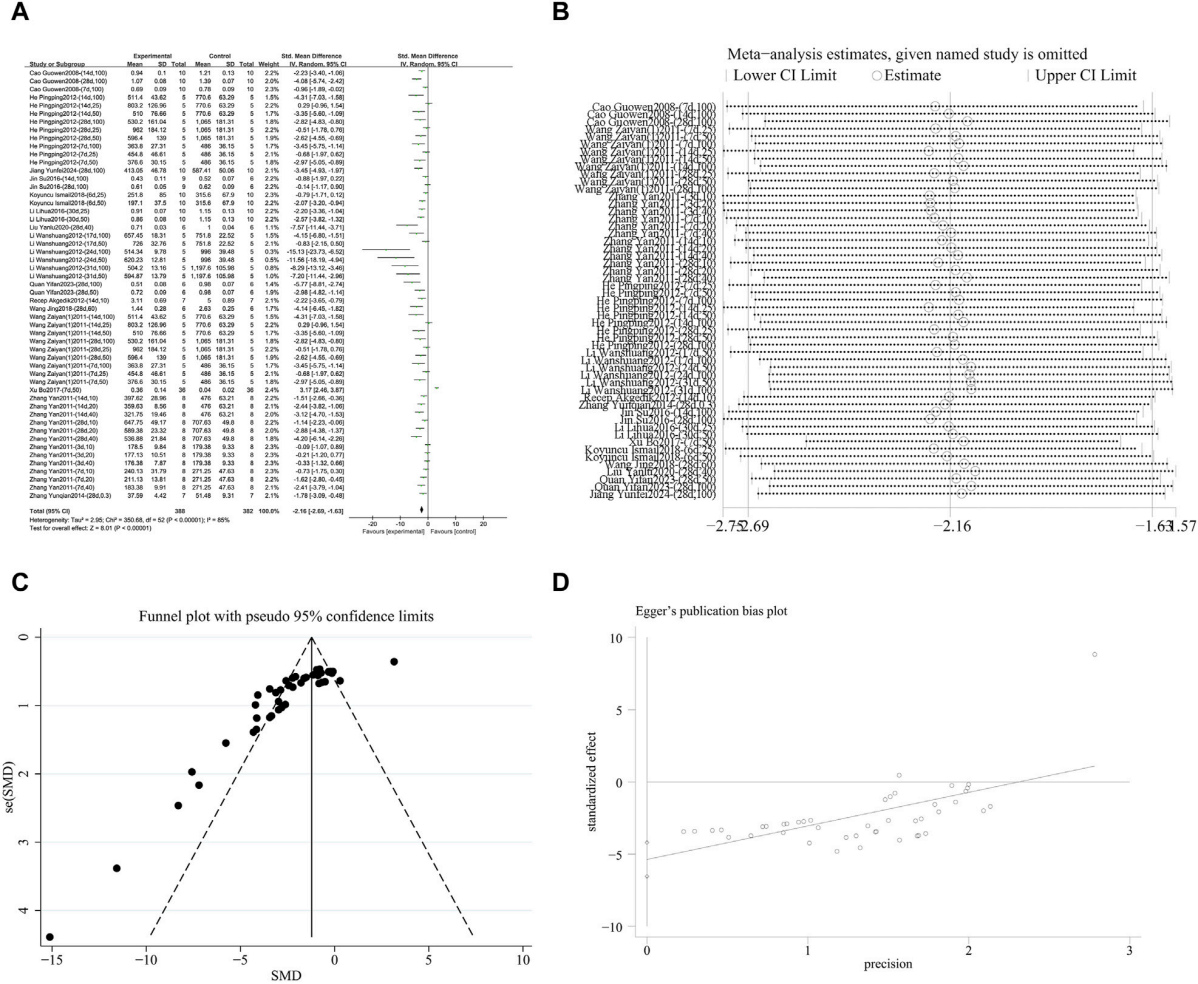
Effect of RES on Hyp content in lung tissues of PF animals. **(A)** Forest plot of Hyp content. **(B)** Sensitivity analysis of Hyp content. **(C)** Funnel plot of Hyp content. **(D)** Egger test for Hyp content.

A meta-analysis was conducted on the effect sizes of Hyp content in 53 studies from 15 literature sources, the study samples were obtained from bronchoalveolar lavage fluid, lung tissue or serum. Compared with the control group, RES significantly reduced Hyp content. There was significant heterogeneity among the included studies, and a random-effects model was used (SMD = −2.16, 95% CI [−2.69, −1.63], *p* < 0.00001, *I* ^*2*^ = 85%) (Figure 5A). Sensitivity analysis indicated that the results were stable (Figure 5B). Further subgroup analysis revealed that animal species (*p* = 0.22), animal strain (*p* = 0.29), lung fibrosis modeling method (*p* = 0.31), and RES administration route (*p* = 0.11) were not sources of heterogeneity. However, RES drug source (*p* < 0.0001, *I* ^*2*^ = 78.9%) (Supplementary Figure S2A), RES dosage (*p* < 0.0001, *I* ^*2*^ = 90.1%) (Supplementary Figure S2B), and RES intervention duration (*p* = 0.01, *I* ^*2*^ = 76.3%) (Supplementary Figure S2C) were the sources of heterogeneity in this study. Additionally, funnel plots (Figure 5C) and Egger’s test (*p* < 0.0001) (Figure 5D) indicated the presence of publication bias.

###### 3.4.1.1.3 Inhibition of type I collagen (col 1) accumulation

A meta-analysis was conducted on the effect sizes of Col 1 content in 11 studies from 6 literature sources, the study samples were obtained from lung tissue or serum. Compared with the control group, RES significantly reduced the content of Col 1. There was significant heterogeneity among the included studies, and a random-effects model was used (SMD = −2.70, 95% CI [−4.71, −0.70], *p* < 0.00001, *I* ^*2*^ = 96%) (Supplementary Figure S3A). Sensitivity analysis indicated that the results were stable (Supplementary Figure S3B). Further subgroup analysis showed that animal species (*p* = 0.39), animal strain (*p* = 0.39), RES dosage (*p* = 0.55), RES intervention time (*p* = 0.13), pulmonary fibrosis modeling method (*p* = 0.66), and RES administration route (*p* = 0.37) were not sources of heterogeneity. RES drug source (*p* = 0.03, *I* ^*2*^ = 67.9%) (Supplementary Figure S4) was the source of heterogeneity in this study. Additionally, funnel plots (Supplementary Figure S3C) and Egger’s test (*p* = 0.003) (Supplementary Figure S3D) indicated the presence of publication bias.

##### 3.4.1.2 Inhibition of inflammatory damage

###### 3.4.1.2.1 Alleviate alveolitis score

**FIGURE 6:**
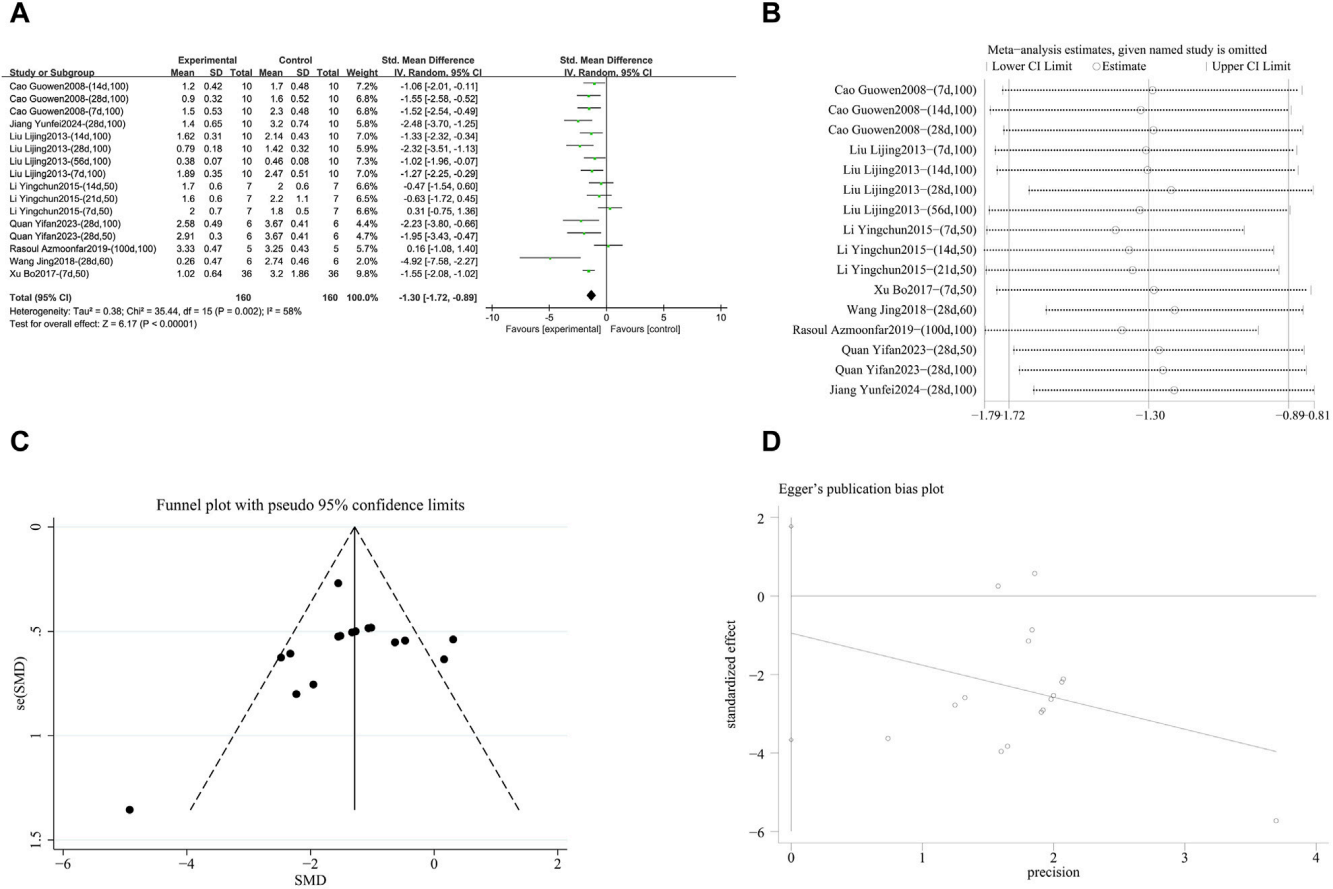
Effect of RES on Alveolitis score in lung tissues of PF animals. **(A)** Forest plot of the Alveolitis score. **(B)** Sensitivity analysis of the Alveolitis score. **(C)** Funnel plot of the Alveolitis score. **(D)** Egger test for the Alveolitis score.

A meta-analysis was conducted on the effect sizes of alveolitis scores in 16 studies from 8 literature sources, the study samples were obtained from lung tissue or serum. Compared with the control group, RES significantly reduced alveolitis scores. There was significant heterogeneity among the included studies, and a random-effects model was used (SMD = −1.30, 95% CI [−1.72, −0.89], *p* = 0.002, *I* ^*2*^ = 58%) (Figure 6A). Sensitivity analysis indicated that the results were stable (Figure 6B). Further subgroup analysis revealed that animal species (*p* = 0.97), RES dosage (*p* = 0.65), and RES intervention duration (*p* = 0.18) were not sources of heterogeneity. However, animal strain (*p* = 0.03, *I* ^*2*^ = 66.2%) (Supplementary Figure S5A), RES drug source (*p* = 0.002, *I* ^*2*^ = 79.7%) (Supplementary Figure S5B), pulmonary fibrosis modeling method (*p* = 0.03, *I* ^*2*^ = 72.7%) (Supplementary Figure S5C), and RES administration route (*p* = 0.006, *I* ^*2*^ = 86.8%) (Supplementary Figure S5D) were sources of heterogeneity in this study. Additionally, the funnel plot (Figure 6C) symmetry was high, and the Egger’s test (*p* = 0.468) (Figure 6D) indicated no publication bias.

#### 3.4.2 Target key molecular mechanisms

##### 3.4.2.1 Inhibition of pro-fibrotic factors

###### 3.4.2.1.1 Blocking the TGF-β signaling pathway

**FIGURE 7:**
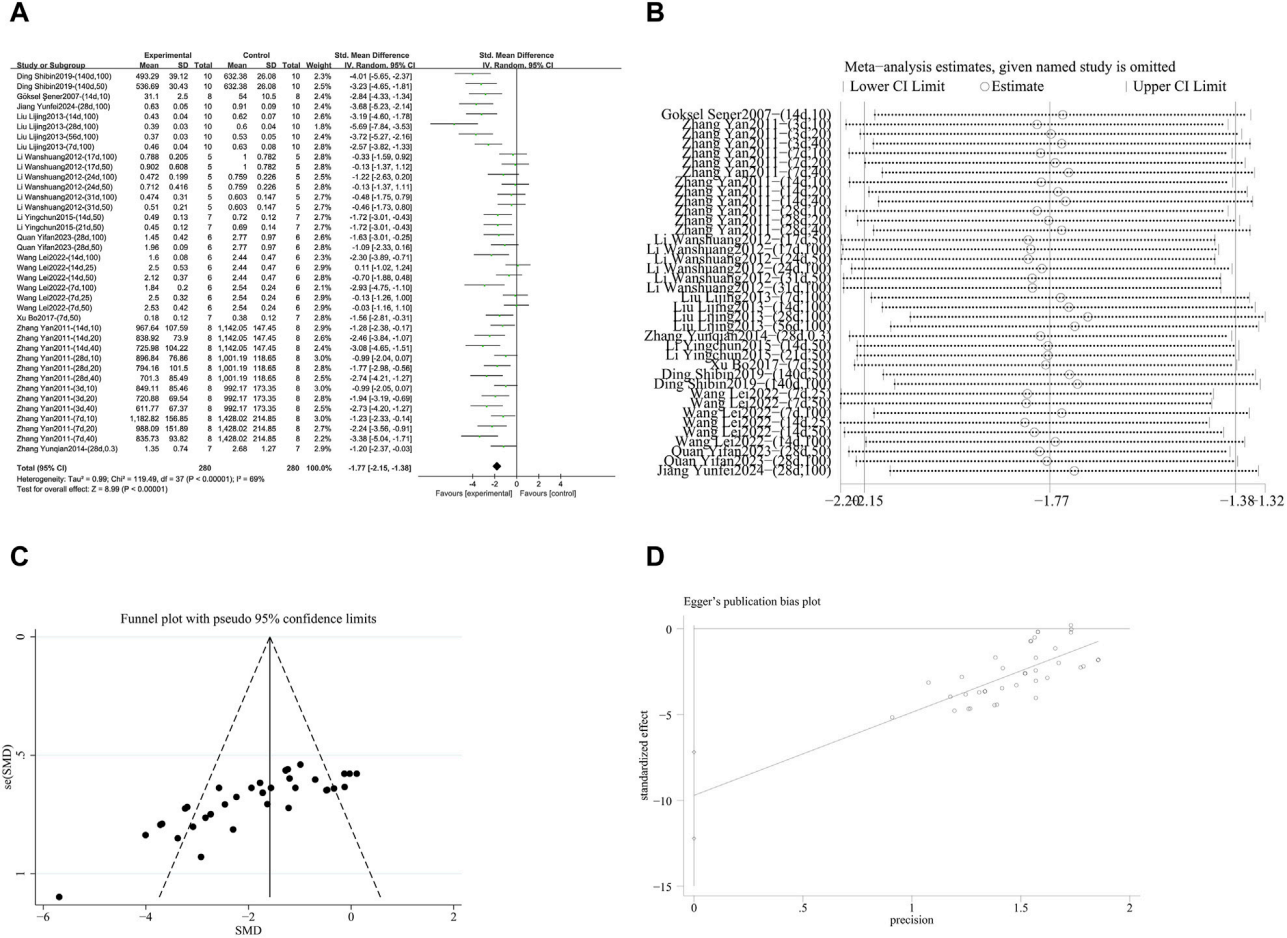
Effect of RES on TGF-β content in lung tissues of PF animals. **(A)** Forest plot of TGF-β content. **(B)** Sensitivity analysis of TGF-β content. **(C)** Funnel plot of TGF-β content. **(D)** Egger test for TGF-β content.

A meta-analysis was conducted on the effect sizes of TGF-β content in 38 studies from 11 literature sources, the study samples were obtained from bronchoalveolar lavage fluid, lung tissue or serum. Compared with the control group, RES significantly reduced TGF-β content. There was significant heterogeneity among the included studies, and a random-effects model was used (SMD = −1.77, 95% CI [−2.15, −1.38], *p* < 0.00001, *I* ^*2*^ = 69%) (Figure 7A). Sensitivity analysis indicated that the results were stable (Figure 7B). Further subgroup analysis revealed that animal species (*p* = 0.07), RES drug source (*p* = 0.13), RES dosage (*p* = 0.05), RES intervention duration (*p* = 0.74), and RES administration route (*p* = 0.38) were not sources of heterogeneity. Animal strain (*p* = 0.0002, *I* ^*2*^ = 84.7%) (Supplementary Figure S6A) and pulmonary fibrosis modeling method (*p* = 0.006, *I* ^*2*^ = 76.1%) (Supplementary Figure S6B) contributed to the heterogeneity in this study. Additionally, funnel plots (Figure 7C) and Egger’s test (*p* < 0.0001) (Figure 7D) indicated the presence of publication bias.

###### 3.4.2.1.2 Inhibition of NF-κB activation

**FIGURE 8:**
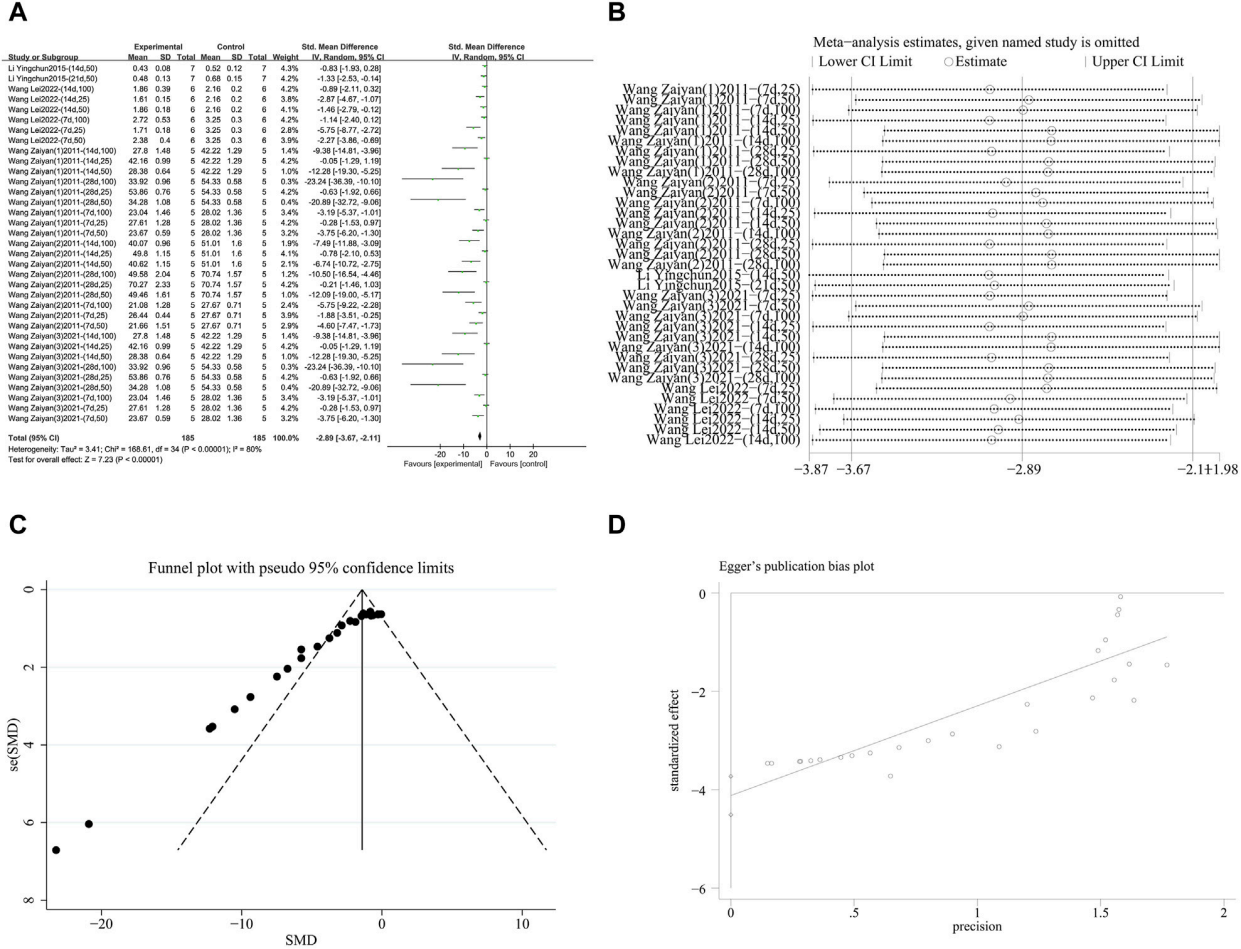
Effect of RES on NF-κB content in lung tissues of PF animals. **(A)** Forest plot of NF-κB content. **(B)** Sensitivity analysis of NF-κB content. **(C)** Funnel plot of NF-κB content. **(D)** Egger test for NF-κB content.

A meta-analysis was conducted on the effect sizes of NF-κB content in 35 studies from 5 literature sources, all study samples were obtained from lung tissue. Compared with the control group, RES significantly reduced NF-κB content. There was significant heterogeneity among the included studies, and a random-effects model was used (SMD = −2.89, 95% CI [−3.67, −2.11], *p* < 0.00001, *I* ^*2*^ = 80%) (Figure 8A). Sensitivity analysis indicated that the results were stable (Figure 8B). Further subgroup analysis revealed that the animal species, strains, and lung fibrosis modeling methods were consistent across 35 studies. The duration of RES intervention (*p* = 0.34) and route of RES administration (*p* = 0.08) were not sources of heterogeneity. The source of RES drug source (*p* = 0.09, *I* ^*2*^ = 64.9%) (Supplementary Figure S7A) and RES dosage (*p* < 0.00001, *I* ^*2*^ = 93.5%) (Supplementary Figure S7B) were sources of heterogeneity in this study. Additionally, funnel plots (Figure 8C) and Egger’s test (*p* < 0.0001) (Figure 8D) indicated publication bias.

##### 3.4.2.2 Neutralize pro-inflammatory cytokines

###### 3.4.2.2.1 Reduction of TNF-α levels

**FIGURE 9:**
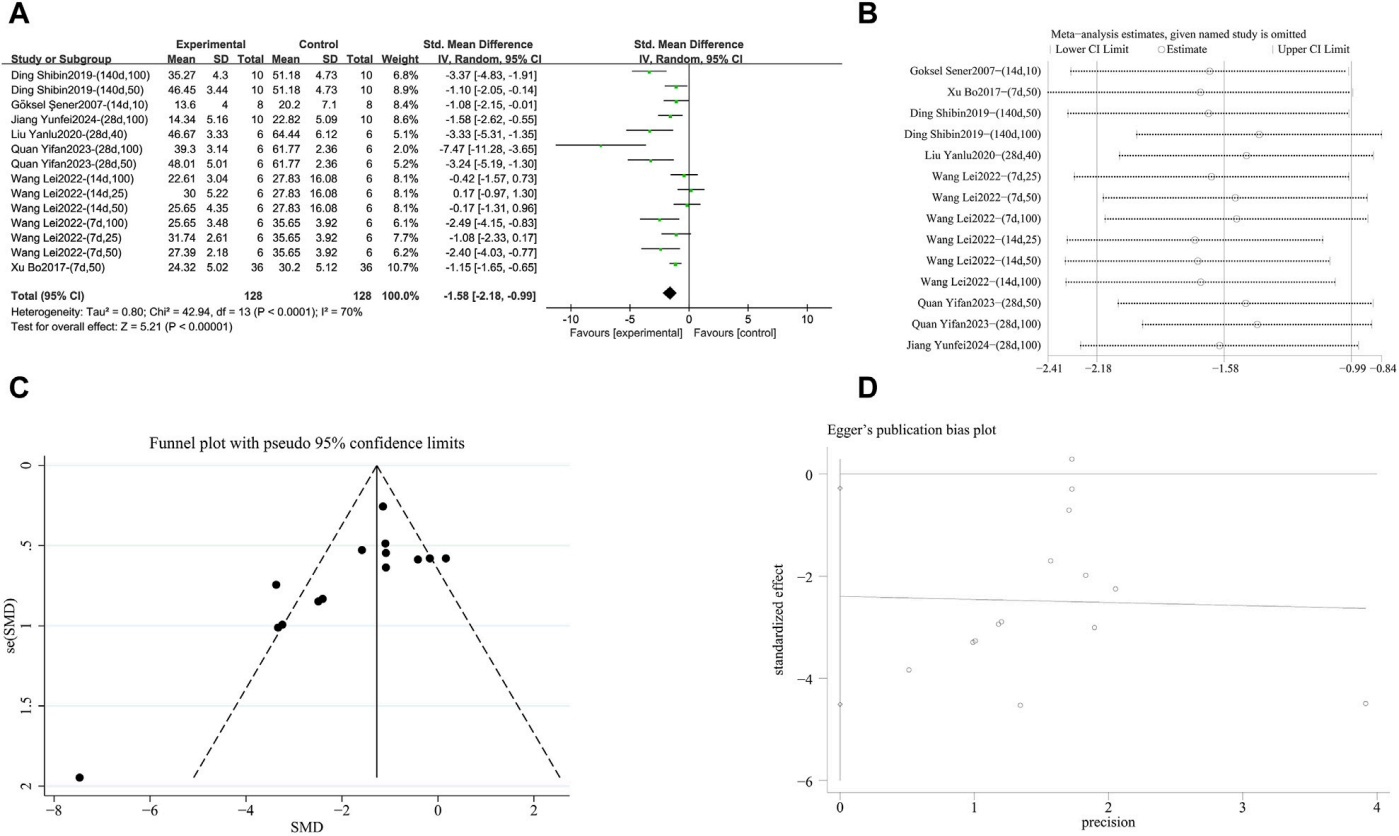
Effect of RES on TNF-α content in lung tissues of PF animals. **(A)** Forest plot of TNF-α content. **(B)** Sensitivity analysis of TNF-α content. **(C)** Funnel plot of TNF-α content. **(D)** Egger test for TNF-α content.

A meta-analysis was conducted on the effect sizes of TNF-α content in 14 studies from 7 literature sources, the study samples were obtained from bronchoalveolar lavage fluid, lung tissue or serum. Compared with the control group, RES significantly reduced TNF-α content. There was significant heterogeneity among the included studies, and a random-effects model was used (SMD = −1.58, 95% CI [−2.18, −0.99], *p* < 0.00001, *I* ^*2*^ = 70%) (Figure 9A). Sensitivity analysis indicated that the results were stable (Figure 9B). Further subgroup analysis revealed no significant differences based on animal species (*p* = 0.57), animal strain (*p* = 0.59), RES drug source (*p* = 0.11), RES dosage (*p* = 0.08), RES intervention duration (*p* = 0.84), pulmonary fibrosis modeling method (*p* = 0.13), and RES administration route (*p* = 0.13) were not sources of heterogeneity, and the cause of this study remains unclear. Additionally, funnel plots (Figure 9C) and Egger’s test (*p* = 0.03) (Figure 9D) indicated the presence of publication bias.

###### 3.4.2.2.2 Inhibition of IL-1β release

**FIGURE 10:**
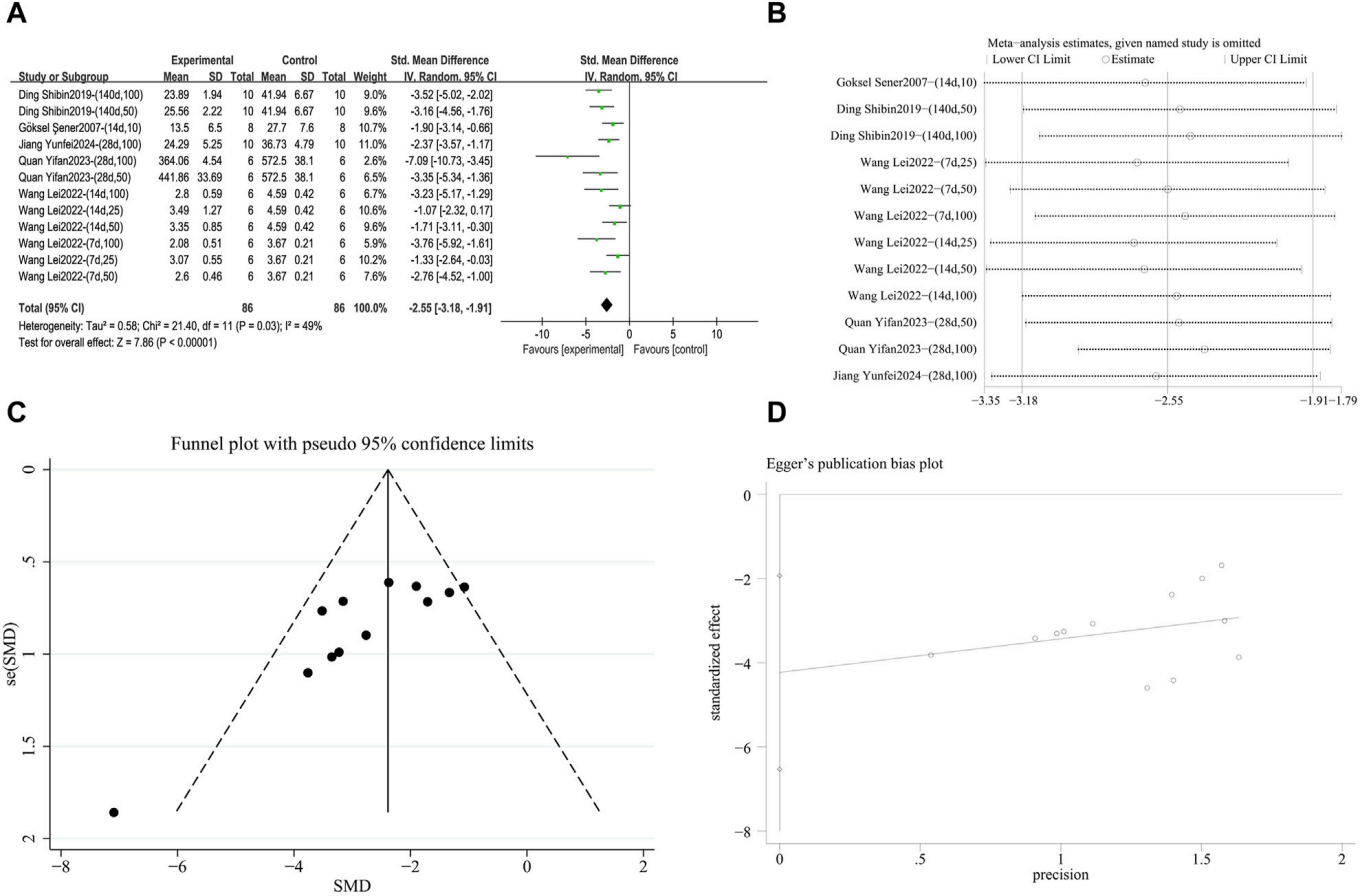
Effect of RES on IL-1β content in lung tissues of PF animals. **(A)** Forest plot of IL-1β content. **(B)** Sensitivity analysis of IL-1β content. **(C)** Funnel plot of IL-1β content. **(D)** Egger test for IL-1β content.

A meta-analysis was conducted on the effect sizes of IL-1β content in 12 studies from 5 literature sources (Şener et al., 2007; Impellizzeri et al., 2015; Wang L. et al., 2022; Quan et al., 2023; Jiang et al., 2024), the study samples were obtained from bronchoalveolar lavage fluid or lung tissue. Compared with the control group, RES reduced IL-1β content. There was significant heterogeneity among the included studies, and a random-effects model was still used (SMD = −2.55, 95% CI [−3.18, −1.91], *p* = 0.03, *I* ^*2*^ = 49%) (Figure 10A). Sensitivity analysis indicated that the results were stable (Figure 10B). Further subgroup analysis showed that animal species (*p* = 0.38), animal strain (*p* = 0.39), RES drug source (*p* = 0.10), RES intervention duration (*p* = 0.36), and RES administration route (*p* = 0.22) were not sources of heterogeneity. RES dosage (*p* = 0.006, *I* ^*2*^ = 80.5%) (Supplementary Figure S8A) and pulmonary fibrosis modeling method (*p* = 0.04, *I* ^*2*^ = 69.7%) (Supplementary Figure S8B) contributed to the heterogeneity in this study. Additionally, funnel plots (Figure 10C) and the Egger’s test (*p* = 0.002) (Figure 10D) indicated the presence of publication bias.

###### 3.4.2.2.3 Downregulation of IL-6 expression

**FIGURE 11:**
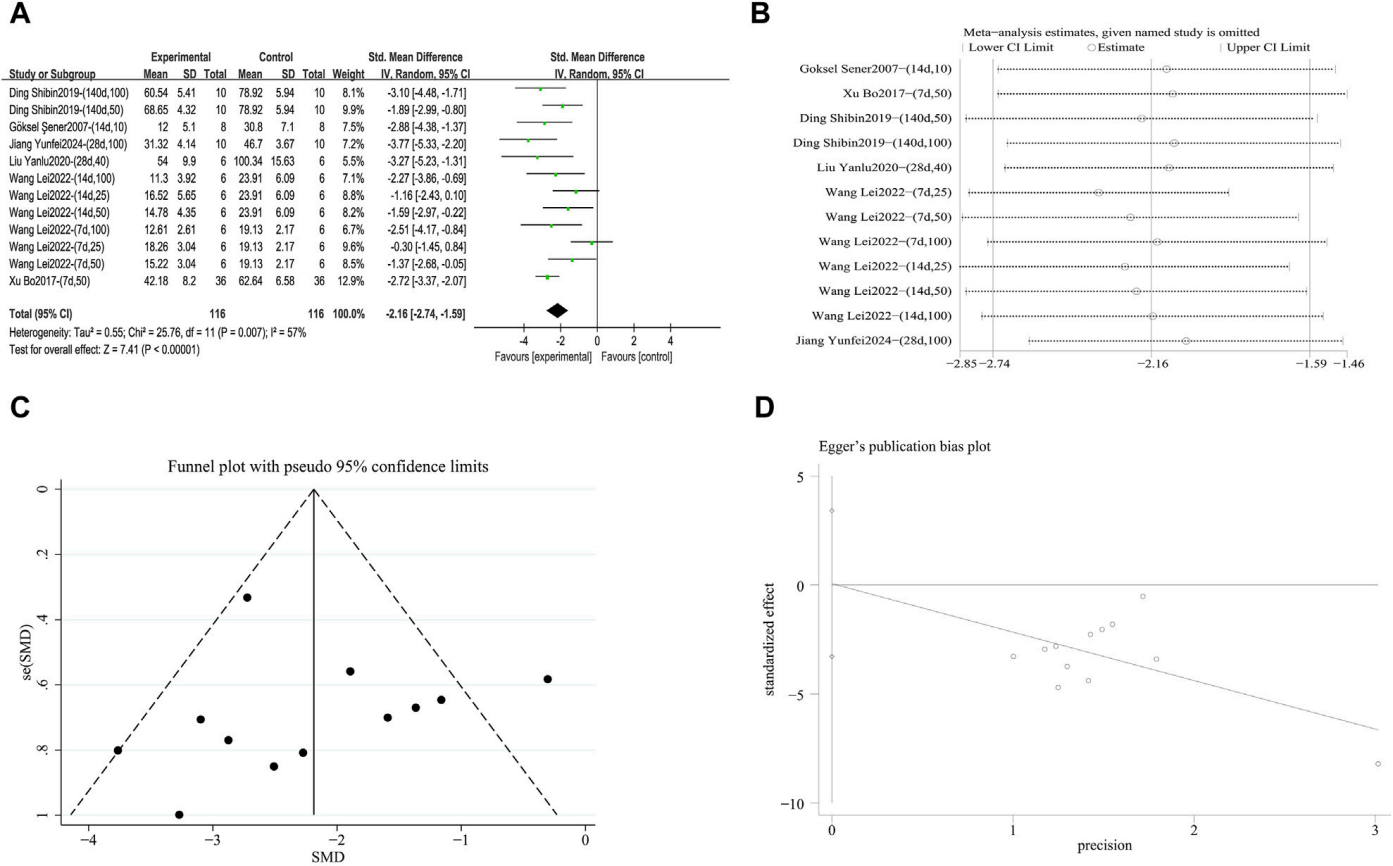
Effect of RES on IL-6 content in lung tissues of PF animals. **(A)** Forest plot of IL-6 content. **(B)** Sensitivity analysis of IL-6 content. **(C)** Funnel plot of IL-6 content. **(D)** Egger test for IL-6 content.

A meta-analysis was conducted on the effect sizes of IL-6 content in 12 studies from 6 literature sources, the study samples were obtained from bronchoalveolar lavage fluid, lung tissue or serum. Compared with the control group, RES significantly reduced IL-6 content. Significant heterogeneity was among the included studies, and a random-effects model was used (SMD = −2.16, 95% CI [−2.74, −1.59], *p* = 0.007, *I* ^*2*^ = 57%) (Figure 11A). Sensitivity analysis indicated that the results were stable (Figure 11B). Further subgroup analysis revealed that animal species (*p* = 0.19), animal strain (*p* = 0.24), RES dosage (*p* = 0.12), RES intervention duration (*p* = 0.66), and pulmonary fibrosis modeling method (*p* = 0.65) were not sources of heterogeneity. RES drug source (*p* = 0.03, *I* ^*2*^ = 62.3%) (Supplementary Figure S9A) and RES administration route (*p* = 0.004, *I* ^*2*^ = 81.7%) (Supplementary Figure S9B) contributed to the heterogeneity in this study. Additionally, the funnel plot (Figure 11C) symmetry was high, and the Egger’s test (*p* = 0.966) (Figure 11D) indicated no publication bias.

##### 3.4.2.3 Regulation of oxidative stress factor

###### 3.4.2.3.1 Reduction of MDA lipid peroxidation

A meta-analysis was conducted on the effect sizes of MDA content in 11 studies from 8 literature sources, the study samples were obtained from bronchoalveolar lavage fluid, lung tissue or serum. Compared with the control group, RES significantly reduced MDA content. There was significant heterogeneity among the included studies, and a random-effects model was used (SMD = −2.20, 95% CI [−2.87, −1.53], *p* < 0.0001, *I* ^*2*^ = 57%) (Supplementary Figure S10A). Sensitivity analysis indicated that the results were stable (Supplementary Figure S10B). Further subgroup analysis showed no significant differences in animal species (*p* = 0.28), animal strain (*p* = 0.31), RES drug source (*p* = 0.38), RES dosage (*p* = 0.35), RES intervention duration (*p* = 0.80), pulmonary fibrosis modeling method (*p* = 0.10), and RES administration route (*p* = 0.57) were not sources of heterogeneity, so the cause of this study remains unclear. Additionally, funnel plots (Supplementary Figure S10C) and Egger’s test (*p* < 0.0001) (Supplementary Figure S10D) indicated the presence of publication bias.

###### 3.4.2.3.2 Inhibition of MPO activity

A meta-analysis was conducted on the effect sizes of MPO content in 5 studies from 4 literature sources, the study samples were obtained from bronchoalveolar lavage fluid, lung tissue or serum. Compared with the control group, RES significantly reduced MPO content. There was significant heterogeneity among the included studies, and a random-effects model was used (SMD = −2.22, 95% CI [−3.09, −1.35], *p* = 0.06, *I* ^*2*^ = 55%) (Supplementary Figure S11A). Sensitivity analysis indicated that the results were stable (Supplementary Figure S11B). Further subgroup analysis revealed that the lung fibrosis modeling methods were consistent across five studies. There were no significant differences in animal species (*p* = 0.44), animal strain (*p* = 0.16), RES drug source (*p* = 0.16), RES dosage (*p* = 0.05), RES intervention duration (*p* = 0.44), and RES administration route (*p* = 0.30) were not sources of heterogeneity. Therefore, the cause of this study remains unclear. Additionally, the number of studies was <10, so no publication bias test was conducted.

###### 3.4.2.3.3 Enhance SOD antioxidant capacity

**FIGURE 12:**
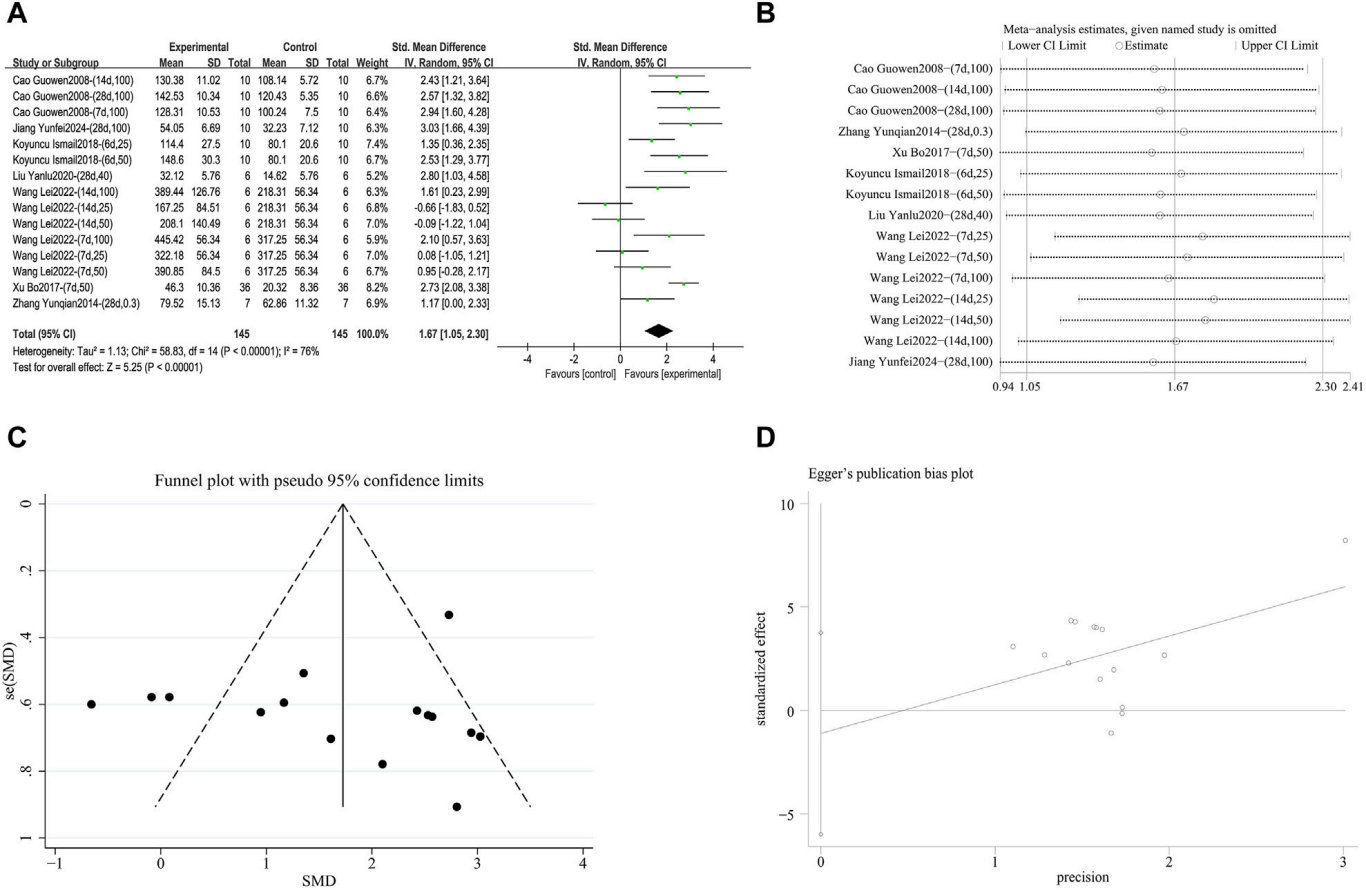
Effect of RES on SOD content in lung tissues of PF animals. **(A)** Forest plot of SOD content. **(B)** Sensitivity analysis of SOD content. **(C)** Funnel plot of SOD content. **(D)** Egger test for SOD content.

A meta-analysis was conducted on the effect sizes of SOD content in 15 studies from 7 literature sources, the study samples were obtained from lung tissue or serum. Compared with the control group, RES significantly increased SOD content. There was significant heterogeneity among the included studies, and a random-effects model was used (SMD = 1.67, 95% CI [1.05, 2.30], *p* < 0.0001, *I* ^*2*^ = 76%) (Figure 12A). Sensitivity analysis indicated that the results were stable (Figure 12B). Further subgroup analysis revealed that animal species (*p* = 0.08), animal strain (*p* = 0.05), RES intervention duration (*p* = 0.69), and lung fibrosis modeling method (*p* = 0.43) were not sources of heterogeneity. However, RES drug source (*p* < 0.001, *I* ^*2*^ = 86.4%) (Supplementary Figure S12A), RES dosage (*p* = 0.002, *I* ^*2*^ = 84.4%) (Supplementary Figure S12B), and RES administration route (*p* < 0.001, *I* ^*2*^ = 91.1%) (Supplementary Figure S12C) were the sources of heterogeneity in this study. Additionally, the funnel plot (Figure 12C) symmetry was high, and the Egger test (*p* = 0.631) (Figure 12D) indicated no publication bias.

## 4 Discussion

PF is a chronic, progressive, fibrotic interstitial lung disease characterized by increasing scarring of the lung interstitium (Somogyi et al., 2019). Recently, there have been significant advances in treating PF. Pirfenidone and nintedanib are commonly used in clinical practice as antifibrotic therapies (Karimi-Shah and Chowdhury, 2015). While these medications effectively slow lung function decline and disease progression (Biondini et al., 2020), long-term use can cause severe gastrointestinal reactions and liver damage (Kou et al., 2024). Therefore, it is essential to explore new treatment options for PF. Preclinical systematic reviews and meta-analyses of animal studies are valuable tools for assessing clinical efficacy. By analyzing the safety and effectiveness of animal study results, these findings can be further applied to human clinical trials (de Vries et al., 2014). Although numerous preclinical studies have investigated RES in animal models of PF, comprehensive summaries of these studies are limited or incomplete. Additionally, some published articles on the mechanisms of RES treatment for PF lack extensive experimental evidence. A systematic review grounded in evidence-based medicine can help improve animal research quality and facilitate RES therapy translation for PF from preclinical studies to clinical applications.

### 4.1 Summary of results

To our knowledge, no previous meta-analysis has quantitatively evaluated the efficacy of RES therapy for pulmonary fibrosis (PF). This meta-analysis included 25 studies, 90 experimental groups, with 628 experimental animals and 357 control animals. RES significantly improved pulmonary fibrosis, reduced inflammation, and decreased oxidative levels. The results suggest that RES exerts its anti-fibrotic effects through multiple mechanisms, providing scientific support for the clinical use of RES in treating pulmonary fibrosis (Table 2).

**TABLE 2:** Summary of results.

Outcome			Result	References
Core pathological process	Fibrosis-related	Pulmonary fibrosis score	SMD = −2.30, 95% CI [−2.80, −1.79], *p* < 0.00001, *I* ^*2*^ = 76%	Şener et al. (2007), Cao and Mao (2008), Akgedik et al. (2012), Liu et al. (2013), Impellizzeri et al. (2015), Li et al. (2015), Xu et al., 2017; Ismail (2018), Wang et al. (2018), Ding et al. (2019), Yahyapour et al. (2019), Azmoonfar et al. (2020), Liu et al. (2020), Wang et al. (2022a), Quan et al. (2023), Jiang et al. (2024)
		Hyp content	SMD = −2.16, 95% CI [-2.69, −1.63], *p* < 0.00001, *I* ^*2*^ = 85%	Cao and Mao (2008), Wang et al. (2011b), Zhang et al. (2011), Akgedik et al. (2012), He et al. (2012), Li et al. (2012), Zhang et al. (2015), Jin et al. (2016), Li et al. (2016), Xu et al., 2017; Ismail (2018), Wang J. et al., 2018, Liu et al. (2020), Quan et al. (2023), Jiang et al. (2024)
		Col 1 content	SMD = −2.70, 95% CI [-4.71, −0.70], *p* < 0.00001, *I* ^*2*^ = 96%	Liu et al. (2013), Zhang et al. (2015), Li et al. (2016), Xu et al. (2017), Liu et al. (2020), Quan et al. (2023)
	Inflammation-related	Alveolitis score	SMD = −1.30, 95% CI [−1.72, −0.89], *p* = 0.002, *I* ^*2*^ = 58%	Cao and Mao (2008), Liu et al. (2013), Li et al. (2015), Xu et al., 2017; Wang J. et al., 2018, Azmoonfar et al. (2020), Quan et al. (2023), Jiang et al. (2024)
Key molecular mechanism	Pro-fibrotic factor	TGF-β content	SMD = −1.77, 95% CI [−2.15, −1.38], *p* < 0.00001, *I* ^*2*^ = 69%	Şener et al. (2007), Zhang et al. (2011), Li et al. (2012), Liu et al. (2013), Li et al. (2015), Zhang et al. (2015), Xu et al. (2017), Ding et al. (2019), Wang et al. (2022a), Quan et al. (2023), Jiang et al. (2024)
		NF-κB content	SMD = −2.89, 95% CI [−3.67, −2.11], *p* < 0.00001, *I* ^*2*^ = 80%	Wang et al. (2011b), Li et al. (2015), Wang et al. (2021), Wang et al. (2022a)
	Pro-inflammatory cytokine	TNF-α content	SMD = −1.58, 95% CI [−2.18, −0.99], *p* < 0.00001, *I* ^*2*^ = 70%	Şener et al. (2007), Xu et al. (2017), Ding et al. (2019), Liu et al. (2020), Wang et al. (2022a), Quan et al. (2023), Jiang et al. (2024)
		IL-1β content	SMD = −2.55, 95% CI [−3.18, −1.91], *p* = 0.03, *I* ^*2*^ = 49%	Şener et al. (2007), Impellizzeri et al. (2015), Wang et al. (2022a), Quan et al. (2023), Jiang et al. (2024)
		IL-6 content	SMD = −2.16, 95% CI [−2.74, −1.59], *p* = 0.007, *I* ^*2*^ = 57%	Şener et al. (2007), Xu et al. (2017), Ding et al. (2019), Liu et al. (2020), Wang et al. (2022a), Jiang et al. (2024)
	Oxidative stress factor	MDA content	SMD = −2.20, 95% CI [−2.87, −1.53], *p* < 0.0001, *I* ^*2*^ = 57%	Şener et al. (2007), Cao and Mao (2008), Akgedik et al. (2012), Zhang et al. (2015), Xu et al., 2017; Ismail (2018), Liu et al. (2020), Jiang et al. (2024)
		MPO content	SMD = −2.22, 95% CI [−3.09, −1.35], *p* = 0.06, *I* ^*2*^ = 55%	Şener et al. (2007), Impellizzeri et al. (2015), Ismail, 2018; Liu et al. (2020)
		SOD content	SMD = 1.67, 95% CI [1.05, 2.30], *p* < 0.0001, *I* ^*2*^ = 76%	Cao and Mao (2008), Zhang et al. (2015), Xu et al., 2017; Ismail (2018), Liu et al. (2020), Wang et al. (2022a), Jiang et al. (2024)

### 4.2 Heterogeneity analysis

Differences in animal species, strains, RES drug sources, RES dosage, RES intervention duration, pulmonary fibrosis modeling methods, and RES administration routes may cause clinical heterogeneity. Before formal analysis, we addressed potential clinical heterogeneity from two perspectives. First, regarding RES dosage, the range in this experiment was 0–100 mg/kg/day. Based on low, medium, and high dosage groups, the dosage was categorized as follows: 0–30 mg/kg/day for low dose, 31–60 mg/kg/day for medium dose, and 61–100 mg/kg/day for high dose; second, regarding the intervention duration, the categories were: ≤7 days (short-term), 8–28 days (medium-term), and >28 days (long-term). The study found that heterogeneity was generally high across all outcome measures. Therefore, sensitivity analysis was first performed to confirm the stability of the results, followed by subgroup analysis to identify sources of heterogeneity, which mainly originated from RES drug sources, lung fibrosis modeling methods, RES administration routes, and RES dosage. Although this study included many subgroups, the sources of heterogeneity for TNF-α, MDA, and MPO remain unclear. We think that inconsistent testing methods, varied sampling times, and different drug sources may all add to this heterogeneity. However, because of the limited number of studies included, we cannot perform a more detailed analysis with the current data. Future research should adopt a standardized set of core outcome measures (such as those from the COMET Initiative) to unify testing procedures and require detailed reporting of drug purity, assay kit numbers, and sampling times. Doing so will help clarify differences between studies in future research, improving the reliability and clinical relevance of the findings.

### 4.3 Interpretation and discussion of the study results

#### 4.3.1 Selection of animal models

Since PF is difficult to replicate, various animals have been used to develop PF animal models. These models from different species can better reflect PF patients (Tashiro et al., 2017). The animals used in this study were the most common models for PF—male and female rodents (rats and mice). Research shows that mice are most frequently used (Moore and Hogaboam, 2008), but rats were more common in this study. The average age of the animals was 9.5 weeks. PF is an aging-related disease, but most animal models are between 8 and 12 weeks old (Izbicki et al., 2002). Spontaneous fibrosis regression often occurs in young mice after a single bleomycin dose, but this does not happen in older mice. Multiple doses in young mice better mimic human pulmonary fibrosis (Tashiro et al., 2017). Regarding gender, the proportion of male animals in this study was significantly higher than that of females. The study shows that, regardless of age, male rodents exhibit a stronger response than females when bleomycin induces lung fibrosis (Redente et al., 2011). Based on these results and related reports, future research should focus on using older or young male mice with multiple doses to optimize models and better replicate PF features seen in humans (Stout-Delgado et al., 2016).

#### 4.3.2 Establishment of pulmonary fibrosis models

This meta-analysis did not restrict modeling methods before inclusion to better represent PF development’s various states and etiologies. Bleomycin was used for modeling in 80% of the included studies (n = 20). Bleomycin-induced pulmonary fibrosis modeling is the most common among currently applied experimentally induced pulmonary fibrosis animal models (Degryse and Lawson, 2011; Moore et al., 2013). The pathological features induced by bleomycin closely mimic those of end-stage pulmonary fibrosis in humans. They can dynamically demonstrate the progression of pulmonary fibrosis from acute injury to chronic fibrosis (Ayilya et al., 2023). Bleomycin is administered *via* multiple routes during modeling, with intratracheal administration being the most common and closest to the normal pulmonary fibrosis morphology in humans (Degryse and Lawson, 2011). Most of the studies in this review also used intratracheal administration for modeling. In addition, this study also included modeling using silica (n = 1), radiation (n = 2), delicate particulate matter (n = 1), and lipopolysaccharide (n = 1). Silica molding is similar to silicosis nodular fibrosis. It is related to occupational exposure (Arras et al., 2001). PF caused by this molding method often has a long course and stable condition (Barbarin et al., 2004). Radiation modeling can better demonstrate clinical pathological processes, and clinical experimental process parameters can be controlled, inducing more accurate results (Jin et al., 2020). The range of environmentally delicate particulate matter is greater than silicon dioxide, directly reflecting the impact of air pollution on the formation of pulmonary fibrosis, which has chronic and persistent characteristics (Li et al., 2024). The lipopolysaccharide-induced pulmonary fibrosis model has the core advantages of a precise inflammation-driven mechanism and well-defined immune regulatory targets, enabling it to simulate the progression of pulmonary fibrosis in the context of infection or inflammation (Nguyen et al., 2022). Bleomycin-induced pulmonary fibrosis models often present with acute lung tissue damage, and acute modeling results in high mortality rates in animal models (Gul et al., 2023). This study included five modeling methods, compensating for the shortcomings of a single modeling method.

#### 4.3.3 Administration routes for RES

This study included three administration methods: gavage (n = 17), intraperitoneal injection (n = 4), and oral administration (n = 2). Both gavage and oral administration simulate the human oral administration route. However, due to poor compliance with self-administration in experimental animals, such as factors affecting drug palatability and feeding rhythms, artificial gavage intervention is often used to ensure dose accuracy (Turner et al., 2011). Intestinal administration (including gavage and oral administration) requires absorption through the gastrointestinal tract before entering the systemic circulation. During this process, it undergoes metabolism by the gastrointestinal mucosa and the first-pass effect of the liver, resulting in a significant reduction in the bioavailability of RES (Doherty and Pang, 1997). Therefore, parenteral administration routes should be included when analyzing the impact of RES administration routes on PF efficacy. Intraperitoneal injection, as the most commonly used parenteral administration method, can effectively avoid the first-pass effect on drug activity (Lukas et al., 1971)and provide more comprehensive data support for the optimal RES administration strategy.

### 4.4 The effects and mechanisms of resveratrol on animal models of pulmonary fibrosis

#### 4.4.1 Core pathological processes

##### 4.4.1.1 Delaying the progression of fibrosis

The results of this meta-analysis, including pulmonary fibrosis histological scores, hydroxyproline (Hyp) content, and type I collagen (Col 1) content, can systematically assess fibrosis progression from a multidimensional perspective. First, pulmonary fibrosis histology scoring is considered the “gold standard” for diagnosing fibrosis (Hübner et al., 2008). It objectively reflects the morphological progression of fibrosis by directly observing histopathological features such as collagen deposition and alveolar structural damage. Secondly, as a collagen-specific amino acid, the tissue content of Hyp can not only be used to assess total collagen content quantitatively, but is also widely used in the efficacy evaluation system for anti-fibrotic drugs (Nakagome et al., 2006; Tang et al., 2022). In addition, abnormal deposition in Col 1 is not only a characteristic marker of fibrosis, but also constitutes the core pathological mechanism of lung tissue structural remodeling and functional decline by altering the mechanical properties of the extracellular matrix and triggering a cascade of pro-fibrotic signals (Shoda et al., 2007). The outcome measures of this meta-analysis showed that the RES experimental group had significantly lower fibrosis histology scores, hyp content, and Col 1 content in lung tissue compared to the control group, suggesting that RES not only inhibits key steps in collagen synthesis but also blocks the vicious cycle of fibrosis, ultimately achieving multiple protective effects such as slowing disease progression and maintaining alveolar gas exchange function.

##### 4.4.1.2 Reducing lung tissue inflammation

Alveolitis is a key initiating step in pulmonary fibrosis, characterized pathologically by inflammatory reactions in alveolar walls and interstitium. A persistent inflammatory microenvironment can activate fibroblasts, promote the release of pro-fibrotic factors, and induce excessive deposition of extracellular matrix, ultimately leading to irreversible structural damage to lung tissue and irreversible fibrotic scar formation (Song et al., 2016; Han et al., 2021). One of the outcome measures in this meta-analysis, the alveolitis score, showed that the alveolitis score in the RES experimental group was significantly lower than that in the control group, indicating that RES may effectively delay or even block the pathological transformation of inflammation to fibrosis, providing sufficient time for clinical intervention.

#### 4.4.2 Key molecular mechanisms

##### 4.4.2.1 Reducing the levels of pro-fibrotic factors

In the progression of pulmonary fibrosis, TGF-β acts as a classic pro-fibrotic factor, driving the pathological process through a dual mechanism: on one hand, it activates fibroblasts to differentiate into myofibroblasts, leading to excessive secretion of ECM proteins dominated by Col 1, thereby forming irreversible pulmonary tissue scarring (Peng et al., 2022); on the other hand, TGF-β can induce epithelial-mesenchymal transition (EMT) in alveolar epithelial cells, conferring migratory and invasive capabilities and a pro-fibrotic phenotype, thereby further exacerbating the formation of a fibrotic microenvironment (Inui et al., 2021). NF-κB, as a pro-fibrotic inflammatory regulatory factor, indirectly enhances the fibrotic process by mediating the cascade release of inflammatory factors such as IL-6 and TNF-α (Lawrence, 2009). These two key pathways do not act completely independently; there is a significant positive synergistic effect between TGF-β and NF-κB: the TGF-β/Smad signaling pathway enhances the transcriptional activity of NF-κB, while activated NF-κB promotes the synthesis and activation of TGF-β through a positive feedback loop (Wu W. et al., 2022), forming a continuously amplified pro-fibrotic signaling network. Data from this meta-analysis showed that TGF-β and NF-κB expression levels were significantly reduced in the lung tissue of animals in the RES experimental group. This suggests that it may effectively inhibit the cascade reaction of pro-fibrotic factors by simultaneously targeting these two key signaling nodes.

##### 4.4.2.2 Reducing the levels of inflammatory factors in lung tissue

TNF-α, IL-1β, and IL-6 are key factors mediating inflammatory responses, regulating the initiation, cascade amplification, and chronic progression of inflammation through multiple pathways (Tylutka et al., 2024). Regarding their mechanisms of action, the three agents exhibit synergistic effects. TNF-α, as a key regulator of early inflammation, is primarily secreted by activated macrophages and T lymphocytes. It activates the NF-κB signaling pathway, significantly upregulating the expression of other inflammatory factors and serving as the initial trigger point for the inflammatory cascade reaction (El-Tahan et al., 2016). IL-1β is produced through the activation of the NLRP3 inflammasome and forms a positive feedback regulatory loop with TNF-α, synergistically promoting the infiltration of neutrophils and monocytes (Bent et al., 2018). Animal experiments have confirmed that its specific antagonist can effectively delay fibrosis progression (Nan et al., 2021). IL-6 plays a dual role in pulmonary fibrosis: on the one hand, it acts as a typical inflammatory factor, exacerbating tissue inflammatory damage by regulating neutrophil recruitment and macrophage polarization (Rose-John, 2012); on the other hand, it acts as a direct driver of the fibrotic process, significantly enhancing pro-fibrotic effects by increasing the sensitivity of TGF-β receptor signaling (Wang Y. et al., 2022). The data from this meta-analysis showed that the expression levels of TNF-α, IL-1β, and IL-6 in the bronchoalveolar lavage fluid and plasma of animals in the RES experimental group were significantly reduced, suggesting that RES can improve the alveolar inflammatory microenvironment by inhibiting key inflammatory factor networks through multiple targets.

##### 4.4.2.3 Reducing oxidative stress factor levels

**FIGURE 13:**
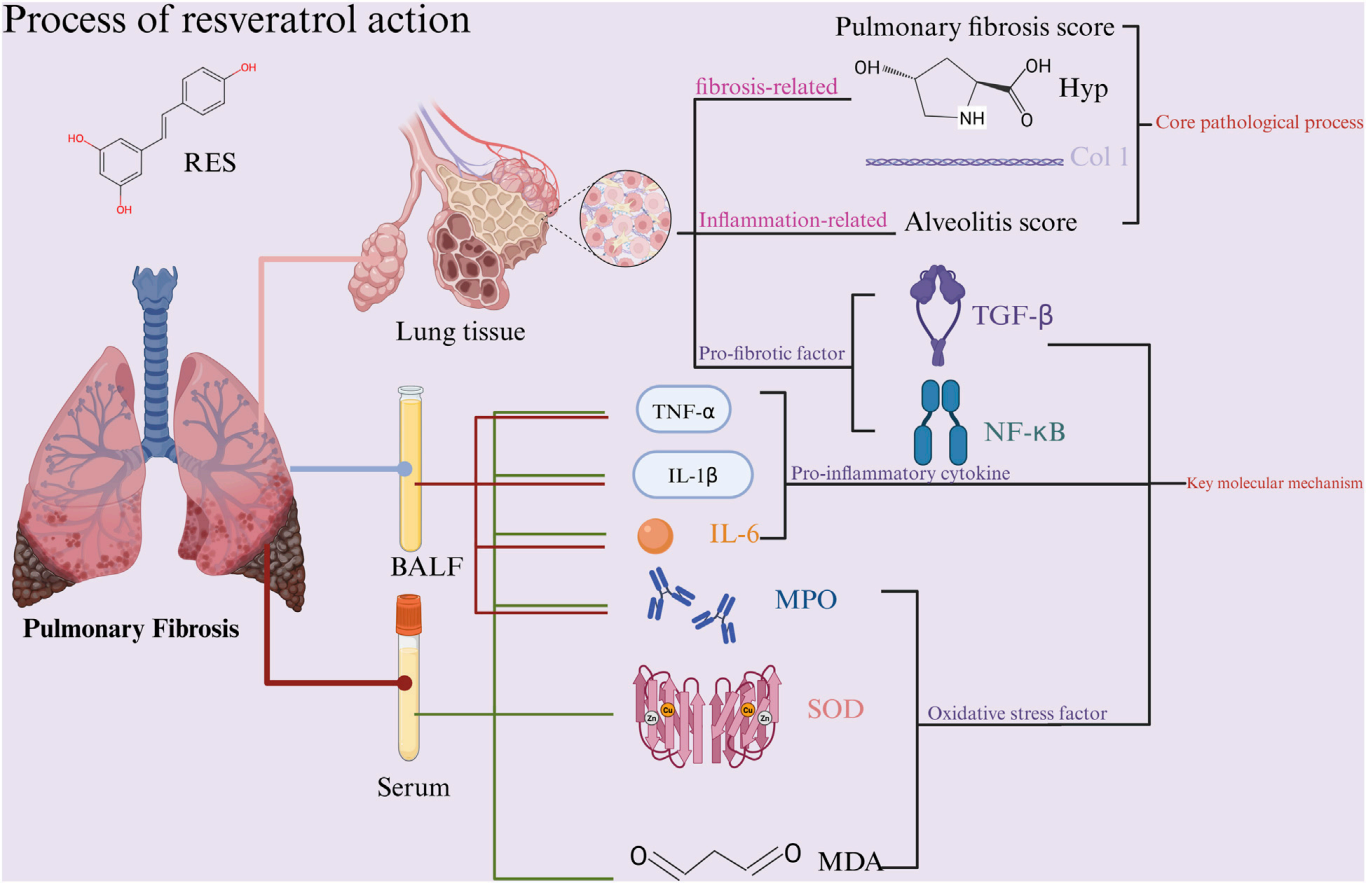
Schematic representation of the possible molecular mechanism of RES inhibition of PF. (Created using BioRender.com).

Oxidative stress, as one of the core mechanisms underlying the development and progression of PF, primarily manifests through the dynamic imbalance between reactive oxygen species (ROS) and the antioxidant system. This imbalanced state drives pulmonary tissue inflammatory cascades and fibrotic repair processes through dual pathways: direct cellular damage and indirect activation of signaling pathways (Luo et al., 2024). The factors related to oxidative stress in this outcome indicator are MDA, MPO, and SOD, which participate in the fibrotic pathological process from different dimensions. As the end product of lipid peroxidation, MDA damages alveolar epithelial cells, activates pro-fibrotic pathways such as TGF-β1, and accelerates the process of pulmonary fibrosis (Cordiano et al., 2023). As a neutrophil activation marker, MPO directly attacks the alveolar basement membrane structure by catalyzing strong oxidizing substances such as hypochlorous acid. It also promotes the release of inflammatory factors such as IL-6 and TNF-α, creating a persistent inflammatory microenvironment and forming a positive feedback loop of oxidative stress and inflammatory response (Lin et al., 2024). SOD, as a key enzyme in the endogenous antioxidant system, maintains redox homeostasis by specifically scavenging superoxide anions (O2-). Enhanced SOD activity helps protect mitochondrial function integrity and blocks ROS-induced alveolar epithelial cell apoptosis pathways (Younus, 2018). In this meta-analysis, the RES experimental group showed reduced levels of MDA and MPO in bronchoalveolar lavage fluid and plasma, while SOD activity was significantly increased. This suggests that RES may exert its effects through a multi-target regulatory mechanism, including inhibiting lipid peroxidation reactions, reducing neutrophil infiltration, and enhancing antioxidant enzyme activity, thereby synergistically improving pulmonary tissue oxidative stress and delaying the fibrotic process characterized by alveolar structural remodeling and abnormal extracellular matrix deposition (Figure 13).

### 4.5 Security

The results of this study indicate that even when the experimental group animals were administered RES at a dose of 100 mg/kg/day, no adverse reactions related to RES treatment for PF were observed in the study. However, the current data are insufficient to assess the safety profile of RES fully. Therefore, future studies should focus on the following aspects: first, researchers should establish standardized adverse reaction monitoring and reporting mechanisms to record any potential toxic reactions and avoid selective reporting objectively; second, the relationship between adverse reactions and factors such as dosage, route of administration, and treatment duration should be closely examined, combined with pharmacokinetic-related indicators for correlation analysis.

### 4.6 Limitation

The main challenge comes from the difference between animal models and real clinical cases. Current animal models, like the commonly used bleomycin-induced model, can’t fully mimic the complex features of human pulmonary fibrosis (Perel et al., 2007). A key issue is that rodents have much greater natural repair abilities than humans (Moeller et al., 2008), creating uncertainties when applying these models’ research results to human treatment. Also, most studies do not report the age of the animals well, but age is a crucial factor that affects how well the model works. This makes it challenging to ensure that all animals develop stable and reliable pulmonary fibrosis models, potentially affecting the accuracy of RES effectiveness evaluations due to the model’s reliability.

The considerable heterogeneity among studies and methodological limitations pose another significant constraint. There are notable differences between studies in detection methods and measurement units, such as histological scoring criteria and biochemical indicator detection techniques. Although standardized mean differences (SMD) were used to combine results, this method’s inherent limitations mean it cannot entirely eliminate systematic errors, and the indirect standardization process of the original data may also introduce calculation errors.

Even more concerning is the potential risk of publication bias. The analysis indicates that publication bias might exist for specific outcome measures. Specifically, studies showing positive results that demonstrate the efficacy of RES are more likely to be published. In contrast, studies with harmful or ineffective results may not be reported appropriately or published. This bias probably leads to an overestimation of the true efficacy of RES, thus impacting the objective assessment of its clinical value (Lin and Chu, 2018).

Finally, differences in resveratrol formulations and quality present another major obstacle. The RES used in the study varied significantly in source, purity, formulation, and administration route. Because RES itself has issues like low bioavailability and rapid metabolism (Ma et al., 2025), the pharmacokinetic properties of different formulations and methods vary widely. However, these crucial pharmaceutical factors were neither standardized nor adequately reported in the studies, making it difficult to compare results directly across studies and significantly impeding the determination of effective doses and optimal administration strategies for RES (Nyambuya et al., 2020). This undoubtedly poses a significant challenge for clinical translation.

In summary, despite the limitations mentioned earlier, resveratrol has shown promising potential for clinical use in treating pulmonary fibrosis (Berman et al., 2017). To advance the clinical application of RES, the following key issues need to be addressed: first, develop high-quality animal models that better resemble the pathophysiology of human pulmonary fibrosis to improve the models’ predictive accuracy; second, strictly standardize experimental reporting standards, adopt uniform assessment methods and guidelines to enhance result comparability across different studies; and finally, conduct detailed studies on the pharmacokinetics and pharmacodynamics of RES, optimize delivery systems to increase bioavailability, and establish a strong pharmaceutical foundation for future clinical trials.

### 4.7 Contribution to the 4Rs in ethnopharmacology

This systematic review and meta-analysis of preclinical studies on resveratrol for pulmonary fibrosis was conducted with a strong commitment to the ethical principles of the 4Rs framework (Reduce, Refine, Replace, Responsibility) in animal research.

#### 4.7.1 Reduce

By synthesizing data from 25 existing animal studies (totaling 985 animals), this review provides a comprehensive evidence base that can guide future research and potentially lessen the need for additional animal experiments. The detection of significant heterogeneity (e.g., in animal strains, drug sources, and modeling methods) underscores the importance of well-designed studies with adequate sample sizes. Our findings can help researchers optimize experimental designs, thereby reducing the number of animals required without compromising scientific validity.

#### 4.7.2 Refine

We used the SYRCLE risk of bias tool to evaluate the methodological quality of the included studies, identifying areas for improvement, such as randomization, blinding, and allocation concealment. Our review of modeling methods (e.g., bleomycin, radiation, silica) and their effects on outcomes provides insights into refining models to simulate human disease more accurately while minimizing animal distress. Additionally, our finding that male and older animals may be more suitable for pulmonary fibrosis research suggests that choosing these models could enhance animal welfare and improve data relevance.

#### 4.7.3 Replace

Although this review consolidates data from animal studies, it emphasizes the molecular mechanisms (e.g., TGF-β/Smad and NF-κB pathways) that could be further explored using alternative methods such as *in vitro* cell cultures or computational models. Additionally, the strong preclinical evidence presented here supports the transition of resveratrol into human clinical trials, which may ultimately reduce dependence on animal models.

#### 4.7.4 Responsibility

We highlight the ethical duty of researchers to report detailed methodologies, including drug sources, purity, animal characteristics, and adverse events, to ensure reproducibility and transparency. Our review also advocates for the use of standardized core outcome measures and safety monitoring in future studies, which are crucial for responsible research practice. By critically evaluating the limitations of current animal models and supporting improved study designs, we aim to promote responsible animal use in ethnopharmacological research.

Overall, this review improves the understanding of resveratrol’s therapeutic potential for pulmonary fibrosis and promotes the ethical development of animal research by adhering to the 4Rs principles.

## 5 Conclusion

This study is the first systematic review and meta-analysis of the effects and mechanisms of resveratrol (RES) in animal pulmonary fibrosis (PF) models. The results show that RES has significant anti-PF potential in animal models, mainly by slowing fibrosis progression, reducing inflammatory responses, and combating oxidative stress. However, because of limitations in the included studies, the interpretation and application of these findings should be cautiously approached. Still, the evidence from this research strongly supports RES as a highly promising anti-PF candidate drug, deserving further detailed investigation and progress toward clinical use. To address current limitations and advance RES’s clinical application, future preclinical studies must adopt strict standardization in design, including unified model validation standards, standardized specifications, detailed administration protocols for RES, and consistent detection methods and reporting metrics. This will help reduce heterogeneity and improve the comparability and reliability of results.

## Data Availability

The original contributions presented in the study are included in the article/Supplementary Material, further inquiries can be directed to the corresponding authors.

## References

[B1] Adams T. S. Schupp J. C. Poli S. Ayaub E. A. Neumark N. Ahangari F. (2020). Single-cell RNA-seq reveals ectopic and aberrant lung-resident cell populations in idiopathic pulmonary fibrosis. Sci. Adv. 6 (28), eaba1983. 10.1126/sciadv.aba1983 32832599 PMC7439502

[B2] Akgedik R. Akgedik S. Karamanli H. Uysal S. Bozkurt B. Ozol D. (2012). Effect of resveratrol on treatment of bleomycin-induced pulmonary fibrosis in rats. Inflammation 35 (5), 1732–1741. 10.1007/s10753-012-9491-0 22707284

[B3] Allen R. J. Porte J. Braybrooke R. Flores C. Fingerlin T. E. Oldham J. M. (2017). Genetic variants associated with susceptibility to idiopathic pulmonary fibrosis in people of European ancestry: a genome-wide association study. Lancet Respir. Med. 5 (11), 869–880. 10.1016/s2213-2600(17)30387-9 29066090 PMC5666208

[B4] Arras M. Huaux F. Vink A. Delos M. Coutelier J. P. Many M. C. (2001). Interleukin-9 reduces lung fibrosis and type 2 immune polarization induced by silica particles in a murine model. Am. J. Respir. Cell Mol. Biol. 24 (4), 368–375. 10.1165/ajrcmb.24.4.4249 11306428

[B5] Ayilya B. L. Balde A. Ramya M. Benjakul S. Kim S. K. Nazeer R. A. (2023). Insights on the mechanism of bleomycin to induce lung injury and associated *in vivo* models: a review. Int. Immunopharmacol. 121, 110493. 10.1016/j.intimp.2023.110493 37331299

[B6] Azmoonfar R. Amini P. Yahyapour R. Rezaeyan A. Tavassoli A. Motevaseli E. (2020). Mitigation of radiation-induced pneumonitis and lung fibrosis using alpha-lipoic acid and resveratrol. Anti-Inflammatory Anti-Allergy Agents Med. Chem. 19 (2), 149–157. 10.2174/1871523018666190319144020 30892165 PMC7509749

[B7] Bao L. Ye J. Liu N. Shao Y. Li W. Fan X. (2022). Resveratrol ameliorates fibrosis in rheumatoid arthritis-associated interstitial lung disease *via* the autophagy-lysosome pathway. Molecules 27 (23), 8475. 10.3390/molecules27238475 36500562 PMC9740423

[B8] Barbarin V. Arras M. Misson P. Delos M. McGarry B. Phan S. H. (2004). Characterization of the effect of interleukin-10 on silica-induced lung fibrosis in mice. Am. J. Respir. Cell Mol. Biol. 31 (1), 78–85. 10.1165/rcmb.2003-0299OC 14975940

[B9] Bent R. Moll L. Grabbe S. Bros M. (2018). Interleukin-1 Beta-A friend or foe in malignancies? Int. J. Mol. Sci. 19 (8), 2155. 10.3390/ijms19082155 30042333 PMC6121377

[B10] Berman A. Y. Motechin R. A. Wiesenfeld M. Y. Holz M. K. (2017). The therapeutic potential of resveratrol: a review of clinical trials. NPJ Precis. Oncol. 1, 35. 10.1038/s41698-017-0038-6 28989978 PMC5630227

[B11] Biondini D. Balestro E. Sverzellati N. Cocconcelli E. Bernardinello N. Ryerson C. J. (2020). Acute exacerbations of idiopathic pulmonary fibrosis (AE-IPF): an overview of current and future therapeutic strategies. Expert Rev. Respir. Med. 14 (4), 405–414. 10.1080/17476348.2020.1724096 31994940

[B12] Bringardner B. D. Baran C. P. Eubank T. D. Marsh C. B. (2008). The role of inflammation in the pathogenesis of idiopathic pulmonary fibrosis. Antioxid. Redox Signal 10 (2), 287–301. 10.1089/ars.2007.1897 17961066 PMC2737712

[B13] Cannito S. Novo E. di Bonzo L. V. Busletta C. Colombatto S. Parola M. (2010). Epithelial-mesenchymal transition: from molecular mechanisms, redox regulation to implications in human health and disease. Antioxid. Redox Signal 12 (12), 1383–1430. 10.1089/ars.2009.2737 19903090

[B14] Cao G. W. Mao W. D. (2008). Effect of resveratrol on bleomycln-induced pulmonary fibrosis in mice. J. Mod. Med. Health 24 (24), 3643–3645.

[B15] Chuliá-Peris L. Carreres-Rey C. Gabasa M. Alcaraz J. Carretero J. Pereda J. (2022). Matrix metalloproteinases and their inhibitors in pulmonary fibrosis: EMMPRIN/CD147 comes into play. Int. J. Mol. Sci. 23 (13), 6894. 10.3390/ijms23136894 35805895 PMC9267107

[B16] Cordiano R. Di Gioacchino M. Mangifesta R. Panzera C. Gangemi S. Minciullo P. L. (2023). Malondialdehyde as a potential oxidative stress marker for allergy-oriented diseases: an update. Molecules 28 (16), 5979. 10.3390/molecules28165979 37630231 PMC10457993

[B17] de Vries R. B. Wever K. E. Avey M. T. Stephens M. L. Sena E. S. Leenaars M. (2014). The usefulness of systematic reviews of animal experiments for the design of preclinical and clinical studies. Ilar J. 55 (3), 427–437. 10.1093/ilar/ilu043 25541545 PMC4276599

[B18] Degryse A. L. Lawson W. E. (2011). Progress toward improving animal models for idiopathic pulmonary fibrosis. Am. J. Med. Sci. 341 (6), 444–449. 10.1097/MAJ.0b013e31821aa000 21613932 PMC3103078

[B19] Ding S. Wang H. Wang M. Bai L. Yu P. Wu W. (2019). Resveratrol alleviates chronic “real-world” ambient particulate matter-induced lung inflammation and fibrosis by inhibiting NLRP3 inflammasome activation in mice. Ecotoxicol. Environ. Saf. 182, 109425. 10.1016/j.ecoenv.2019.109425 31295660

[B20] Dludla P. V. Silvestri S. Orlando P. Gabuza K. B. Mazibuko-Mbeje S. E. Nyambuya T. M. (2020). Exploring the comparative efficacy of metformin and resveratrol in the management of diabetes-associated complications: a systematic review of preclinical studies. Nutrients 12 (3), 739. 10.3390/nu12030739 32168855 PMC7146424

[B21] Doherty M. M. Pang K. S. (1997). First-pass effect: significance of the intestine for absorption and metabolism. Drug Chem. Toxicol. 20 (4), 329–344. 10.3109/01480549709003891 9433662

[B22] El-Tahan R. R. Ghoneim A. M. El-Mashad N. (2016). TNF-α gene polymorphisms and expression. Springerplus 5 (1), 1508. 10.1186/s40064-016-3197-y 27652081 PMC5014780

[B23] Galli J. A. Pandya A. Vega-Olivo M. Dass C. Zhao H. Criner G. J. (2017). Pirfenidone and nintedanib for pulmonary fibrosis in clinical practice: tolerability and adverse drug reactions. Respirology 22 (6), 1171–1178. 10.1111/resp.13024 28317233

[B24] Glass D. S. Grossfeld D. Renna H. A. Agarwala P. Spiegler P. DeLeon J. (2022). Idiopathic pulmonary fibrosis: current and future treatment. Clin. Respir. J. 16 (2), 84–96. 10.1111/crj.13466 35001525 PMC9060042

[B25] Gul A. Yang F. Xie C. Du W. Mohammadtursun N. Wang B. (2023). Pulmonary fibrosis model of mice induced by different administration methods of bleomycin. BMC Pulm. Med. 23 (1), 91. 10.1186/s12890-023-02349-z 36944966 PMC10029181

[B26] Gupta R. Morgan A. D. George P. M. Quint J. K. (2024). Incidence, prevalence and mortality of idiopathic pulmonary fibrosis in England from 2008 to 2018: a cohort study. THORAX 79 (7), 624–631. 10.1136/thorax-2023-220887 38688708

[B27] Han Y. Jiang M. He R. Lv X. Liao X. He Y. (2021). Mefunidone ameliorates bleomycin-induced pulmonary fibrosis in mice. Front. Pharmacol. 12, 713572. 10.3389/fphar.2021.713572 34630088 PMC8499630

[B28] He P. P. Wang Z. Y. Zhang P. Li W. S. Xiao X. Z. (2012). Comparison of intervention with different dosages of resveratrol on rats of pulmonary fibrosis induced by bleomycin. J. Clin. Pulm. Med. 17 (1), 3–5. 10.3969/j.issn.1009-6663.2012.01.002

[B29] Henderson N. C. Rieder F. Wynn T. A. (2020). Fibrosis: from mechanisms to medicines. Nature 587 (7835), 555–566. 10.1038/s41586-020-2938-9 33239795 PMC8034822

[B30] Hooijmans C. R. Rovers M. M. de Vries R. B. Leenaars M. Ritskes-Hoitinga M. Langendam M. W. (2014). SYRCLE's risk of bias tool for animal studies. BMC Med. Res. Methodol. 14, 43. 10.1186/1471-2288-14-43 24667063 PMC4230647

[B31] Huang S. Y. Cui H. S. Lyu M. S. Huang G. R. Hou D. Yu M. X. (2022). Efficacy of traditional Chinese medicine injections for treating idiopathic pulmonary fibrosis: a systematic review and network meta-analysis. PLoS One 17 (7), e0272047. 10.1371/journal.pone.0272047 35881610 PMC9321402

[B32] Hübner R. H. Gitter W. El Mokhtari N. E. Mathiak M. Both M. Bolte H. (2008). Standardized quantification of pulmonary fibrosis in histological samples. Biotechniques 44 (4), 507–517. 10.2144/000112729 18476815

[B33] Hutchinson J. Fogarty A. Hubbard R. McKeever T. (2015). Global incidence and mortality of idiopathic pulmonary fibrosis: a systematic review. Eur. Respir. J. 46 (3), 795–806. 10.1183/09031936.00185114 25976683

[B35] Impellizzeri D. Talero E. Siracusa R. Alcaide A. Cordaro M. Maria Zubelia J. (2015). Protective effect of polyphenols in an inflammatory process associated with experimental pulmonary fibrosis in mice. Br. J. Nutr. 114 (6), 853–865. 10.1017/S0007114515002597 26334388

[B36] Inui N. Sakai S. Kitagawa M. (2021). Molecular pathogenesis of pulmonary fibrosis, with focus on pathways related to TGF-β and the ubiquitin-proteasome pathway. Int. J. Mol. Sci. 22 (11), 6107. 10.3390/ijms22116107 34198949 PMC8201174

[B38] Ismail K. (2018). Resveratrol attenuates bleomycin-induced genotoxicity, pulmonary fibrosis and DNA damage in Balb/C mice with ehrlich ascites carcinoma. Bezmialem Sci. 6 (4), 262–271. 10.14235/bs.2018.2105

[B39] Izbicki G. Segel M. J. Christensen T. G. Conner M. W. Breuer R. (2002). Time course of bleomycin-induced lung fibrosis. Int. J. Exp. Pathol. 83 (3), 111–119. 10.1046/j.1365-2613.2002.00220.x 12383190 PMC2517673

[B40] Jannini-Sá Y. A. P. Creyns B. Hogaboam C. M. Parks W. C. Hohmann M. S. (2024). Macrophages in lung repair and fibrosis. Results Probl. Cell Differ. 74, 257–290. 10.1007/978-3-031-65944-7_10 39406909

[B41] Jiang Y. F. Wang H. Y. Li Y. Pan X. Y. Wang Y. Y. He L. N. (2024). Study on the protective mechanism of resveratrol against bleomycin in⁃duced pulmonary interstitial fibrosis in mice. Syst. Med. 9 (20), 5–28. 10.19368/j.cnki.2096-1782.2024.20.005

[B42] Jin S. Li S. Y. Chen F. N. Wang X. L. (2016). Single or combining use of salvianolic acid B,Glycyrrhetinic acid and resveratrol on pulmonary fibrosis mice induced by bleomycin. Chin. Archives Traditional Chin. Med. 34 (5), 1095–1098. 10.13193/j.issn.1673-7717.2016.05.020

[B43] Jin H. Yoo Y. Kim Y. Kim Y. Cho J. Lee Y. S. (2020). Radiation-induced lung fibrosis: preclinical animal models and therapeutic strategies. Cancers (Basel) 12 (6), 1561. 10.3390/cancers12061561 32545674 PMC7352529

[B44] Jo H. E. Glaspole I. Grainge C. Goh N. Hopkins P. M. Moodley Y. (2017). Baseline characteristics of idiopathic pulmonary fibrosis: analysis from the Australian idiopathic pulmonary fibrosis registry. Eur. Respir. J. 49 (2), 1601592. 10.1183/13993003.01592-2016 28232409

[B45] Karampitsakos T. Juan-Guardela B. M. Tzouvelekis A. Herazo-Maya J. D. (2023). Precision medicine advances in idiopathic pulmonary fibrosis. EBioMedicine 95, 104766. 10.1016/j.ebiom.2023.104766 37625268 PMC10469771

[B46] Karimi-Shah B. A. Chowdhury B. A. (2015). Forced vital capacity in idiopathic pulmonary fibrosis--FDA review of pirfenidone and nintedanib. N. Engl. J. Med. 372 (13), 1189–1191. 10.1056/NEJMp1500526 25806913

[B47] Kou M. Jiao Y. Li Z. Wei B. Li Y. Cai Y. (2024). Real-world safety and effectiveness of pirfenidone and nintedanib in the treatment of idiopathic pulmonary fibrosis: a systematic review and meta-analysis. Eur. J. Clin. Pharmacol. 80 (10), 1445–1460. 10.1007/s00228-024-03720-7 38963453

[B48] Koudstaal T. Funke-Chambour M. Kreuter M. Molyneaux P. L. Wijsenbeek M. S. (2023). Pulmonary fibrosis: from pathogenesis to clinical decision-making. Trends Mol. Med. 29 (12), 1076–1087. 10.1016/j.molmed.2023.08.010 37716906

[B49] Koushki M. Lakzaei M. Khodabandehloo H. Hosseini H. Meshkani R. Panahi G. (2020). Therapeutic effect of resveratrol supplementation on oxidative stress: a systematic review and meta-analysis of randomised controlled trials. Postgrad. Med. J. 96 (1134), 197–205. 10.1136/postgradmedj-2019-136415 31628212

[B50] Lawrence T. (2009). The nuclear factor NF-kappaB pathway in inflammation. Cold Spring Harb. Perspect. Biol. 1 (6), a001651. 10.1101/cshperspect.a001651 20457564 PMC2882124

[B51] Lebel M. Cliche D. O. Charbonneau M. Adam D. Brochiero E. Dubois C. M. (2022). Invadosome formation by lung fibroblasts in idiopathic pulmonary fibrosis. Int. J. Mol. Sci. 24 (1), 499. 10.3390/ijms24010499 36613948 PMC9820272

[B116] Li L. H. Lu B. Wu H. K. Zhang H. Yao F. F. (2016). Resveratrol inactivates sonic hedgehog signaling and alleviates lung fibrosis in rats. Chin. J. Mod. Med. 26 (1), 35–40.

[B52] Li W. S. Zhang P. He P. P. Xiao X. Z. (2012). Reverse of bleomycin-induced pulmonary fibrosis of rats by the effects of resveratrol. Clin. Med. Eng. 19 (2), 185–187. 10.3969/j.issn.1674-4659.2012.02.0185

[B53] Li Y. C. Jiang X. Fu F. Y. Li J. S. Wang X. F. (2015). Effects of resveratrol compined with irbesartan on pulmonary fibrosis and TGF-β1,NF-κB in rats. J. Clin. Exp. Med. (2), 83–86. 10.3969/j.issn.1671-4695.2015.02.002

[B54] Li J. Zeng G. Zhang Z. Wang Y. Shao M. Li C. (2024). Urban airborne PM(2.5) induces pulmonary fibrosis through triggering glycolysis and subsequent modification of histone lactylation in macrophages. Ecotoxicol. Environ. Saf. 273, 116162. 10.1016/j.ecoenv.2024.116162 38458067

[B55] Lin L. Chu H. (2018). Quantifying publication bias in meta-analysis. Biometrics 74 (3), 785–794. 10.1111/biom.12817 29141096 PMC5953768

[B56] Lin W. Chen H. Chen X. Guo C. (2024). The roles of neutrophil-derived myeloperoxidase (MPO) in diseases: the new progress. Antioxidants (Basel) 13 (1), 132. 10.3390/antiox13010132 38275657 PMC10812636

[B57] Liu L. J. Yu X. H. Zhang p. (2013). Resveratrol inhibits pulmonary fibrosis through TGF-β1/ADAMTS-1 signaling pathway. Chin. Pharmacol. Bull. 29 (3), 425–431. 10.3969/j.issn.1001-1978.2013.03.028

[B58] Liu Y. M. Nepali K. Liou J. P. (2017). Idiopathic pulmonary fibrosis: current status, recent progress, and emerging targets. J. Med. Chem. 60 (2), 527–553. 10.1021/acs.jmedchem.6b00935 28122457

[B59] Liu Y. L. Chen B. Y. Nie J. Zhao G. H. Zhuo J. Y. Yuan J. (2020). Polydatin prevents bleomycin-induced pulmonary fibrosis by inhibiting the TGF-β/Smad/ERK signaling pathway. Exp. Ther. Med. 20 (5), 62. 10.3892/etm.2020.9190 32952652 PMC7485305

[B60] Lukas G. Brindle S. D. Greengard P. (1971). The route of absorption of intraperitoneally administered compounds. J. Pharmacol. Exp. Ther. 178 (3), 562–566. 10.1016/s0022-3565(25)28988-1 5571904

[B61] Luo J. Li P. Dong M. Zhang Y. Lu S. Chen M. (2024). SLC15A3 plays a crucial role in pulmonary fibrosis by regulating macrophage oxidative stress. Cell Death Differ. 31 (4), 417–430. 10.1038/s41418-024-01266-w 38374230 PMC11043330

[B62] Ma M. Chu Z. Quan H. Li H. Zhou Y. Han Y. (2025). Natural products for anti-fibrotic therapy in idiopathic pulmonary fibrosis: marine and terrestrial insights. Front. Pharmacol. 16, 1524654. 10.3389/fphar.2025.1524654 40438605 PMC12116445

[B63] Mei Q. Liu Z. Zuo H. Yang Z. Qu J. (2021). Idiopathic pulmonary fibrosis: an update on pathogenesis. Front. Pharmacol. 12, 797292. 10.3389/fphar.2021.797292 35126134 PMC8807692

[B64] Meng T. Xiao D. Muhammed A. Deng J. Chen L. He J. (2021). Anti-inflammatory action and mechanisms of resveratrol. Molecules 26 (1), 229. 10.3390/molecules26010229 33466247 PMC7796143

[B65] Moeller A. Ask K. Warburton D. Gauldie J. Kolb M. (2008). The bleomycin animal model: a useful tool to investigate treatment options for idiopathic pulmonary fibrosis? Int. J. Biochem. Cell Biol. 40 (3), 362–382. 10.1016/j.biocel.2007.08.011 17936056 PMC2323681

[B66] Moore B. B. Hogaboam C. M. (2008). Murine models of pulmonary fibrosis. Am. J. Physiol. Lung Cell Mol. Physiol. 294 (2), L152–L160. 10.1152/ajplung.00313.2007 17993587

[B67] Moore B. B. Lawson W. E. Oury T. D. Sisson T. H. Raghavendran K. Hogaboam C. M. (2013). Animal models of fibrotic lung disease. Am. J. Respir. Cell Mol. Biol. 49 (2), 167–179. 10.1165/rcmb.2013-0094TR 23526222 PMC3824038

[B68] Mora A. L. Rojas M. Pardo A. Selman M. (2017). Emerging therapies for idiopathic pulmonary fibrosis, a progressive age-related disease. Nat. Rev. Drug Discov. 16 (11), 755–772. 10.1038/nrd.2017.170 28983101

[B69] Mortimer K. M. Bartels D. B. Hartmann N. Capapey J. Yang J. Gately R. (2020). Characterizing health outcomes in idiopathic pulmonary fibrosis using US health claims data. Respiration 99 (2), 108–118. 10.1159/000504630 31982886 PMC7949201

[B70] Nakagome K. Dohi M. Okunishi K. Tanaka R. Miyazaki J. Yamamoto K. (2006). *In vivo* IL-10 gene delivery attenuates bleomycin induced pulmonary fibrosis by inhibiting the production and activation of TGF-beta in the lung. Thorax 61 (10), 886–894. 10.1136/thx.2005.056317 16809410 PMC2104751

[B71] Nan D. Abraira-Meriel C. de la Roz-Fernández S. Maestre-Orozco T. Hernandez J. L. Fernandez-Ayala M. (2021). Delayed use of the recombinant human IL-1 receptor antagonist anakinra in five COVID-19 patients with pulmonary fibrosis and persistent hypoxaemia: a preliminary report. Eur. J. Case Rep. Intern Med. 8 (10), 002821. 10.12890/2021_002821 34790623 PMC8592658

[B72] Nguyen N. Xu S. Lam T. Y. W. Liao W. Wong W. S. F. Ge R. (2022). ISM1 suppresses LPS-induced acute lung injury and post-injury lung fibrosis in mice. Mol. Med. 28 (1), 72. 10.1186/s10020-022-00500-w 35752760 PMC9233842

[B73] Nyambuya T. M. Nkambule B. B. Mazibuko-Mbeje S. E. Mxinwa V. Mokgalaboni K. Orlando P. (2020). A meta-analysis of the impact of resveratrol supplementation on markers of renal function and blood pressure in type 2 diabetic patients on hypoglycemic therapy. Molecules 25 (23), 5645. 10.3390/molecules25235645 33266114 PMC7730696

[B74] Otoupalova E. Smith S. Cheng G. Thannickal V. J. (2020). Oxidative stress in pulmonary fibrosis. Compr. Physiol. 10 (2), 509–547. 10.1002/cphy.c190017 32163196

[B75] Page M. J. McKenzie J. E. Bossuyt P. M. Boutron I. Hoffmann T. C. Mulrow C. D. (2021). The PRISMA 2020 statement: an updated guideline for reporting systematic reviews. Bmj 372, n71. 10.1136/bmj.n71 33782057 PMC8005924

[B76] Peng D. Fu M. Wang M. Wei Y. Wei X. (2022). Targeting TGF-β signal transduction for fibrosis and cancer therapy. Mol. Cancer 21 (1), 104. 10.1186/s12943-022-01569-x 35461253 PMC9033932

[B77] Perel P. Roberts I. Sena E. Wheble P. Briscoe C. Sandercock P. (2007). Comparison of treatment effects between animal experiments and clinical trials: systematic review. Bmj 334, 197. 10.1136/bmj.39048.407928.BE 17175568 PMC1781970

[B78] Podolanczuk A. J. Thomson C. C. Remy-Jardin M. Richeldi L. Martinez F. J. Kolb M. (2023). Idiopathic pulmonary fibrosis: state of the art for 2023. Eur. Respir. J. 61 (4), 2200957. 10.1183/13993003.00957-2022 36702498

[B79] Quan Y. F. Tang Q. Xing C. Liu F. Li M. Li C. (2023). Effects of resveratrol on pulmonary fibrosis and chitinase-3-like protein 1 expression in silicosis-rats. Chin. J. Industrial Med. 36 (4), 335–封303. 10.13631/j.cnki.zggyyx.2023.04.004

[B80] Raghu G. Chen S. Y. Hou Q. Yeh W. S. Collard H. R. (2016). Incidence and prevalence of idiopathic pulmonary fibrosis in US adults 18-64 years old. Eur. Respir. J. 48 (1), 179–186. 10.1183/13993003.01653-2015 27126689

[B81] Raghu G. Remy-Jardin M. Myers J. L. Richeldi L. Ryerson C. J. Lederer D. J. (2018). Diagnosis of idiopathic pulmonary fibrosis. An official ATS/ERS/JRS/ALAT clinical practice guideline. Am. J. Respir. Crit. Care Med. 198 (5), e44–e68. 10.1164/rccm.201807-1255ST 30168753

[B82] Raghu G. Remy-Jardin M. Richeldi L. Thomson C. C. Inoue Y. Johkoh T. (2022). Idiopathic pulmonary fibrosis (an update) and progressive pulmonary fibrosis in adults: an official ATS/ERS/JRS/ALAT clinical practice guideline. Am. J. Respir. Crit. Care Med. 205 (9), e18–e47. 10.1164/rccm.202202-0399ST 35486072 PMC9851481

[B83] Ramli I. Cheriet T. Posadino A. M. Giordo R. Zayed H. Eid A. H. (2023). Potential therapeutic targets of resveratrol in the prevention and treatment of pulmonary fibrosis. Front. Biosci. 28 (9), 198. 10.31083/j.fbl2809198 37796708

[B84] Redente E. F. Jacobsen K. M. Solomon J. J. Lara A. R. Faubel S. Keith R. C. (2011). Age and sex dimorphisms contribute to the severity of bleomycin-induced lung injury and fibrosis. Am. J. Physiol. Lung Cell Mol. Physiol. 301 (4), L510–L518. 10.1152/ajplung.00122.2011 21743030 PMC3191751

[B85] Rose-John S. (2012). IL-6 trans-signaling *via* the soluble IL-6 receptor: importance for the pro-inflammatory activities of IL-6. Int. J. Biol. Sci. 8 (9), 1237–1247. 10.7150/ijbs.4989 23136552 PMC3491447

[B86] Selman M. Pardo A. (2020). The leading role of epithelial cells in the pathogenesis of idiopathic pulmonary fibrosis. Cell Signal 66, 109482. 10.1016/j.cellsig.2019.109482 31760172

[B87] Şener G. Topaloǧlu N. Özer Şehirli A. Ercan F. Gedik N. (2007). Resveratrol alleviates bleomycin-induced lung injury in rats. Pulm. Pharmacol. Ther. 20 (6), 642–649. 10.1016/j.pupt.2006.07.003 17035056

[B88] Shoda H. Yokoyama A. Nishino R. Nakashima T. Ishikawa N. Haruta Y. (2007). Overproduction of collagen and diminished SOCS1 expression are causally linked in fibroblasts from idiopathic pulmonary fibrosis. Biochem. Biophys. Res. Commun. 353 (4), 1004–1010. 10.1016/j.bbrc.2006.12.128 17198680

[B89] Somogyi V. Chaudhuri N. Torrisi S. E. Kahn N. Müller V. Kreuter M. (2019). The therapy of idiopathic pulmonary fibrosis: what is next? Eur. Respir. Rev. 28 (153), 190021. 10.1183/16000617.0021-2019 31484664 PMC9488691

[B90] Song C. He L. Zhang J. Ma H. Yuan X. Hu G. (2016). Fluorofenidone attenuates pulmonary inflammation and fibrosis *via* inhibiting the activation of NALP3 inflammasome and IL-1β/IL-1R1/MyD88/NF-κB pathway. J. Cell Mol. Med. 20 (11), 2064–2077. 10.1111/jcmm.12898 27306439 PMC5082399

[B91] Stout-Delgado H. W. Cho S. J. Chu S. G. Mitzel D. N. Villalba J. El-Chemaly S. (2016). Age-dependent susceptibility to pulmonary fibrosis is associated with NLRP3 inflammasome activation. Am. J. Respir. Cell Mol. Biol. 55 (2), 252–263. 10.1165/rcmb.2015-0222OC 26933834 PMC4979364

[B92] Tang Q. Xing C. Li M. Jia Q. Bo C. Zhang Z. (2022). Pirfenidone ameliorates pulmonary inflammation and fibrosis in a rat silicosis model by inhibiting macrophage polarization and JAK2/STAT3 signaling pathways. Ecotoxicol. Environ. Saf. 244, 114066. 10.1016/j.ecoenv.2022.114066 36108436

[B93] Tashiro J. Rubio G. A. Limper A. H. Williams K. Elliot S. J. Ninou I. (2017). Exploring animal models that resemble idiopathic pulmonary fibrosis. Front. Med. (Lausanne) 4, 118. 10.3389/fmed.2017.00118 28804709 PMC5532376

[B95] Tian B. Liu J. (2020). Resveratrol: a review of plant sources, synthesis, stability, modification and food application. J. Sci. Food Agric. 100 (4), 1392–1404. 10.1002/jsfa.10152 31756276

[B96] Turner P. V. Brabb T. Pekow C. Vasbinder M. A. (2011). Administration of substances to laboratory animals: routes of administration and factors to consider. J. Am. Assoc. Lab. Anim. Sci. 50 (5), 600–613. 22330705 PMC3189662

[B97] Tylutka A. Walas Ł. Zembron-Lacny A. (2024). Level of IL-6, TNF, and IL-1β and age-related diseases: a systematic review and meta-analysis. Front. Immunol. 15, 1330386. 10.3389/fimmu.2024.1330386 38495887 PMC10943692

[B98] Wang J. He F. Chen L. Li Q. Jin S. Zheng H. (2018). Resveratrol inhibits pulmonary fibrosis by regulating miR-21 through MAPK/AP-1 pathways. Biomed. Pharmacother. 105, 37–44. 10.1016/j.biopha.2018.05.104 29843043

[B99] Wang Z. Y. Zhang P. He P. P. Yu X. H. (2011a). Effects of resveratrol on bleomycin-induced pulmonary fibrosis of the rat and expression of NF-κB. J. Clin. Pulm. Med. 16 (9), 1315–1317. 10.3969/j.issn.1009-6663.2011.09.002

[B100] Wang Z. Y. Zhang P. He P. P. Yu X. H. (2011b). Expression of nuclear factor-κB and intervention of resveratrol in rats with pulmonary fibrosis. Int. J. Respir. 31 (12), 907–912. 10.3760/cma.j.issn.1673-436X.2011.012.006

[B101] Wang Z. Li X. Chen H. Han L. Ji X. Wang Q. (2021). Resveratrol alleviates bleomycin-induced pulmonary fibrosis *via* suppressing HIF-1α and NF-κB expression. Aging 13 (3), 4605–4616. 10.18632/aging.202420 33495418 PMC7906133

[B102] Wang L. Shao M. Jiang W. Huang Y. (2022a). Resveratrol alleviates bleomycin-induced pulmonary fibrosis by inhibiting epithelial-mesenchymal transition and down-regulating TLR4/NF-κB and TGF-β1/smad3 signalling pathways in rats. Tissue Cell 79, 101953. 10.1016/j.tice.2022.101953 36228366

[B103] Wang Y. Sang X. Shao R. Qin H. Chen X. Xue Z. (2022b). Xuanfei Baidu Decoction protects against macrophages induced inflammation and pulmonary fibrosis *via* inhibiting IL-6/STAT3 signaling pathway. J. Ethnopharmacol. 283, 114701. 10.1016/j.jep.2021.114701 34606948 PMC9715986

[B104] Wijsenbeek M. Cottin V. (2020). Spectrum of fibrotic lung diseases. N. Engl. J. Med. 383 (10), 958–968. 10.1056/NEJMra2005230 32877584

[B105] Willis B. C. Borok Z. (2007). TGF-beta-induced EMT: mechanisms and implications for fibrotic lung disease. Am. J. Physiol. Lung Cell Mol. Physiol. 293 (3), L525–L534. 10.1152/ajplung.00163.2007 17631612

[B106] Wu J. Huang H. Tu M. Yu H. Wei T. Huang X. (2022a). Acute toxicological study: EZY-1 with potent therapeutic effects of idiopathic pulmonary fibrosis and its mechanisms. J. Food Biochem. 46 (12), e14483. 10.1111/jfbc.14483 36226766

[B107] Wu W. Wang X. Yu X. Lan H. Y. (2022b). Smad3 signatures in renal inflammation and fibrosis. Int. J. Biol. Sci. 18 (7), 2795–2806. 10.7150/ijbs.71595 35541902 PMC9066101

[B108] Wu X. Xiao X. Chen X. Yang M. Hu Z. Shuai S. (2022c). Effectiveness and mechanism of metformin in animal models of pulmonary fibrosis: a preclinical systematic review and meta-analysis. Front. Pharmacol. 13, 948101. 10.3389/fphar.2022.948101 36147352 PMC9485720

[B109] Wu X. Xiao X. Su Y. Zhang Y. Li G. Wang F. (2025). Use quercetin for pulmonary fibrosis: a preclinical systematic review and meta-analysis. Inflammopharmacology 33 (4), 1879–1897. 10.1007/s10787-025-01678-1 40038212

[B110] Xu B. Wang T. Guo W. Liu D. P. (2017). Protective effect of resveratrol combined with Irbesartan on Bleomycin—induced pulmonary fibrosis in rats and mechanism study. Med Pharm J Chin PLA 29 (7), 1–4. 10.3969/j.issn.2095-140X.2017.07.001

[B111] Yahyapour R. Amini P. Saffar H. Motevaseli E. Farhood B. Pooladvand V. (2019). Protective effect of metformin, resveratrol and alpha-lipoic acid on Radiation- induced pneumonitis and fibrosis: a histopathological study. Curr. Drug Res. Rev. 11 (2), 111–117. 10.2174/2589977511666191018180758 31875783

[B112] Younus H. (2018). Therapeutic potentials of superoxide dismutase. Int. J. Health Sci. (Qassim) 12 (3), 88–93. 29896077 PMC5969776

[B113] Zhang Y. Chen L. Y. Liang B. (2011). Effects of polydatin on bleomycin-induced pulmonary fibrosis in rats. China J. Chin. Materia Medica 36 (24), 3528–3534. 10.4268/cjcmm20112431 22368872

[B114] Zhang Y. Q. Liu Y. J. Mao Y. F. Dong W. W. Zhu X. Y. Jiang L. (2015). Resveratrol ameliorates lipopolysaccharide-induced epithelial mesenchymal transition and pulmonary fibrosis through suppression of oxidative stress and transforming growth factor-β1 signaling. Clin. Nutr. 34 (4), 752–760. 10.1016/j.clnu.2014.08.014 25234611

[B115] Zordoky B. N. Robertson I. M. Dyck J. R. (2015). Preclinical and clinical evidence for the role of resveratrol in the treatment of cardiovascular diseases. Biochim. Biophys. Acta 1852 (6), 1155–1177. 10.1016/j.bbadis.2014.10.016 25451966

