# Supplementary material for: Protective effects and mechanism of resveratrol in animal models of pulmonary fibrosis: a preclinical systematic review and meta-analysis
Source: Front Pharmacol. 2025 Sep 29;16:1666698. doi: 10.3389/fphar.2025.1666698 (PMC12515909; doi:10.3389/fphar.2025.1666698)

Supplementary Material

# Supplementary Tables

## Supplementary Table S1 A detailed search strategy for each database

| PubMed   \| Search number \| Query \| Results \| \| --- \| --- \| --- \| \| 30 \| #29 AND #26 AND #13 \| 103 \| \| 29 \| (Resveratrol[MeSH Terms]) OR ((((((((((((((((Resveratrol[Title/Abstract]) OR (3,4',5-Stilbenetriol[Title/Abstract])) OR (3,5,4'-Trihydroxystilbene[Title/Abstract])) OR (3,4',5-Trihydroxystilbene[Title/Abstract])) OR (trans-Resveratrol[Title/Abstract])) OR (trans Resveratrol[Title/Abstract])) OR (Resveratrol-3-sulfate[Title/Abstract])) OR (Resveratrol 3 sulfate[Title/Abstract])) OR (SRT 501[Title/Abstract])) OR (SRT-501[Title/Abstract])) OR (SRT501[Title/Abstract])) OR (cis-Resveratrol[Title/Abstract])) OR (cis Resveratrol[Title/Abstract])) OR (Resveratrol, (Z)-[Title/Abstract])) OR (trans-Resveratrol-3-O-sulfate[Title/Abstract])) OR (trans Resveratrol 3 O sulfate[Title/Abstract])) \| 19,313 \| \| 28 \| (((((((((((((((Resveratrol[Title/Abstract]) OR (3,4',5-Stilbenetriol[Title/Abstract])) OR (3,5,4'-Trihydroxystilbene[Title/Abstract])) OR (3,4',5-Trihydroxystilbene[Title/Abstract])) OR (trans-Resveratrol[Title/Abstract])) OR (trans Resveratrol[Title/Abstract])) OR (Resveratrol-3-sulfate[Title/Abstract])) OR (Resveratrol 3 sulfate[Title/Abstract])) OR (SRT 501[Title/Abstract])) OR (SRT-501[Title/Abstract])) OR (SRT501[Title/Abstract])) OR (cis-Resveratrol[Title/Abstract])) OR (cis Resveratrol[Title/Abstract])) OR (Resveratrol, (Z)-[Title/Abstract])) OR (trans-Resveratrol-3-O-sulfate[Title/Abstract])) OR (trans Resveratrol 3 O sulfate[Title/Abstract]) \| 18,927 \| \| 27 \| Resveratrol[MeSH Terms] \| 11,825 \| \| 26 \| #16 OR #19 OR #22 OR #25 \| 28,060,692 \| \| 25 \| (Animal Experimentation[MeSH Terms]) OR ((((((((((((Animal Experimentation[Title/Abstract]) OR (Experimentation, Animal[Title/Abstract])) OR (Animal Experimental Use[Title/Abstract])) OR (Animal Experimental Uses[Title/Abstract])) OR (Experimental Use, Animal[Title/Abstract])) OR (Experimental Uses, Animal[Title/Abstract])) OR (Animal Research[Title/Abstract])) OR (Research, Animal[Title/Abstract])) OR (Animal Experiments[Title/Abstract])) OR (Animal Experiment[Title/Abstract])) OR (Experiment, Animal[Title/Abstract])) OR (Experiments, Animal[Title/Abstract])) \| 40,223 \| \| 24 \| (((((((((((Animal Experimentation[Title/Abstract]) OR (Experimentation, Animal[Title/Abstract])) OR (Animal Experimental Use[Title/Abstract])) OR (Animal Experimental Uses[Title/Abstract])) OR (Experimental Use, Animal[Title/Abstract])) OR (Experimental Uses, Animal[Title/Abstract])) OR (Animal Research[Title/Abstract])) OR (Research, Animal[Title/Abstract])) OR (Animal Experiments[Title/Abstract])) OR (Animal Experiment[Title/Abstract])) OR (Experiment, Animal[Title/Abstract])) OR (Experiments, Animal[Title/Abstract]) \| 32,076 \| \| 23 \| Animal Experimentation[MeSH Terms] \| 10,662 \| \| 22 \| (Animals, Laboratory[MeSH Terms]) OR ((((Animals, Laboratory[Title/Abstract]) OR (Animal, Laboratory[Title/Abstract])) OR (Laboratory Animal[Title/Abstract])) OR (Laboratory Animals[Title/Abstract])) \| 1,004,178 \| \| 21 \| (((Animals, Laboratory[Title/Abstract]) OR (Animal, Laboratory[Title/Abstract])) OR (Laboratory Animal[Title/Abstract])) OR (Laboratory Animals[Title/Abstract]) \| 19,626 \| \| 20 \| Animals, Laboratory[MeSH Terms] \| 989,924 \| \| 19 \| (Models, Animal[MeSH Terms]) OR ((((((((((((((((Models, Animal[Title/Abstract]) OR (Animal Model[Title/Abstract])) OR (Animal Models[Title/Abstract])) OR (Model, Animal[Title/Abstract])) OR (Experimental Animal Models[Title/Abstract])) OR (Animal Model, Experimental[Title/Abstract])) OR (Animal Models, Experimental[Title/Abstract])) OR (Experimental Animal Model[Title/Abstract])) OR (Model, Experimental Animal[Title/Abstract])) OR (Models, Experimental Animal[Title/Abstract])) OR (Laboratory Animal Models[Title/Abstract])) OR (Animal Model, Laboratory[Title/Abstract])) OR (Animal Models, Laboratory[Title/Abstract])) OR (Laboratory Animal Model[Title/Abstract])) OR (Model, Laboratory Animal[Title/Abstract])) OR (Models, Laboratory Animal[Title/Abstract])) \| 830,529 \| \| 18 \| (((((((((((((((Models, Animal[Title/Abstract]) OR (Animal Model[Title/Abstract])) OR (Animal Models[Title/Abstract])) OR (Model, Animal[Title/Abstract])) OR (Experimental Animal Models[Title/Abstract])) OR (Animal Model, Experimental[Title/Abstract])) OR (Animal Models, Experimental[Title/Abstract])) OR (Experimental Animal Model[Title/Abstract])) OR (Model, Experimental Animal[Title/Abstract])) OR (Models, Experimental Animal[Title/Abstract])) OR (Laboratory Animal Models[Title/Abstract])) OR (Animal Model, Laboratory[Title/Abstract])) OR (Animal Models, Laboratory[Title/Abstract])) OR (Laboratory Animal Model[Title/Abstract])) OR (Model, Laboratory Animal[Title/Abstract])) OR (Models, Laboratory Animal[Title/Abstract]) \| 235,248 \| \| 17 \| Models, Animal[MeSH Terms] \| 675,443 \| \| 16 \| (Animals[MeSH Terms]) OR ((((Animals[Title/Abstract]) OR (Animal[Title/Abstract])) OR (Animalia[Title/Abstract])) OR (Metazoa[Title/Abstract])) \| 28,058,934 \| \| 15 \| (((Animals[Title/Abstract]) OR (Animal[Title/Abstract])) OR (Animalia[Title/Abstract])) OR (Metazoa[Title/Abstract]) \| 1,344,282 \| \| 14 \| Animals[MeSH Terms] \| 27,877,595 \| \| 13 \| #3 OR #6 OR #9 OR #12 \| 136,703 \| \| 12 \| (Respiratory Distress Syndrome[MeSH Terms]) OR (((((((Respiratory Distress Syndrome[Title/Abstract]) OR (Distress Syndrome, Respiratory[Title/Abstract])) OR (Distress Syndromes, Respiratory[Title/Abstract])) OR (Respiratory Distress Syndromes[Title/Abstract])) OR (Syndrome, Respiratory Distress[Title/Abstract])) OR (Shock Lung[Title/Abstract])) OR (Lung, Shock[Title/Abstract])) \| 62,334 \| \| 11 \| ((((((Respiratory Distress Syndrome[Title/Abstract]) OR (Distress Syndrome, Respiratory[Title/Abstract])) OR (Distress Syndromes, Respiratory[Title/Abstract])) OR (Respiratory Distress Syndromes[Title/Abstract])) OR (Syndrome, Respiratory Distress[Title/Abstract])) OR (Shock Lung[Title/Abstract])) OR (Lung, Shock[Title/Abstract]) \| 41,163 \| \| 10 \| Respiratory Distress Syndrome[MeSH Terms] \| 42,331 \| \| 9 \| (Acute Lung Injury[MeSH Terms]) OR ((((Acute Lung Injury[Title/Abstract]) OR (Acute Lung Injuries[Title/Abstract])) OR (Lung Injuries, Acute[Title/Abstract])) OR (Lung Injury, Acute[Title/Abstract])) \| 20,813 \| \| 8 \| (((Acute Lung Injury[Title/Abstract]) OR (Acute Lung Injuries[Title/Abstract])) OR (Lung Injuries, Acute[Title/Abstract])) OR (Lung Injury, Acute[Title/Abstract]) \| 19,099 \| \| 7 \| Acute Lung Injury[MeSH Terms] \| 9,761 \| \| 6 \| (Idiopathic Pulmonary Fibrosis[MeSH Terms]) OR (((((((((((((((Idiopathic Pulmonary Fibrosis[Title/Abstract]) OR (Idiopathic Pulmonary Fibroses[Title/Abstract])) OR (Pulmonary Fibroses, Idiopathic[Title/Abstract])) OR (Cryptogenic Fibrosing Alveolitis[Title/Abstract])) OR (Cryptogenic Fibrosing Alveolitides[Title/Abstract])) OR (Fibrosing Alveolitides, Cryptogenic[Title/Abstract])) OR (Pulmonary Fibrosis, Idiopathic[Title/Abstract])) OR (Fibrosing Alveolitis, Cryptogenic[Title/Abstract])) OR (Fibrocystic Pulmonary Dysplasia[Title/Abstract])) OR (Dysplasia, Fibrocystic Pulmonary[Title/Abstract])) OR (Fibrocystic Pulmonary Dysplasias[Title/Abstract])) OR (Pulmonary Dysplasia, Fibrocystic[Title/Abstract])) OR (Idiopathic Fibrosing Alveolitis, Chronic Form[Title/Abstract])) OR (Familial Idiopathic Pulmonary Fibrosis[Title/Abstract])) OR (Idiopathic Pulmonary Fibrosis, Familial[Title/Abstract])) \| 17,601 \| \| 5 \| ((((((((((((((Idiopathic Pulmonary Fibrosis[Title/Abstract]) OR (Idiopathic Pulmonary Fibroses[Title/Abstract])) OR (Pulmonary Fibroses, Idiopathic[Title/Abstract])) OR (Cryptogenic Fibrosing Alveolitis[Title/Abstract])) OR (Cryptogenic Fibrosing Alveolitides[Title/Abstract])) OR (Fibrosing Alveolitides, Cryptogenic[Title/Abstract])) OR (Pulmonary Fibrosis, Idiopathic[Title/Abstract])) OR (Fibrosing Alveolitis, Cryptogenic[Title/Abstract])) OR (Fibrocystic Pulmonary Dysplasia[Title/Abstract])) OR (Dysplasia, Fibrocystic Pulmonary[Title/Abstract])) OR (Fibrocystic Pulmonary Dysplasias[Title/Abstract])) OR (Pulmonary Dysplasia, Fibrocystic[Title/Abstract])) OR (Idiopathic Fibrosing Alveolitis, Chronic Form[Title/Abstract])) OR (Familial Idiopathic Pulmonary Fibrosis[Title/Abstract])) OR (Idiopathic Pulmonary Fibrosis, Familial[Title/Abstract]) \| 16,032 \| \| 4 \| Idiopathic Pulmonary Fibrosis[MeSH Terms] \| 9,171 \| \| 3 \| (Pulmonary Fibrosis[MeSH Terms]) OR (((((((((Pulmonary Fibrosis[Title/Abstract]) OR (Fibroses, Pulmonary[Title/Abstract])) OR (Fibrosis, Pulmonary[Title/Abstract])) OR (Pulmonary Fibroses[Title/Abstract])) OR (Alveolitis, Fibrosing[Title/Abstract])) OR (Alveolitides, Fibrosing[Title/Abstract])) OR (Fibrosing Alveolitides[Title/Abstract])) OR (Fibrosing Alveolitis[Title/Abstract])) OR (Idiopathic Diffuse Interstitial Pulmonary Fibrosis[Title/Abstract])) \| 62,309 \| \| 2 \| ((((((((Pulmonary Fibrosis[Title/Abstract]) OR (Fibroses, Pulmonary[Title/Abstract])) OR (Fibrosis, Pulmonary[Title/Abstract])) OR (Pulmonary Fibroses[Title/Abstract])) OR (Alveolitis, Fibrosing[Title/Abstract])) OR (Alveolitides, Fibrosing[Title/Abstract])) OR (Fibrosing Alveolitides[Title/Abstract])) OR (Fibrosing Alveolitis[Title/Abstract])) OR (Idiopathic Diffuse Interstitial Pulmonary Fibrosis[Title/Abstract]) \| 53,640 \| \| 1 \| Pulmonary Fibrosis[MeSH Terms] \| 30,332 \| |
| --- | --- | --- | --- | --- | --- | --- | --- | --- | --- | --- | --- | --- | --- | --- | --- | --- | --- | --- | --- | --- | --- | --- | --- | --- | --- | --- | --- | --- | --- | --- | --- | --- | --- | --- | --- | --- | --- | --- | --- | --- | --- | --- | --- | --- | --- | --- | --- | --- | --- | --- | --- | --- | --- | --- | --- | --- | --- | --- | --- | --- | --- | --- | --- | --- | --- | --- | --- | --- | --- | --- | --- | --- | --- | --- | --- | --- | --- | --- | --- | --- | --- | --- | --- | --- | --- | --- | --- | --- | --- | --- | --- | --- | --- |
| Embase   \| No. \| Query \| Results \| Date \| \| --- \| --- \| --- \| --- \| \| #22 \| #9 AND #18 AND #21 \| 354 \| 6-Mar-25 \| \| #21 \| #19 OR #20 \| 34803 \| 6-Mar-25 \| \| #20 \| 'resveratrol':ab,ti OR '3,4,5-stilbenetriol':ab,ti OR '3,5,4-trihydroxystilbene':ab,ti OR '3,4,5-trihydroxystilbene':ab,ti OR 'trans-resveratrol':ab,ti OR 'trans resveratrol':ab,ti OR 'resveratrol-3-sulfate':ab,ti OR 'resveratrol 3 sulfate':ab,ti OR 'srt 501':ab,ti OR 'srt-501':ab,ti OR 'srt501':ab,ti OR 'cis-resveratrol':ab,ti OR 'cis resveratrol':ab,ti OR 'resveratrol, (z)-':ab,ti OR 'trans-resveratrol-3-o-sulfate':ab,ti OR 'trans resveratrol 3 o sulfate':ab,ti \| 23004 \| 6-Mar-25 \| \| #19 \| 'resveratrol'/exp \| 32686 \| 6-Mar-25 \| \| #18 \| #10 OR #11 OR #12 OR #13 OR #14 OR #15 OR #16 OR #17 \| 35365882 \| 6-Mar-25 \| \| #17 \| 'animal experimentation':ab,ti OR 'experimentation, animal':ab,ti OR 'animal experimental use':ab,ti OR 'animal experimental uses':ab,ti OR 'experimental use, animal':ab,ti OR 'experimental uses, animal':ab,ti OR 'animal research':ab,ti OR 'research, animal':ab,ti OR 'animal experiments':ab,ti OR 'animal experiment':ab,ti OR 'experiment, animal':ab,ti OR 'experiments, animal':ab,ti \| 40519 \| 6-Mar-25 \| \| #16 \| 'animal experiment'/exp \| 3316836 \| 6-Mar-25 \| \| #15 \| 'experimental animal':ab,ti OR 'animals, laboratory':ab,ti OR 'animal, laboratory':ab,ti OR 'laboratory animal':ab,ti OR 'laboratory animals':ab,ti \| 40263 \| 6-Mar-25 \| \| #14 \| 'experimental animal'/exp \| 895735 \| 6-Mar-25 \| \| #13 \| 'animal model':ab,ti OR 'models, animal':ab,ti OR 'animal models':ab,ti OR 'model, animal':ab,ti OR 'experimental animal models':ab,ti OR 'animal model, experimental':ab,ti OR 'animal models, experimental':ab,ti OR 'experimental animal model':ab,ti OR 'model, experimental animal':ab,ti OR 'models, experimental animal':ab,ti OR 'laboratory animal models':ab,ti OR 'animal model, laboratory':ab,ti OR 'animal models, laboratory':ab,ti OR 'laboratory animal model':ab,ti OR 'model, laboratory animal':ab,ti OR 'models, laboratory animal':ab,ti \| 301973 \| 6-Mar-25 \| \| #12 \| 'animal model'/exp \| 1939453 \| 6-Mar-25 \| \| #11 \| 'animal':ab,ti OR 'animals':ab,ti OR 'animalia':ab,ti OR 'metazoa':ab,ti \| 1638080 \| 6-Mar-25 \| \| #10 \| 'animal'/exp \| 35036933 \| 6-Mar-25 \| \| #9 \| #1 OR #2 OR #3 OR #4 OR #5 OR #6 OR #7 OR #8 \| 239697 \| 6-Mar-25 \| \| #8 \| 'respiratory distress syndrome':ab,ti OR 'distress syndrome, respiratory':ab,ti OR 'distress syndromes, respiratory':ab,ti OR 'respiratory distress syndromes':ab,ti OR 'syndrome, respiratory distress':ab,ti OR 'shock lung':ab,ti OR 'lung, shock':ab,ti \| 53322 \| 6-Mar-25 \| \| #7 \| 'respiratory distress syndrome'/exp \| 114624 \| 6-Mar-25 \| \| #6 \| 'acute lung injury':ab,ti OR 'acute lung injuries':ab,ti OR 'lung injuries, acute':ab,ti OR 'lung injury, acute':ab,ti \| 25654 \| 6-Mar-25 \| \| #5 \| 'acute lung injury'/exp \| 22089 \| 6-Mar-25 \| \| #4 \| 'fibrosing alveolitis':ab,ti OR 'idiopathic pulmonary fibrosis':ab,ti OR 'idiopathic pulmonary fibroses':ab,ti OR 'pulmonary fibroses, idiopathic':ab,ti OR 'cryptogenic fibrosing alveolitis':ab,ti OR 'cryptogenic fibrosing alveolitides':ab,ti OR 'fibrosing alveolitides, cryptogenic':ab,ti OR 'pulmonary fibrosis, idiopathic':ab,ti OR 'fibrosing alveolitis, cryptogenic':ab,ti OR 'fibrocystic pulmonary dysplasia':ab,ti OR 'dysplasia, fibrocystic pulmonary':ab,ti OR 'fibrocystic pulmonary dysplasias':ab,ti OR 'pulmonary dysplasia, fibrocystic':ab,ti OR 'idiopathic fibrosing alveolitis, chronic form':ab,ti OR 'familial idiopathic pulmonary fibrosis':ab,ti OR 'idiopathic pulmonary fibrosis, familial':ab,ti \| 24096 \| 6-Mar-25 \| \| #3 \| 'fibrosing alveolitis'/exp \| 37878 \| 6-Mar-25 \| \| #2 \| 'lung fibrosis':ab,ti OR 'fibroses, pulmonary':ab,ti OR 'fibrosis, pulmonary':ab,ti OR 'pulmonary fibroses':ab,ti OR 'alveolitis, fibrosing':ab,ti OR 'alveolitides, fibrosing':ab,ti OR 'fibrosing alveolitides':ab,ti OR 'fibrosing alveolitis':ab,ti OR 'idiopathic diffuse interstitial pulmonary fibrosis':ab,ti OR 'pulmonary fibrosis':ab,ti \| 50487 \| 6-Mar-25 \| \| #1 \| 'lung fibrosis'/exp \| 108175 \| 6-Mar-25 \| |
| Web of Science   \| # \| Search Query \| Results \| \| --- \| --- \| --- \| \| 1 \| TS=(Pulmonary Fibrosis OR Fibroses, Pulmonary OR Fibrosis, Pulmonary OR Pulmonary Fibroses OR Alveolitis, Fibrosing OR Alveolitides, Fibrosing OR Fibrosing Alveolitides OR Fibrosing Alveolitis OR Idiopathic Diffuse Interstitial Pulmonary Fibrosis) \| 44928 \| \| 2 \| TS=(Idiopathic Pulmonary Fibrosis OR Idiopathic Pulmonary Fibroses OR Pulmonary Fibroses, Idiopathic OR Cryptogenic Fibrosing Alveolitis OR Cryptogenic Fibrosing Alveolitides OR Fibrosing Alveolitides, Cryptogenic OR Pulmonary Fibrosis, Idiopathic OR Fibrosing Alveolitis, Cryptogenic OR Fibrocystic Pulmonary Dysplasia OR Dysplasia, Fibrocystic Pulmonary OR Fibrocystic Pulmonary Dysplasias OR Pulmonary Dysplasia, Fibrocystic OR Idiopathic Fibrosing Alveolitis, Chronic Form OR Familial Idiopathic Pulmonary Fibrosis OR Idiopathic Pulmonary Fibrosis, Familial) \| 17840 \| \| 3 \| TS=(Acute Lung Injury OR Acute Lung Injuries OR Lung Injuries, Acute OR Lung Injury, Acute) \| 30255 \| \| 4 \| TS=(Respiratory Distress Syndrome OR Distress Syndrome, Respiratory OR Distress Syndromes, Respiratory OR Respiratory Distress Syndromes OR Syndrome, Respiratory Distress Shock Lung OR Lung, Shock) \| 37549 \| \| 5 \| #4 OR #3 OR #2 OR #1 \| 98483 \| \| 6 \| TS=(Animals OR Animal OR Animalia OR Metazoa) \| 766024 \| \| 7 \| TS=(Models, Animal OR Animal Model OR Animal Models OR Model, Animal OR Experimental Animal Models OR Animal Model, Experimental OR Animal Models, Experimental OR Experimental Animal Model OR Model, Experimental Animal OR Models, Experimental Animal OR Laboratory Animal Models OR Animal Model, Laboratory OR Animal Models, Laboratory OR Laboratory Animal Model OR Model, Laboratory Animal OR Models, Laboratory Animal) \| 320194 \| \| 8 \| TS=(Animals, Laboratory OR Animal, Laboratory OR Laboratory Animal OR Laboratory Animals) \| 30718 \| \| 9 \| TS=(Animal Experimentation OR Experimentation, Animal OR Animal Experimental Use OR Animal Experimental Uses OR Experimental Use, Animal OR Experimental Uses, Animal OR Animal Research OR Research, Animal OR Animal Experiments OR Animal Experiment OR Experiment, Animal OR Experiments, Animal) \| 200108 \| \| 10 \| #9 OR #8 OR #7 OR #6 \| 766024 \| \| 11 \| TS=(Resveratrol OR (3,4',5-Stilbenetriol) OR (3,5,4'-Trihydroxystilbene) OR (3,4',5-Trihydroxystilbene) OR (trans-Resveratrol) OR (trans Resveratrol) OR (Resveratrol-3-sulfate) OR Resveratrol 3 sulfate OR SRT 501 OR (SRT-501) OR SRT501 OR (cis-Resveratrol) OR cis Resveratrol OR (Resveratrol, (Z)-) OR (trans-Resveratrol-3-O-sulfate) OR trans Resveratrol 3 O sulfate) \| 24586 \| \| 12 \| #11 AND #10 AND #5 \| 32 \| |
| **Cochrane Library**  Date Run: 06/03/2025 23:08:31  Comment:  ID Search Hits  #1 MeSH descriptor: [Pulmonary Fibrosis] explode all trees 912  #2 (Pulmonary Fibrosis):ti,ab,kw OR (Fibroses, Pulmonary):ti,ab,kw OR (Fibrosis, Pulmonary):ti,ab,kw OR (Pulmonary Fibroses):ti,ab,kw OR (Alveolitis, Fibrosing):ti,ab,kw 4664  #3 (Alveolitides, Fibrosing):ti,ab,kw OR (Fibrosing Alveolitides):ti,ab,kw OR (Fibrosing Alveolitis):ti,ab,kw OR (Idiopathic Diffuse Interstitial Pulmonary Fibrosis):ti,ab,kw 921  #4 MeSH descriptor: [Idiopathic Pulmonary Fibrosis] explode all trees 594  #5 (Idiopathic Pulmonary Fibrosis):ti,ab,kw OR (Idiopathic Pulmonary Fibroses):ti,ab,kw OR (Pulmonary Fibroses, Idiopathic):ti,ab,kw OR (Cryptogenic Fibrosing Alveolitis):ti,ab,kw OR (Cryptogenic Fibrosing Alveolitides):ti,ab,kw 1708  #6 (Fibrosing Alveolitides, Cryptogenic):ti,ab,kw OR (Pulmonary Fibrosis, Idiopathic):ti,ab,kw OR (Fibrosing Alveolitis, Cryptogenic):ti,ab,kw OR (Fibrocystic Pulmonary Dysplasia):ti,ab,kw OR (Dysplasia, Fibrocystic Pulmonary):ti,ab,kw 1708  #7 (Fibrocystic Pulmonary Dysplasias):ti,ab,kw OR (Pulmonary Dysplasia, Fibrocystic):ti,ab,kw OR (Idiopathic Fibrosing Alveolitis, Chronic Form):ti,ab,kw OR (Familial Idiopathic Pulmonary Fibrosis):ti,ab,kw OR (Idiopathic Pulmonary Fibrosis, Familial):ti,ab,kw 23  #8 MeSH descriptor: [Acute Lung Injury] explode all trees 696  #9 (Acute Lung Injury):ti,ab,kw OR (Acute Lung Injuries):ti,ab,kw OR (Lung Injuries, Acute):ti,ab,kw OR (Lung Injury, Acute):ti,ab,kw 2895  #10 MeSH descriptor: [Respiratory Distress Syndrome] explode all trees 3576  #11 (Respiratory Distress Syndrome):ti,ab,kw OR (Distress Syndrome, Respiratory):ti,ab,kw OR (Distress Syndromes, Respiratory):ti,ab,kw OR (Respiratory Distress Syndromes):ti,ab,kw OR (Syndrome, Respiratory Distress):ti,ab,kw 7495  #12 (Shock Lung):ti,ab,kw OR (Lung, Shock):ti,ab,kw 957  #13 #1 OR #2 OR #3 OR #4 OR #5 OR #6 OR #7 OR #8 OR #9 OR #10 OR #11 OR #12 14304  #14 MeSH descriptor: [Animation] explode all trees 0  #15 (Animals):ti,ab,kw OR (Animal):ti,ab,kw OR (Animalia):ti,ab,kw OR (Metazoa):ti,ab,kw 40965  #16 MeSH descriptor: [Models, Animal] explode all trees 1155  #17 (Models, Animal):ti,ab,kw OR (Animal Model):ti,ab,kw OR (Animal Models):ti,ab,kw OR (Model, Animal):ti,ab,kw OR (Experimental Animal Models):ti,ab,kw 11808  #18 (Animal Model, Experimental):ti,ab,kw OR (Animal Models, Experimental):ti,ab,kw OR (Experimental Animal Model):ti,ab,kw OR (Model, Experimental Animal):ti,ab,kw OR (Models, Experimental Animal):ti,ab,kw 1571  #19 (Laboratory Animal Models):ti,ab,kw OR (Animal Model, Laboratory):ti,ab,kw OR (Animal Models, Laboratory):ti,ab,kw OR (Laboratory Animal Model):ti,ab,kw OR (Model, Laboratory Animal):ti,ab,kw 684  #20 (Models, Laboratory Animal):ti,ab,kw 463  #21 MeSH descriptor: [Animals, Laboratory] explode all trees 752  #22 (Animals, Laboratory):ti,ab,kw OR (Animal, Laboratory):ti,ab,kw OR (Laboratory Animal):ti,ab,kw OR (Laboratory Animals):ti,ab,kw 2094  #23 MeSH descriptor: [Animal Experimentation] explode all trees 11  #24 (Animal Experimentation):ti,ab,kw OR (Experimentation, Animal):ti,ab,kw OR (Animal Experimental Use):ti,ab,kw OR (Animal Experimental Uses):ti,ab,kw OR (Experimental Use, Animal):ti,ab,kw 1039  #25 (Experimental Uses, Animal):ti,ab,kw OR (Animal Research):ti,ab,kw OR (Research, Animal):ti,ab,kw OR (Animal Experiments):ti,ab,kw OR (Animal Experiment):ti,ab,kw 8921  #26 (Experiment, Animal):ti,ab,kw OR (Experiments, Animal):ti,ab,kw 6435  #27 #14 OR #15 OR #16 OR #17 OR #18 OR #19 OR #20 OR #21 OR #22 OR #23 OR #24 OR #25 OR #26 40974  #28 MeSH descriptor: [Resveratrol] explode all trees 417  #29 (Resveratrol):ti,ab,kw OR (3,4,5Stilbenetriol):ti,ab,kw OR (3,5,4Trihydroxystilbene):ti,ab,kw OR (3,4,5Trihydroxystilbene):ti,ab,kw OR (trans Resveratrol):ti,ab,kw 827  #30 (Resveratrol 3 sulfate):ti,ab,kw OR (SRT501):ti,ab,kw OR (cis Resveratrol):ti,ab,kw OR (trans Resveratrol 3 O sulfate):ti,ab,kw 14  #31 #28 OR #29 OR #30 827  #32 #31 AND #27 AND #13 1 |
| CNKI 69  检索式：  （主题：白藜芦醇 + 白黎芦醇）AND（主题：特发性肺纤维化 + 特发性肺间质纤维化 + 肺纤维化 + 肺间质纤维化) |
| Wanfang 28  检索式：  题名或关键词:(白藜芦醇 OR 白黎芦醇) and 题名或关键词:(特发性肺纤维化 OR 特发性肺间质纤维化 OR 肺纤维化 OR 肺间质纤维化) |
| VIP 20  检索式：  ((题名或关键词=白藜芦醇 OR 题名或关键词=白黎芦醇) AND (((题名或关键词=特发性肺纤维化 OR 题名或关键词=特发性肺间质纤维化) OR 题名或关键词=肺纤维化) OR 题名或关键词=肺间质纤维化)) |
| SinoMed 63  ( "白藜芦醇"[常用字段:智能] OR "白黎芦醇"[常用字段:智能]) AND( "特发性肺纤维化"[常用字段:智能] OR "特发性肺间质纤维化"[常用字段:智能] OR "肺纤维化"[常用字段:智能] OR "肺间质纤维化"[常用字段:智能]) |

## Supplementary Table S2 Characteristics of the included studies

| NO | First Author | Year | Country | Type of animal | Weight | Age | Gender | Sample size(I/C) | |
| --- | --- | --- | --- | --- | --- | --- | --- | --- | --- |
| 1 | Göksel Şener | 2007 | Turkey | Wistar albino rat | 200-250 g | / | Male | 8 | 8 |
| 2 | Cao Guowen | 2008 | China | Kunming mice | 18-22 g | / | / | 30 | 30 |
| 3 | Wang Zaiyan(1) | 2011 | China | SD rat | 200±20 g | / | Female | 15/15/15 | 15 |
| 4 | Wang Zaiyan(2) | 2011 | China | SD rat | 200±20 g | / | Female | 15/15/15 | 15 |
| 5 | Zhang Yan | 2011 | China | SD rat | 240-280 g | 3 months old | Male and Female | 32/32/32 | 32 |
| 6 | He Pingping | 2012 | China | SD rat | 200±20 g | 3-4 months old | Female | 15/15/15 | 15 |
| 7 | Li Wanshuang | 2012 | China | SD rat | 200±20 g | / | Male | 15/15 | 15 |
| 8 | Recep Akgedik | 2012 | Turkey | Wistar albino rat | 200-250 g | Adult | Male | 7 | 7 |
| 9 | Liu Lijing | 2013 | China | SD rat | 220±7.5 g | 3-4 months old | Male | 40 | 40 |
| 10 | Zhang Yunqian | 2014 | China | ICR mice | 23-28 g | 7-9 weeks old | Male | 7 | 7 |
| 11 | Li Yingchun | 2015 | China | SD rat | 200±20 g | 6-7 weeks old | Male | 21 | 21 |
| 12 | Daniela Impellizzeri | 2015 | Italy | CD-1 mice | 25-35 g | / | Male | 10 | 10 |
| 13 | Jin Su | 2016 | China | Kunming mice | 18-22 g | / | Male | 18 | 12 |
| 14 | Li Lihua | 2016 | China | SD rat | / | 5 weeks old | Male | 10/10 | 10 |
| 15 | Xu Bo | 2017 | China | SD rat | 268.96±105.64 g | 3.28±1.20 months old | Male | 36 | 36 |
| 16 | Koyuncu Ismail | 2018 | Turkey | BALB/C mice | 25-30 g | / | Male and Female | 10/10 | 10 |
| 17 | Wang Jing | 2018 | China | SD rat | / | 6-8 weeks old | Male and Female | 6 | 6 |
| 18 | Ding Shibin | 2019 | China | C57BL/6J mice | 18-23 g | 6 weeks old | Male | 10/10 | 10 |
| 19 | Rasoul Yahyapour | 2019 | Iran | NMRI mice | / | / | Male | 10 | 10 |
| 20 | Rasoul Azmoonfar | 2019 | Iran | NMRI mice | / | / | / | 5 | 5 |
| 21 | Liu Yanlu | 2020 | China | SD rat | 180-220 g | 6 weeks old | Male | 6 | 6 |
| 22 | Wang Zaiyan(3) | 2021 | China | SD rat | / | / | / | 15/15/15 | 15 |
| 23 | Wang Lei | 2022 | China | SD rat | 200±20 g | / | Male | 12/12/12 | 12 |
| 24 | Quan Yifan | 2023 | China | SD rat | 180-220 g | / | Male | 6/6 | 6 |
| 25 | Jiang Yunfei | 2024 | China | C57BL/6J mice | 20-25 g | 8 weeks old | Male | 10 | 10 |

Continued Supplementary Table S2 Characteristics of the included studies

| NO | First Author | Modeling methods | Treatment(I/C) | | Res administration method | Res sources | Purity of Res | Treatment time | Specimen | Outcome |  |
| --- | --- | --- | --- | --- | --- | --- | --- | --- | --- | --- | --- |
| 1 | Göksel Şener | Single intratracheal BLM (5 mg/kg) | Res 10 mg/kg/d | NS 1 ml/kg/d | Oral administration | Mikrogen Pharmaceutical,Istanbul,Turkey | / | 14 days | BALF;Lung tissue | ①⑤⑦⑧⑨⑩⑪ | means±SEM |
| 2 | Cao Guowen | Single intratracheal BLM (5 mg/kg) | Res 100 mg/kg/d | NS 10 ml/kg/d | Oral gavage | Xi'an Tianyi Biotechnology Co.,Ltd.,China | ≥98% | 7/14/28 days | Lung tissue | ①②④⑩⑫ | mean±SD |
| 3 | Wang Zaiyan(1) | Single intratracheal BLM (5 mg/kg) | Res 25/50/100 mg/kg/d | NS 10 ml/kg/d | Oral gavage | Xi'an Tianyi Biotechnology Co.,Ltd.,China | ≥98% | 7/14/28 days | Lung tissue | ②⑥ | mean±SD |
| 4 | Wang Zaiyan(2) | Single intratracheal BLM (5 mg/kg) | Res 25/50/100 mg/kg/d | NS 10 ml/kg/d | Oral gavage | Xi'an Tianyi Biotechnology Co.,Ltd.,China | ≥98% | 7/14/28 days | Lung tissue | ⑥ | mean±SD |
| 5 | Zhang Yan | Single intratracheal BLM (5 mg/kg) | Res 10/20/40 mg/kg/d | NS 1 ml/kg/d | Intraperitoneal injection | Shaanxi Saide Gaoke Biotechnology Co.,Ltd.,China | / | 3/7/14/28 days | BALF;Lung tissue | ②⑤ | mean±SD |
| 6 | He Pingping | Single intratracheal BLM (5 mg/kg) | Res 25/50/100 mg/kg/d | NS 10 ml/kg/d | Oral gavage | Xi'an Tianyi Biotechnology Co.,Ltd.,China | ≥98% | 7/14/28 days | Lung tissue | ② | mean±SD |
| 7 | Li Wanshuang | Single intratracheal BLM (5 mg/kg) | Res 50/100 mg/kg/d | NS 10 ml/kg/d | Oral gavage | Xi'an Tianyi Biotechnology Co.,Ltd.,China | ≥98% | 17/24/31 days | Lung tissue | ②⑤ | mean±SD |
| 8 | Recep Akgedik | Single intratracheal BLM (2.5 mg/kg) | Res 10 mg/kg/d | NS | Oral gavage | Terraternal pharmaceutical,London,England | / | 14 days | BALF;Lung tissue;Serum | ①②⑩ | mean±SD |
| 9 | Liu Lijing | Single intratracheal BLM (5 mg/kg) | Res 100 mg/kg/d | NS 10 ml/kg/d | Oral gavage | Xi'an Tianyi Biotechnology Co.,Ltd.,China | ≥98% | 7/14/28/56 days | Lung tissue;Serum | ①③④⑤ | mean±SD |
| 10 | Zhang Yunqian | Single intratracheal LPS (5 mg/kg) | Res 0.3 mg/kg/d | NS | Intraperitoneal injection | Sigma-Aldrich,USA | / | 28 days | Lung tissue | ②③⑤⑩⑫ | means±SEM |
| 11 | Li Yingchun | Single intratracheal BLM (5 mg/kg) | Res 50 mg/kg/d | NS | Oral gavage | Xi'an Tianyi Biotechnology Co.,Ltd.,China | ≥98% | 7/14/21 days | Lung tissue | ①④⑤⑥ | mean±SD |
| 12 | Daniela Impellizzeri | Single intratracheal BLM (1 mg/kg) | Res 50 mg/kg/d | NS | Oral administration | PoliNat SL,Spain | / | 7 days | BALF;Lung tissue | ①⑪ | means±SEM |
| 13 | Jin Su | Single intratracheal BLM (3.5 mg/kg) | Res 100 mg/kg/d | NS | Oral gavage | Baoji Guokang Biotechnology Co.,Ltd.,China | 0.98 | 14/28 days | Lung tissue | ② | mean±SD |
| 14 | Li Lihua | Single intratracheal BLM (4 mg/kg) | Res 25/50 mg/kg/d | NS | Oral gavage | Sigma-Aldrich,USA | / | 30 days | Lung tissue | ②③ | mean±SD |
| 15 | Xu Bo | Single intratracheal BLM (5 mg/kg) | Res 50 mg/kg/d | NS 10 ml/kg/d | Oral gavage | Xi'an Tianyi Biotechnology Co.,Ltd.,China | ≥98% | 7 days | Lung tissue;Serum | ①②③④⑤⑦⑨⑩⑫ | mean±SD |
| 16 | Koyuncu Ismail | Single intraperitoneal BLM(10 mg/kg) | Res 25/50 mg/kg/d | NS | Intraperitoneal injection | / | / | 6 days | Lung tissue;Serum | ①②⑩⑪⑫ | mean±SD |
| 17 | Wang Jing | Single intraperitoneal BLM(5 mg/kg) | Res 60 mg/kg/d | NS | Intraperitoneal injection | Sigma-Aldrich,USA | / | 28 days | Lung tissue | ①②④ | mean±SD |
| 18 | Ding Shibin | exposed to ambient PM | Res 50/100 mg/kg/d | Deionized Water | Oral gavage | Sigma-Aldrich,USA | / | 140 days | BALF;Lung tissue | ①⑤⑦⑧⑨ | mean±SD |
| 19 | Rasoul Yahyapour | Cobalt-60 γ-ray thoracic irradiation (18 Gy, 94 cGy/min) | Res 200 mg/kg/d | γ rays:18 Gy | Oral gavage | NanoKimia company,Iran. | / | 80 days | Lung tissue | ① | mean±SD |
| 20 | Rasoul Azmoonfar | Cobalt-60 γ-ray thoracic irradiation (18 Gy, SSD 80 cm, 60 cGy/min) | Res 100 mg/kg/d | γ rays:18 Gy | Oral gavage | NanoKimia company,Iran. | / | 100 days | Lung tissue | ①④ | mean±SD |
| 21 | Liu Yanlu | Single intraperitoneal BLM(5 mg/kg) | Res 40 mg/kg/d | NS | Oral gavage | Guangzhou Honsea Sunshine Biotech Co., Ltd.,China | >99% | 28 days | Lung tissue | ①②③⑦⑨⑩⑪⑫ | mean±SD |
| 22 | Wang Zaiyan(3) | Single intratracheal BLM (5 mg/kg) | Res 25/50/100 mg/kg/d | NS 10 ml/kg/d | / | / | / | 7/14/28 days | Lung tissue | ⑥ | mean±SD |
| 23 | Wang Lei | Single intratracheal BLM (5 mg/kg) | Res 25/50/100 mg/kg/d | NS | Oral gavage | Chengdu Must Bio-Technology Co., Ltd.,China | ≥98% | 7/14 days | Lung tissue | ①⑤⑥⑦⑧⑨⑫ | mean±SD |
| 24 | Quan Yifan | Single intratracheal SiO_2_ (50 mg/ml) | Res 50/100 mg/kg/d | NS 1ml/d | / | Shanghai Aladdin Co.,China | 99% | 28 days | Lung tissue | ①②③④⑤⑦⑧ | mean±SD |
| 25 | Jiang Yunfei | Single intratracheal BLM (5 mg/kg) | Res 100 mg/kg/d | NS 10 ml/kg/d | Oral gavage | / | / | 28 days | Lung tissue | ①②④⑤⑦⑧⑨⑩⑫ | mean±SD |

Note: Res_resveratrol; BLM_bleomycin; LPS_lipopolysaccharide; NS_normal saline; BALF_bronchoalveolar lavage fluid.
①_Pulmonary fibrosis score;②_Hydroxyproline(Hyp) content;③_Collagen 1 (Col 1) content;④_Alveolitis score;⑤_Transforming growth factor-β(TGF-β) content;⑥_Nuclear factor kappa-B(NF-κB) content;⑦_Tumor necrosis factor-α(TNF-α) content;⑧_Interleukin-1β(IL-1β) content;⑨_Interleukin-6(IL-6) content;⑩Malondialdehyde(MDA) content;⑪Myeloperoxidase(MPO) content;⑫Superoxide Dismutase(SOD) content.

# Supplementary Figures

## Supplementary Figure 1 Subgroup analysis of Pulmonary fibrosis score

### (A) According to animal strain


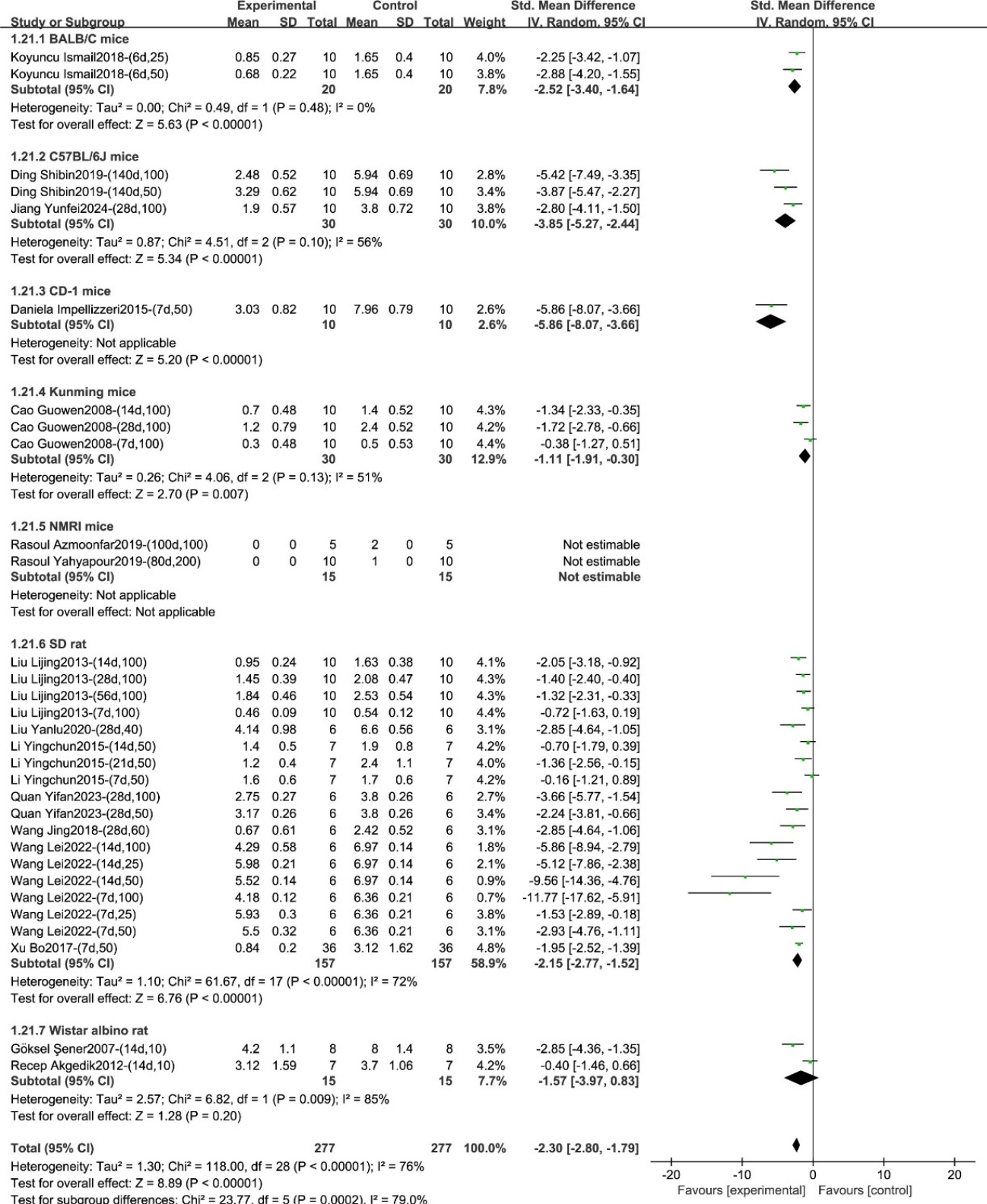


### (B) According to RES drug source


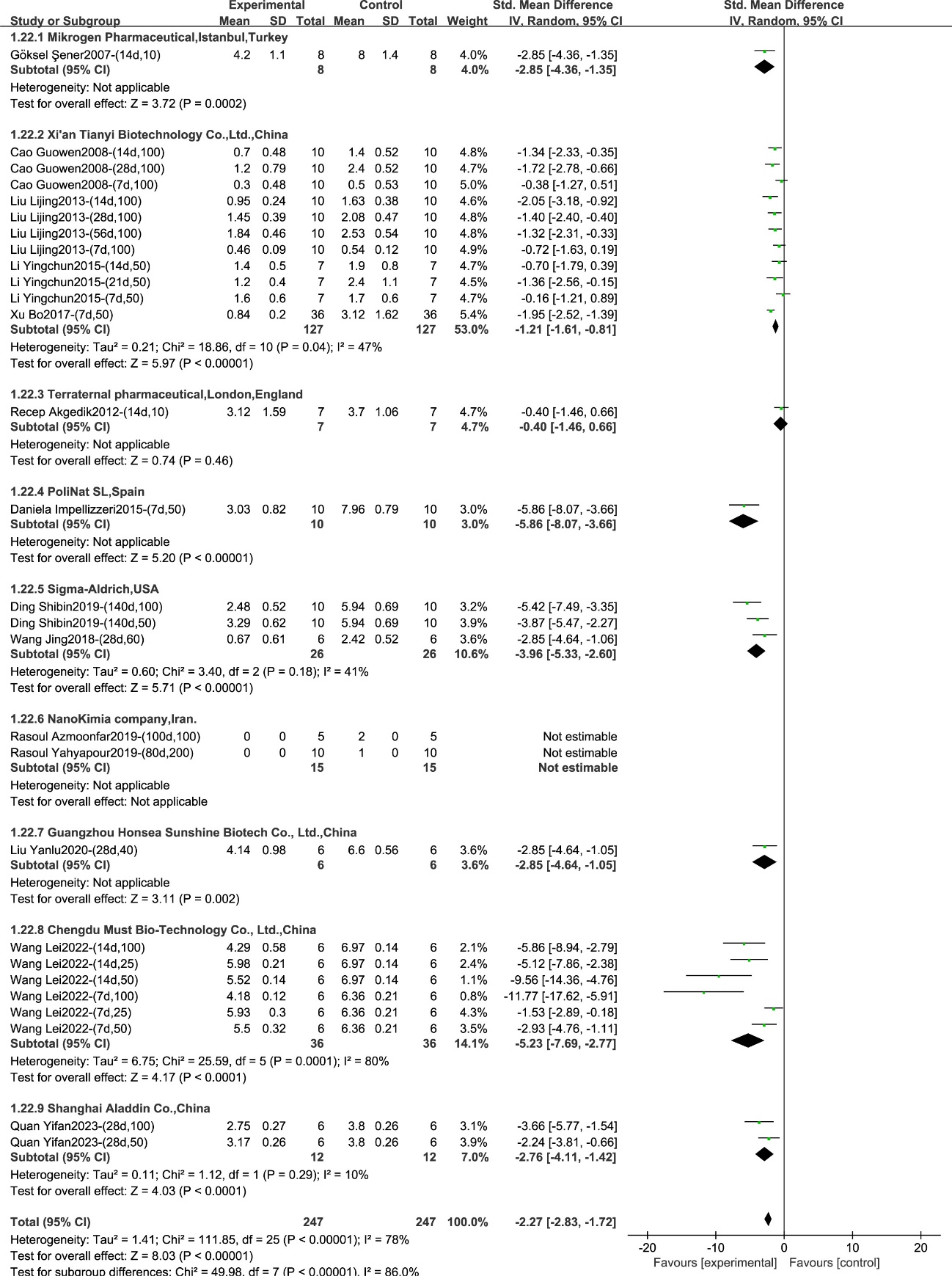


### (C) According to pulmonary fibrosis modeling method


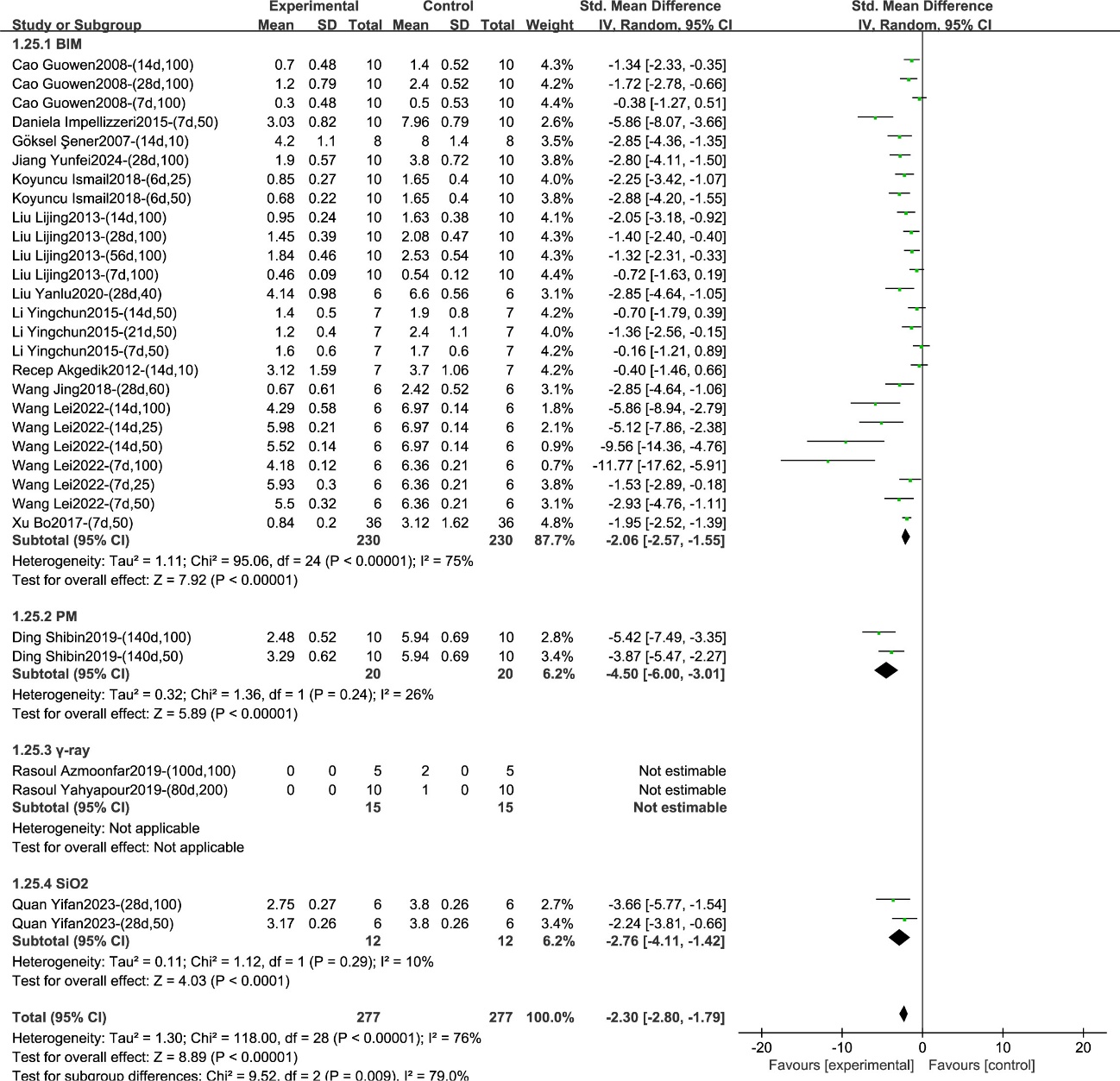


### (D) According to RES administration route


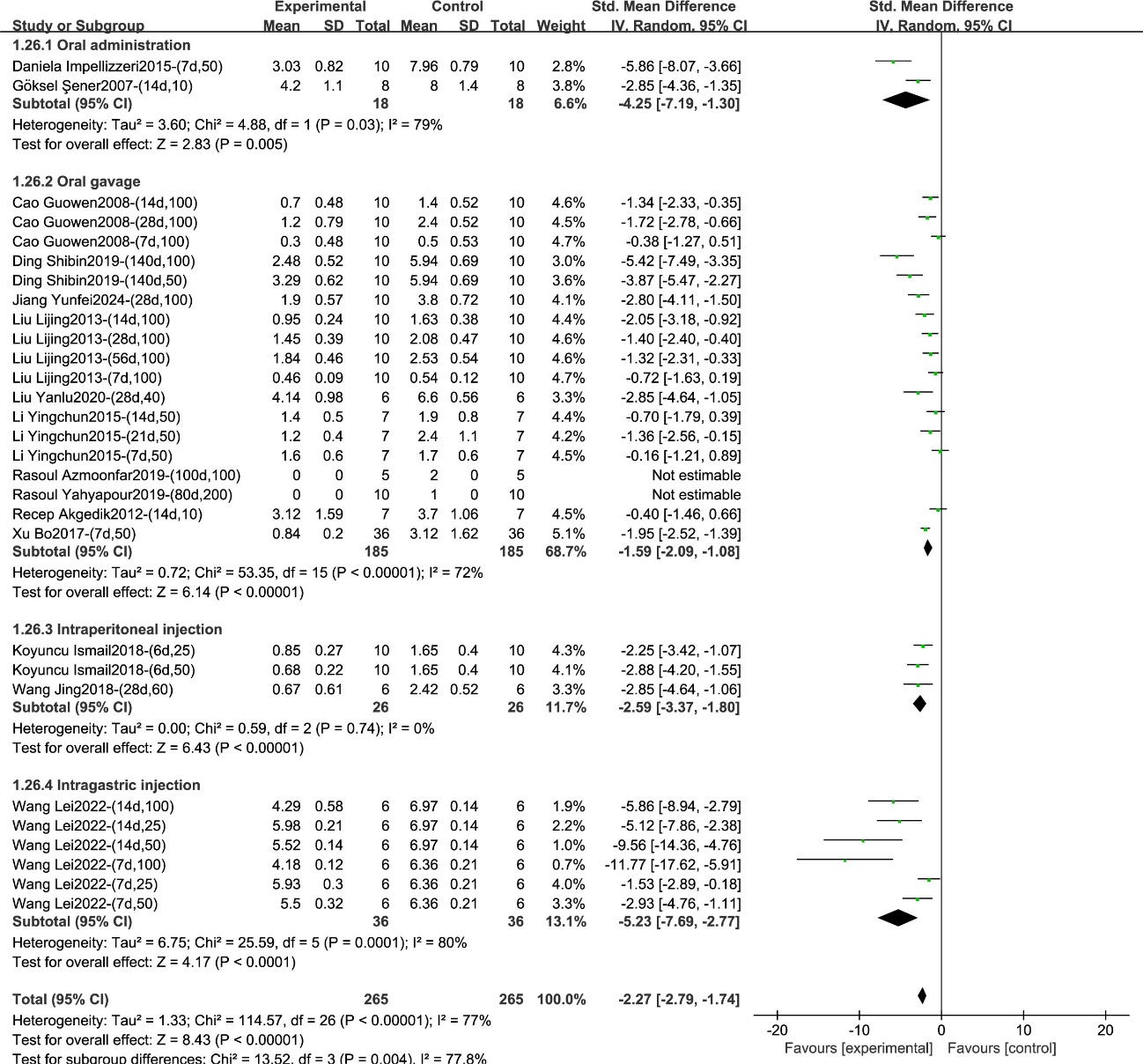


## Supplementary Figure 2 Subgroup analysis of Hyp content

### (A) According to RES drug source


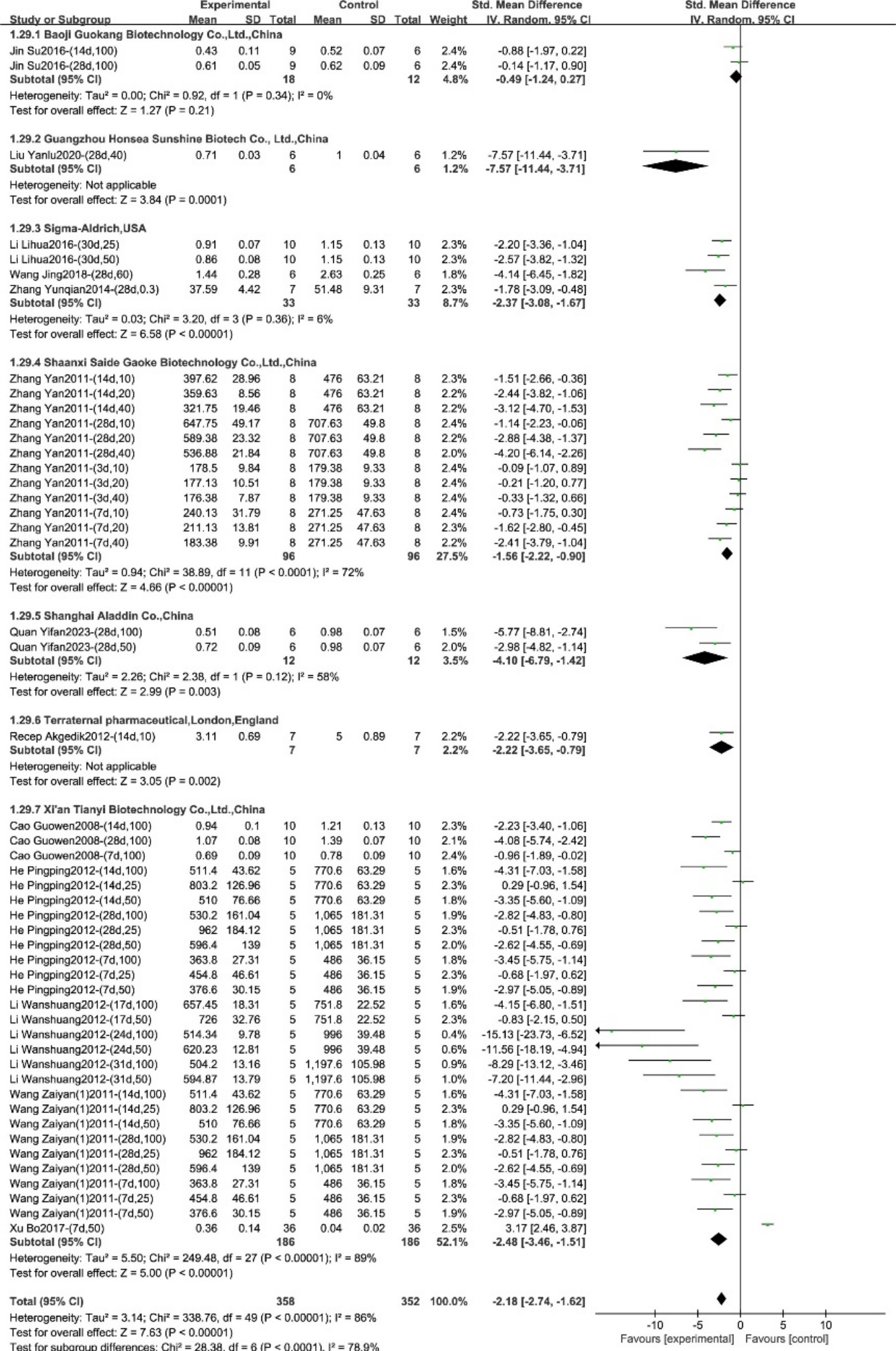


### (B) According to RES dosage


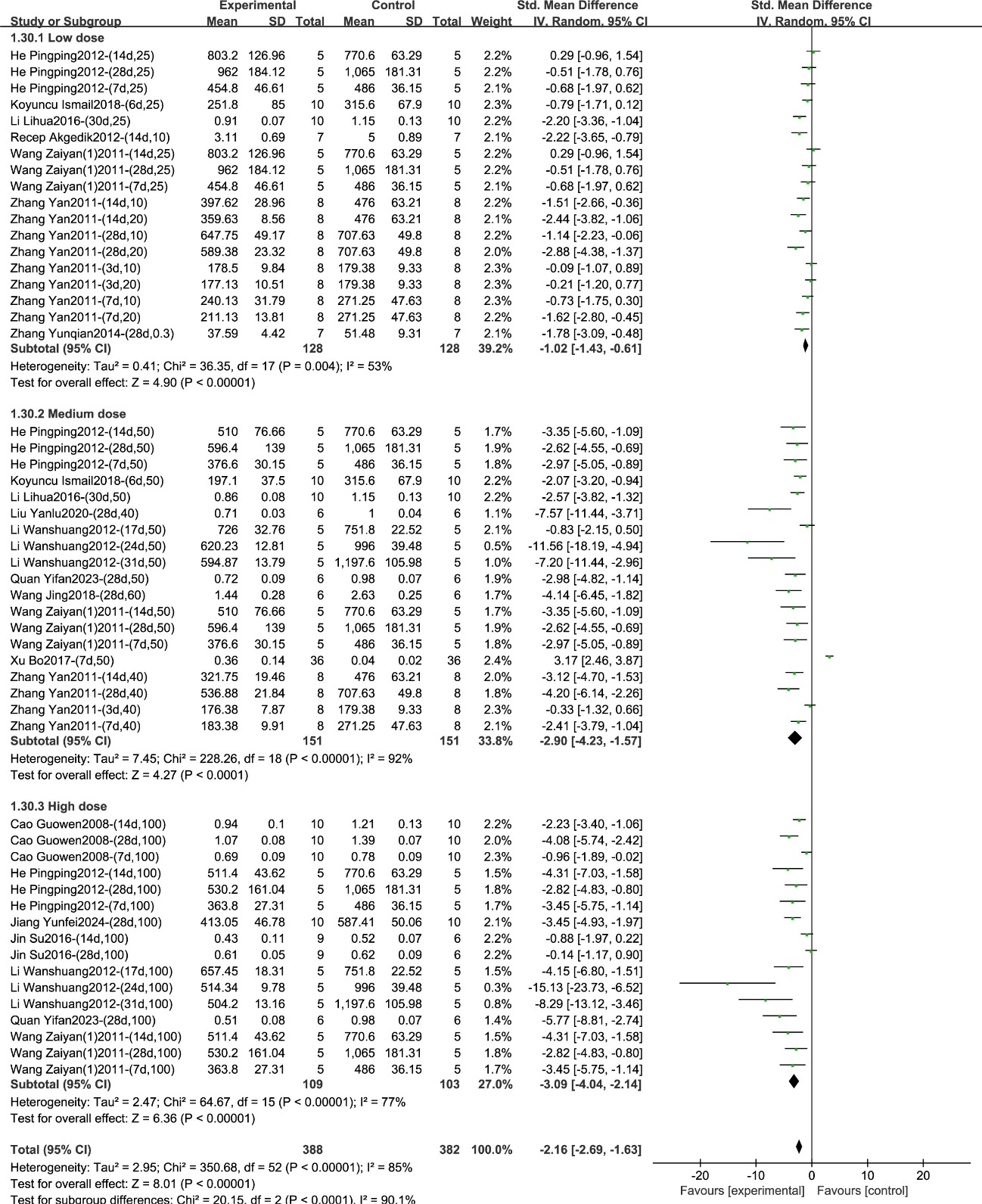


### (C) According to RES intervention duration


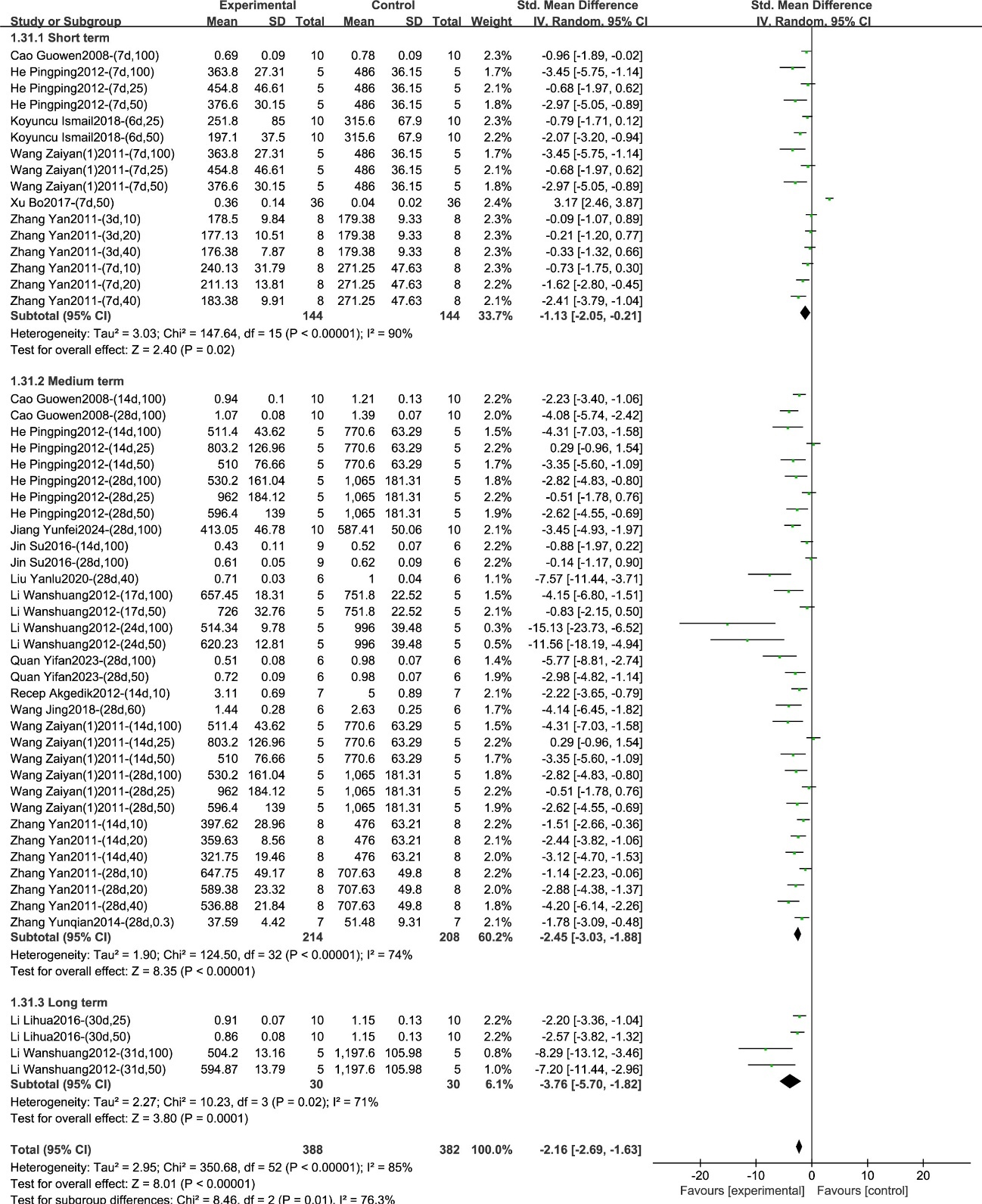


## Supplementary Figure 3 Effect of RES on Col 1 content in lung tissues of PF animals

### (A) Forest plot of Col 1 content


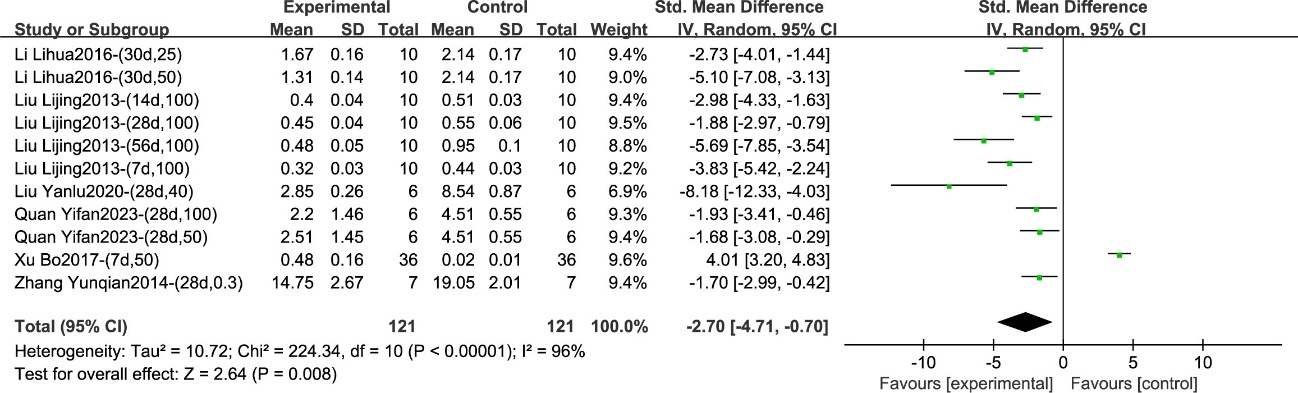


### (B) Sensitivity analysis of Col 1 content


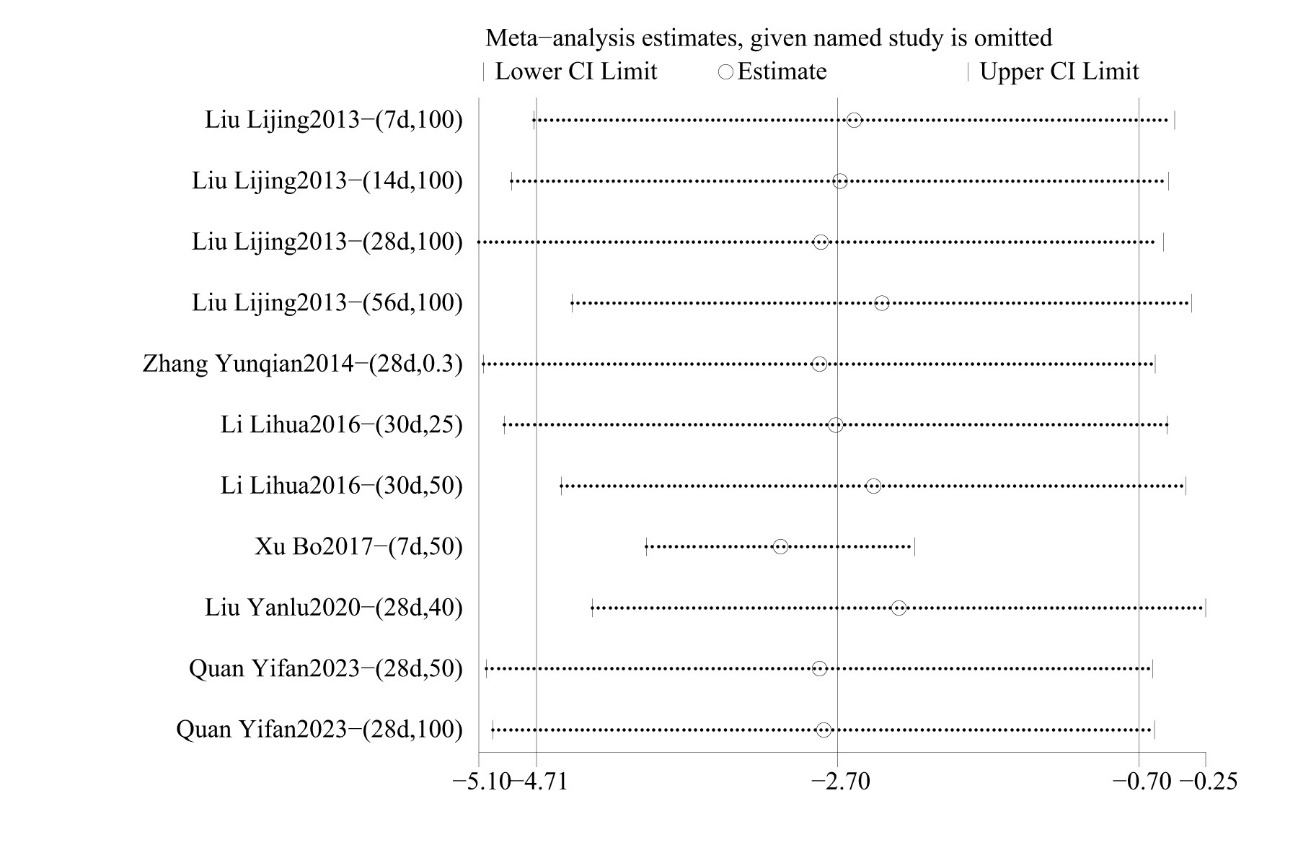


### (C) Funnel plot of Col 1 content


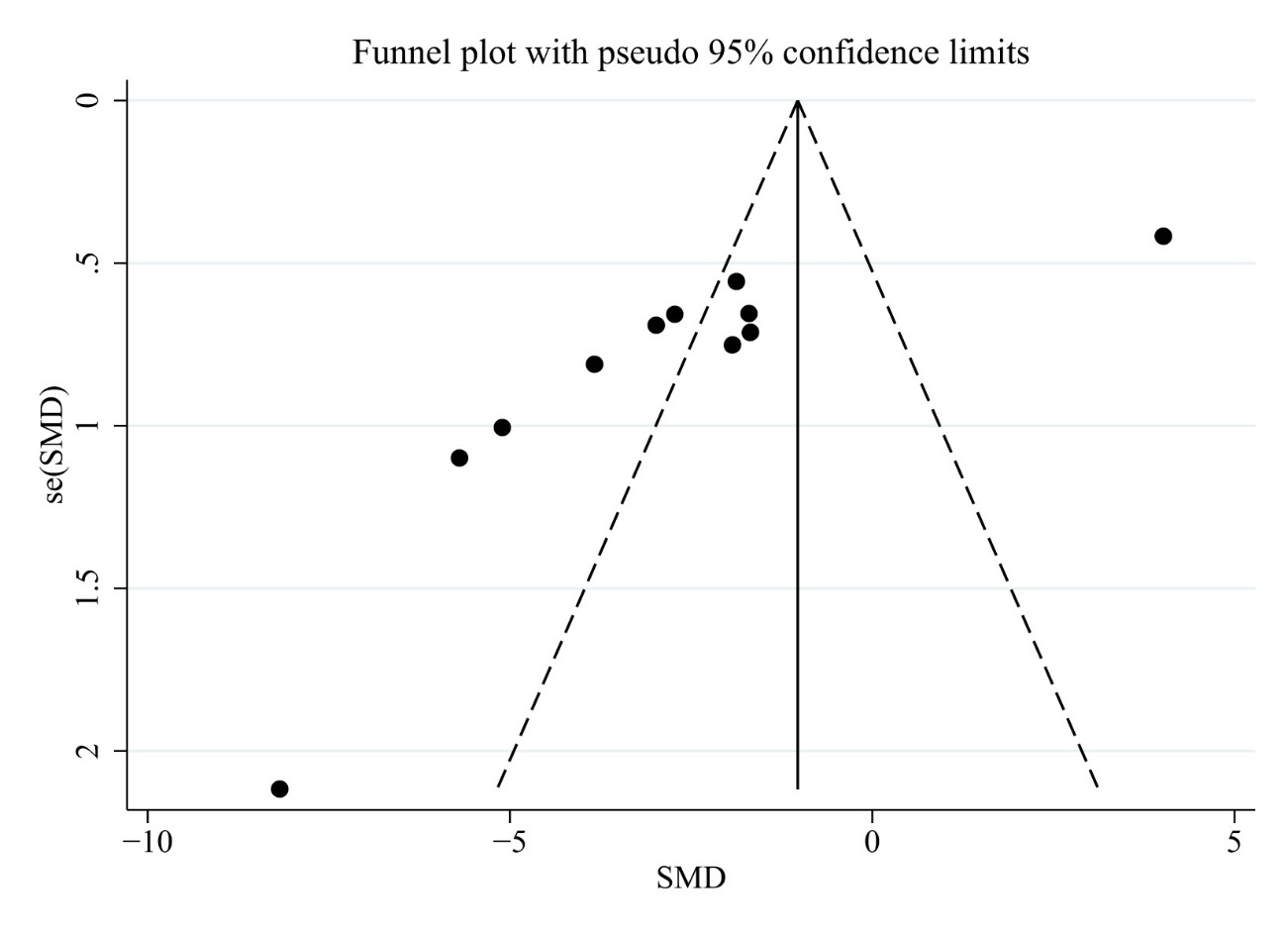


### (D) Egger test for Col 1 content


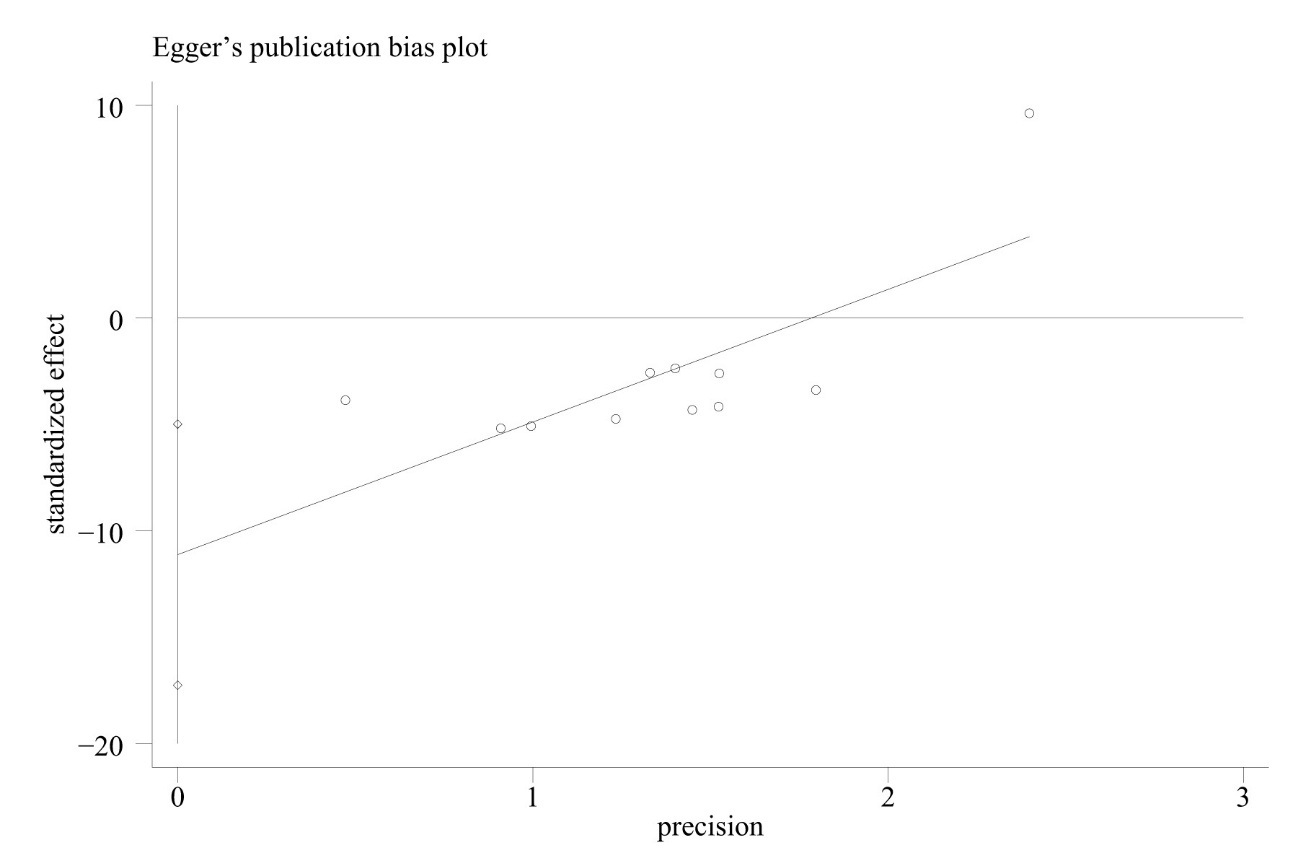


## Supplementary Figure 4 Subgroup analysis of Col 1 content according to RES drug source


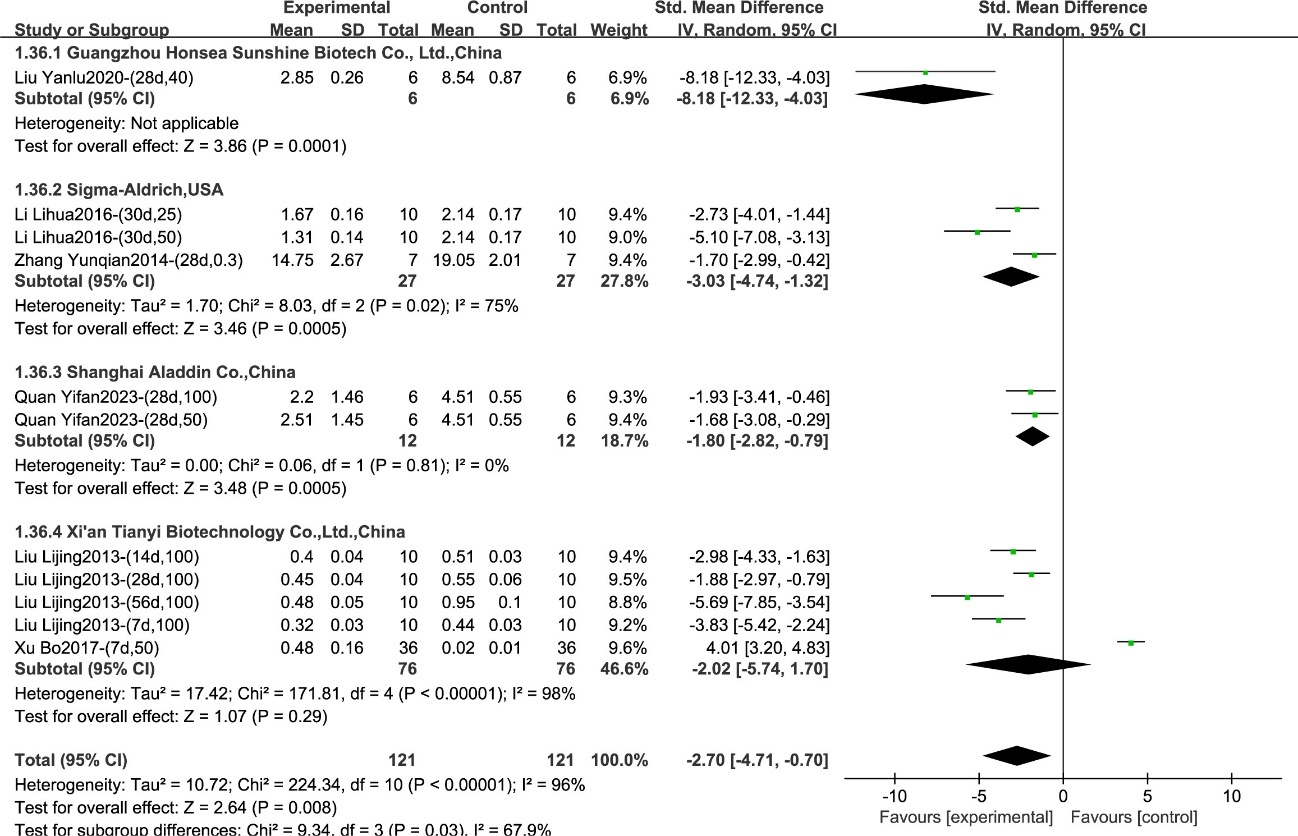


## Supplementary Figure 5 Subgroup analysis of Alveolitis score

### (A) According to animal strain


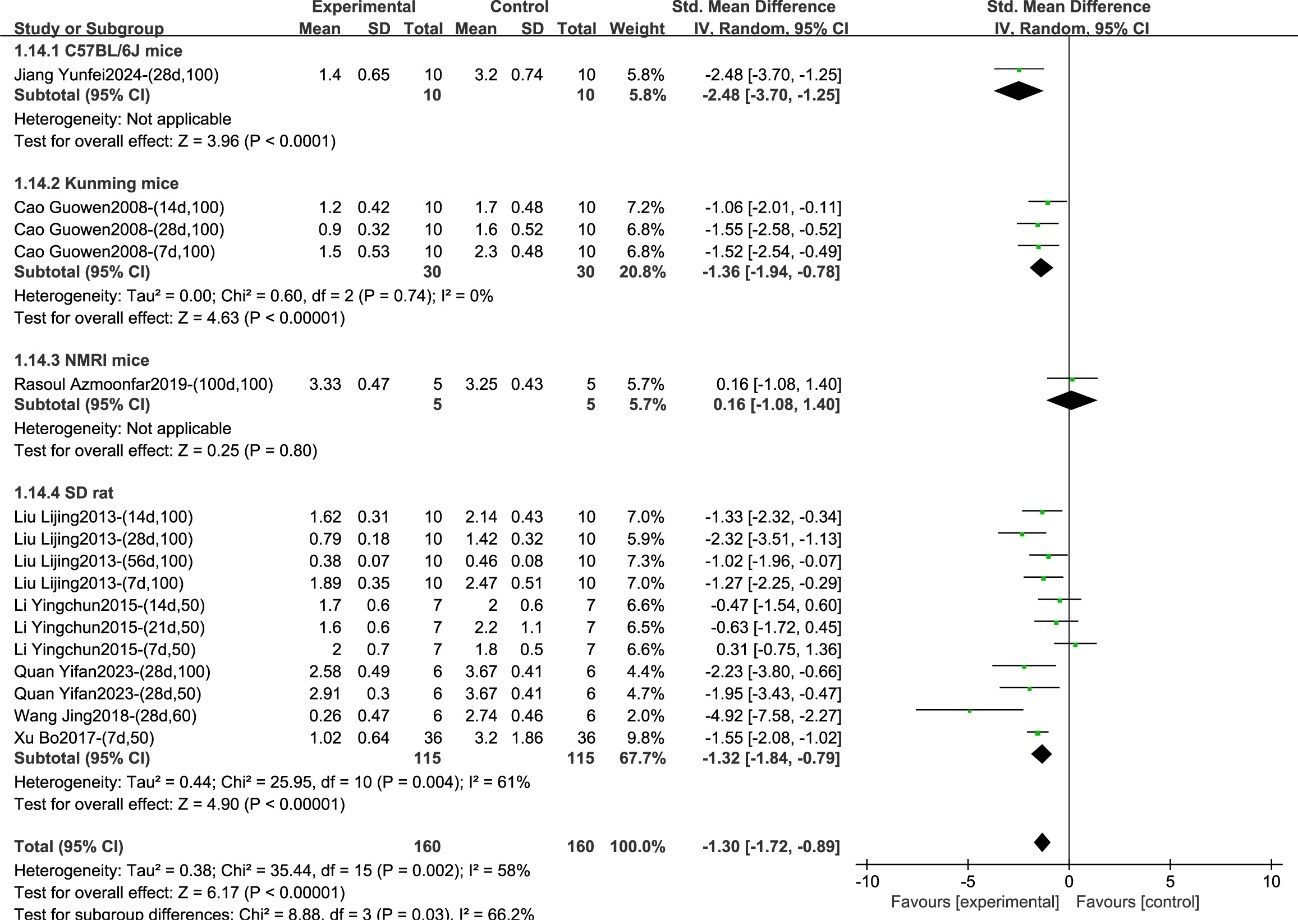


### (B) According to RES drug source


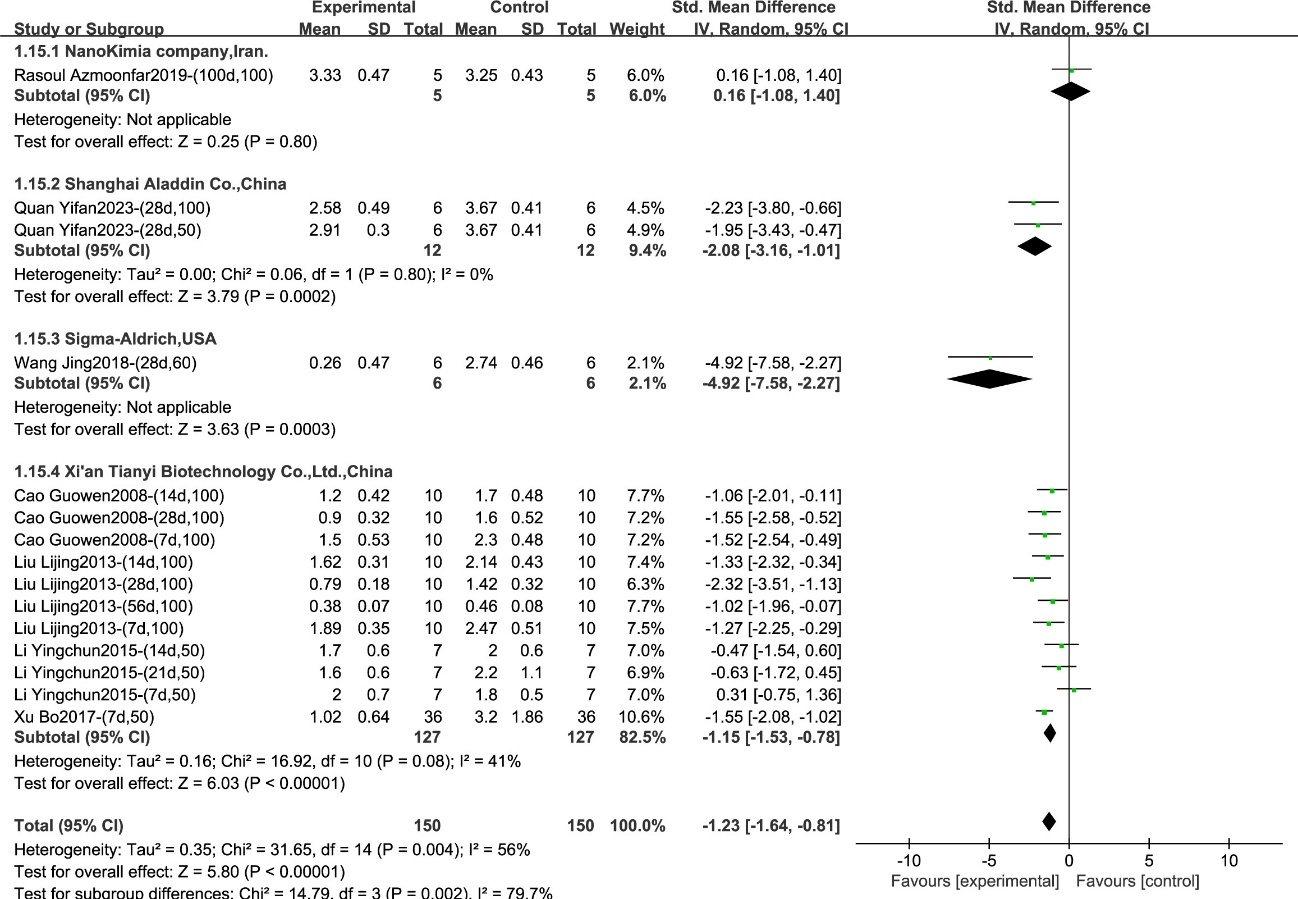


### (C) According to pulmonary fibrosis modeling method


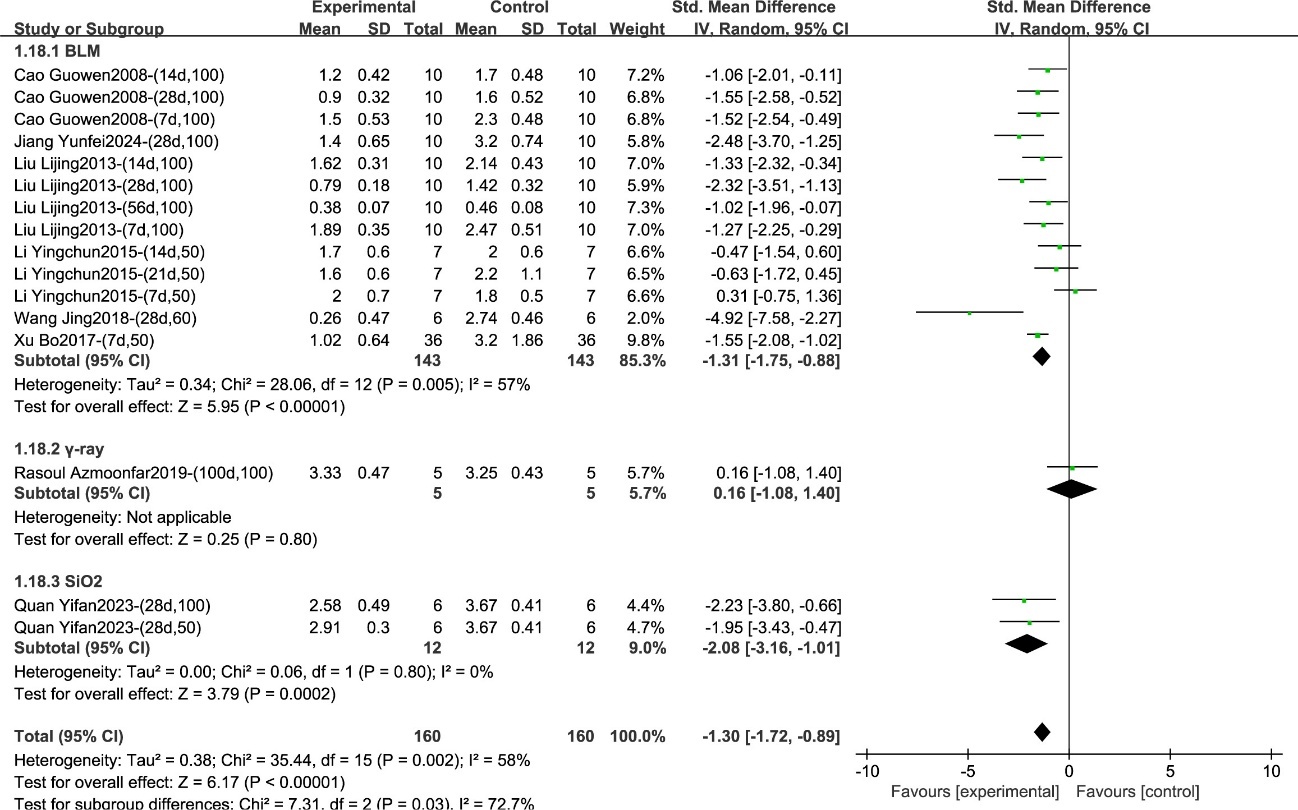


### (D) According to RES administration route


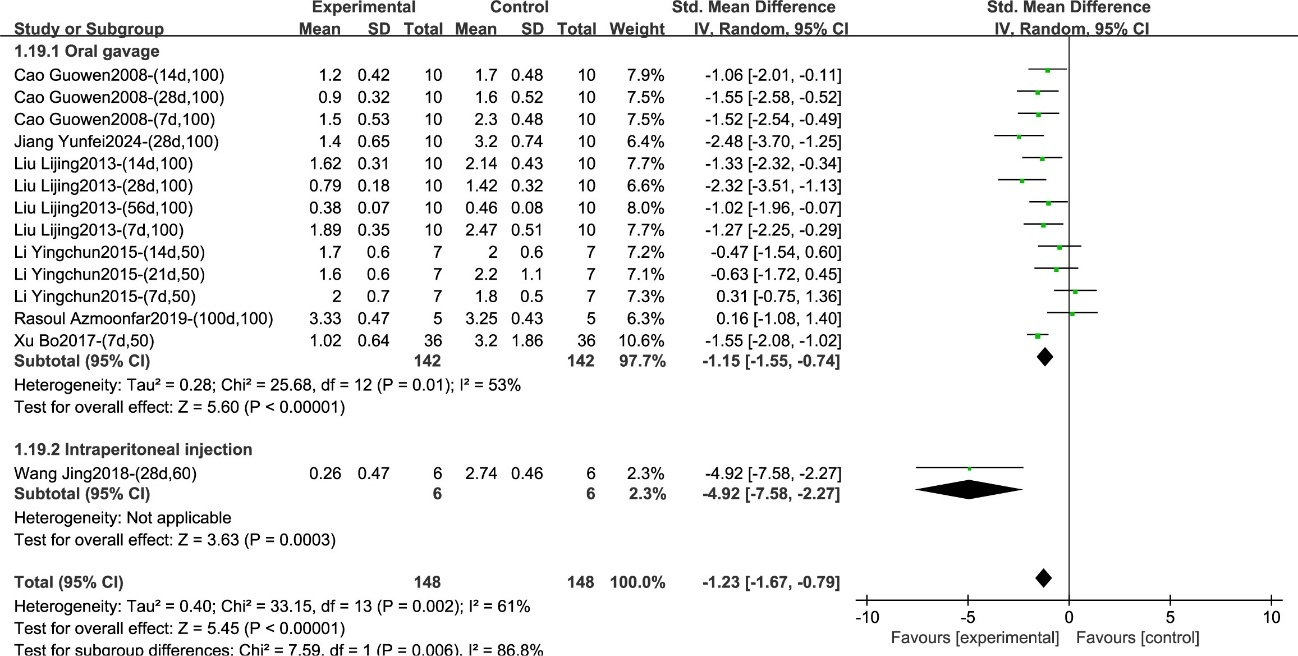


## Supplementary Figure 6 Subgroup analysis of TGF-β content

### (A) According to animal strain


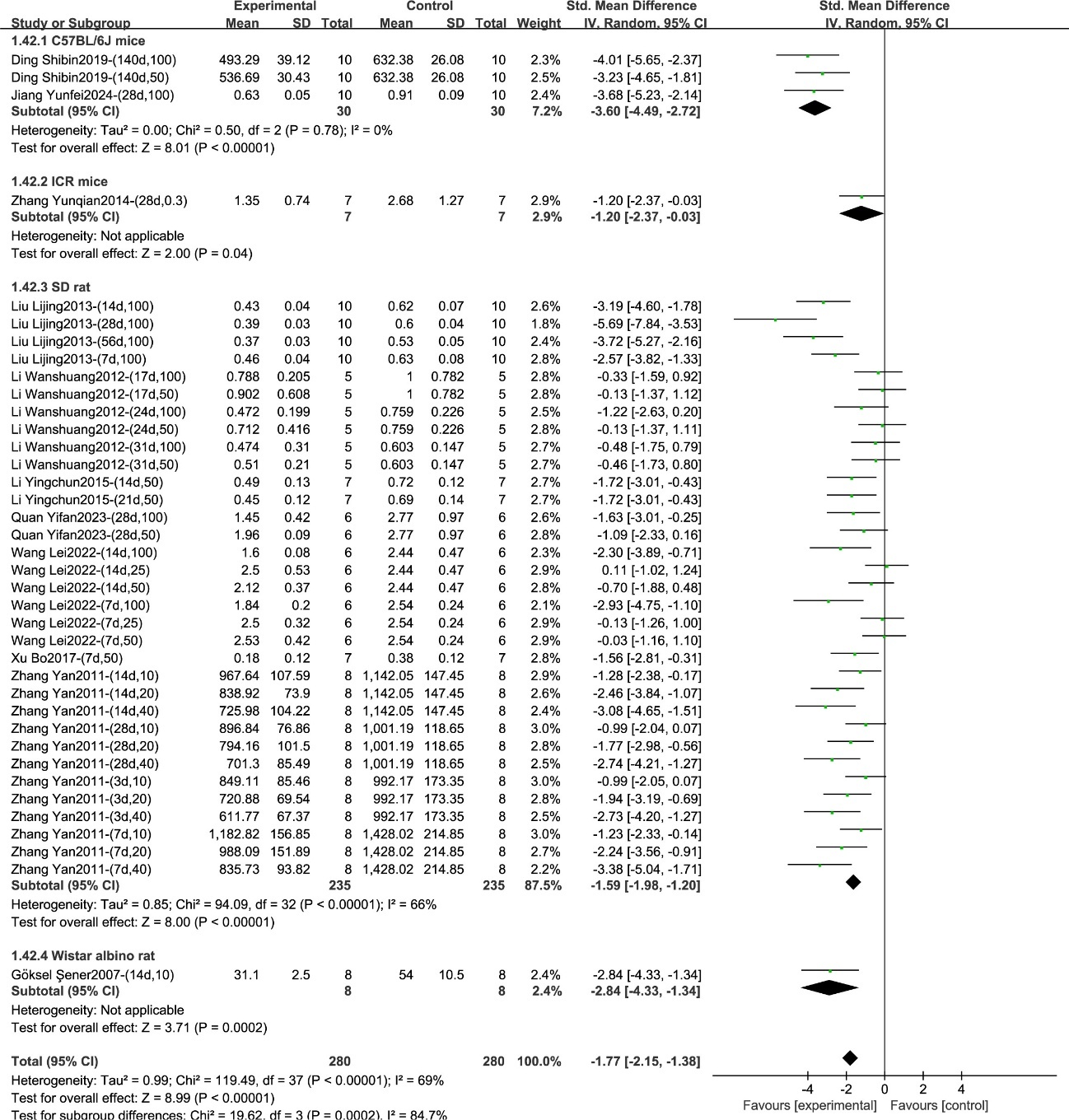


### (B) According to pulmonary fibrosis modeling method


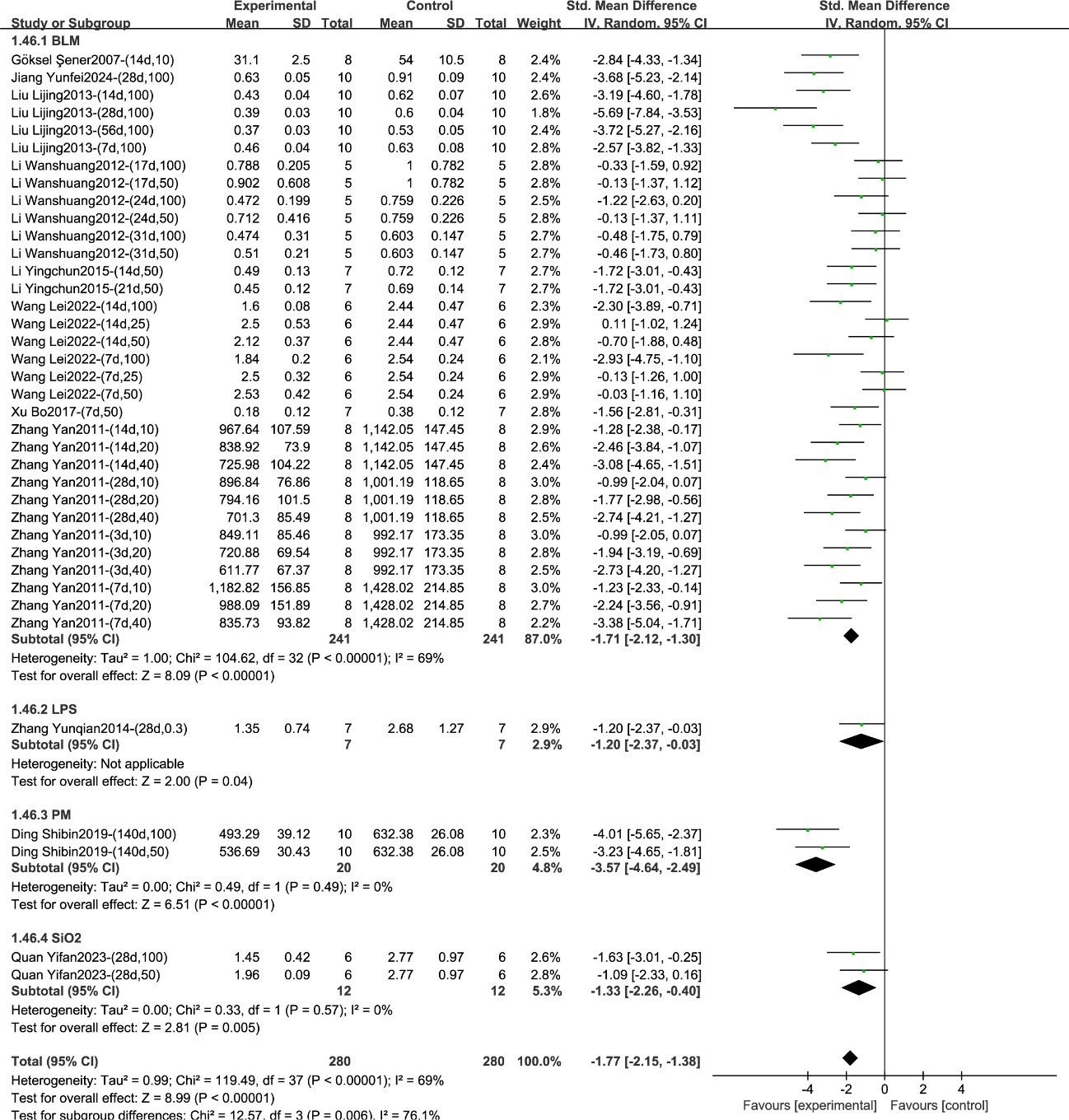


## Supplementary Figure 7 Subgroup analysis of NF-κB content

### (A) According to RES drug source


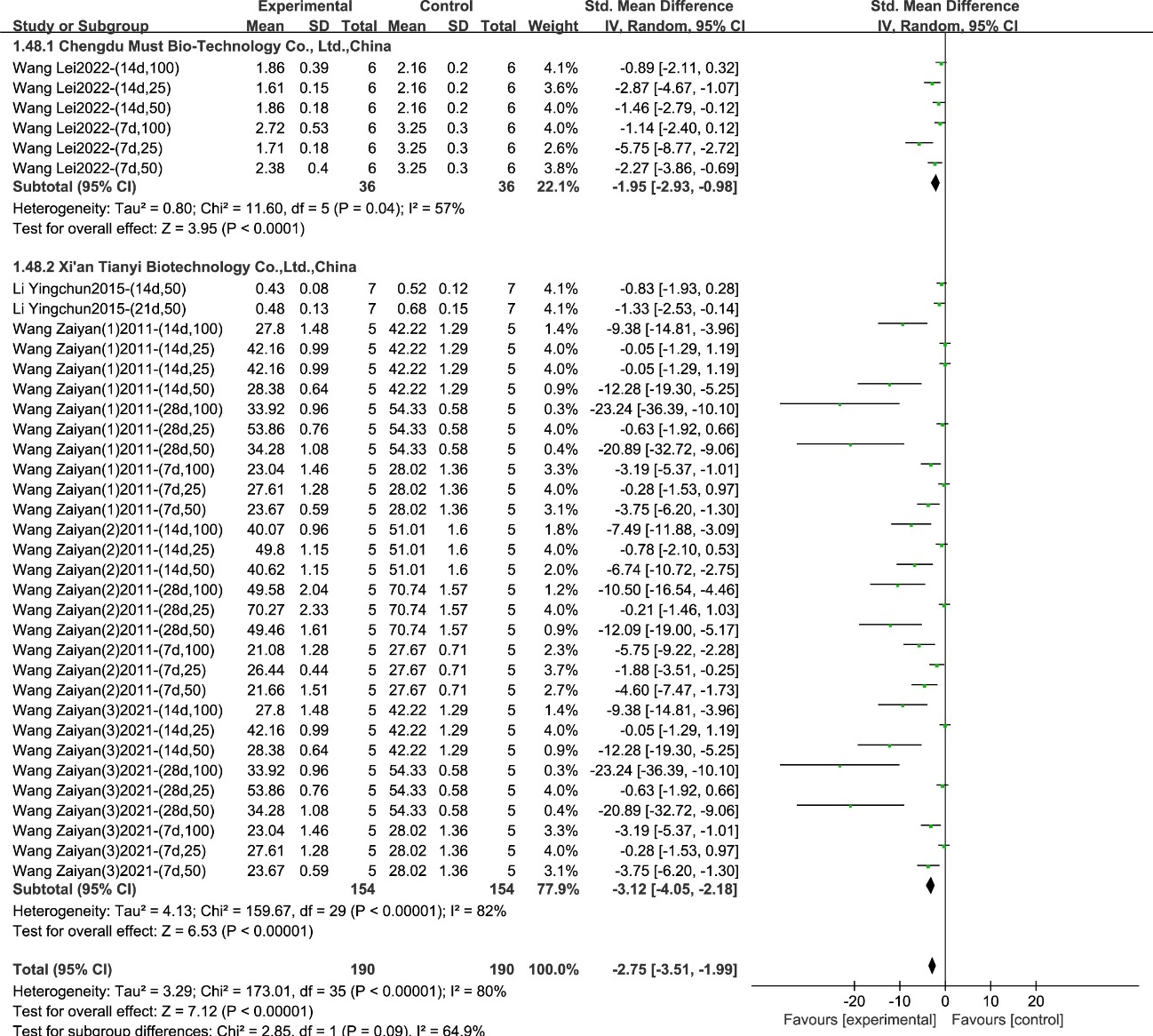


### (B) According to RES dosage


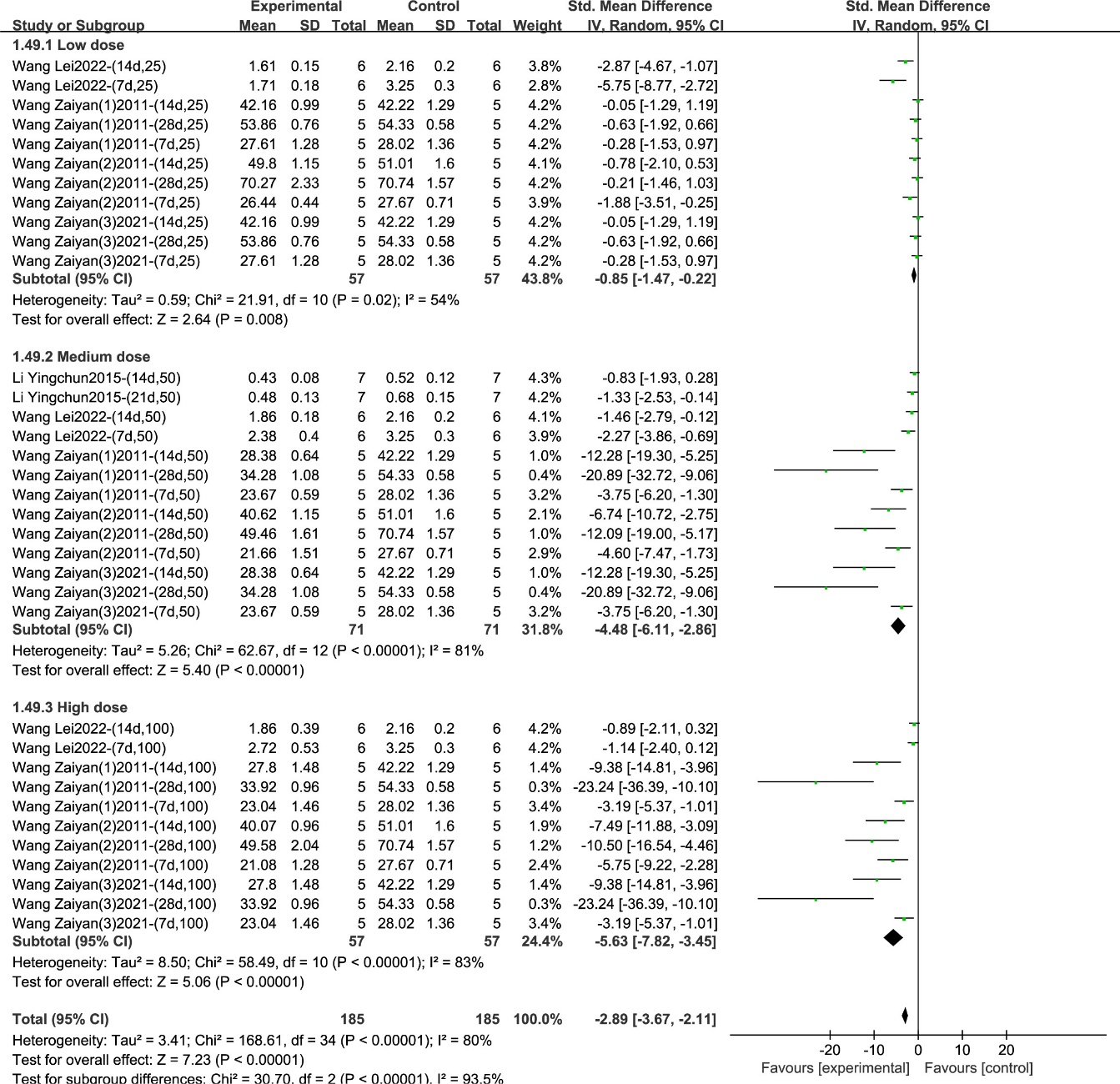


## Supplementary Figure 8 Subgroup analysis of IL-1β content

### (A) According to RES dosage


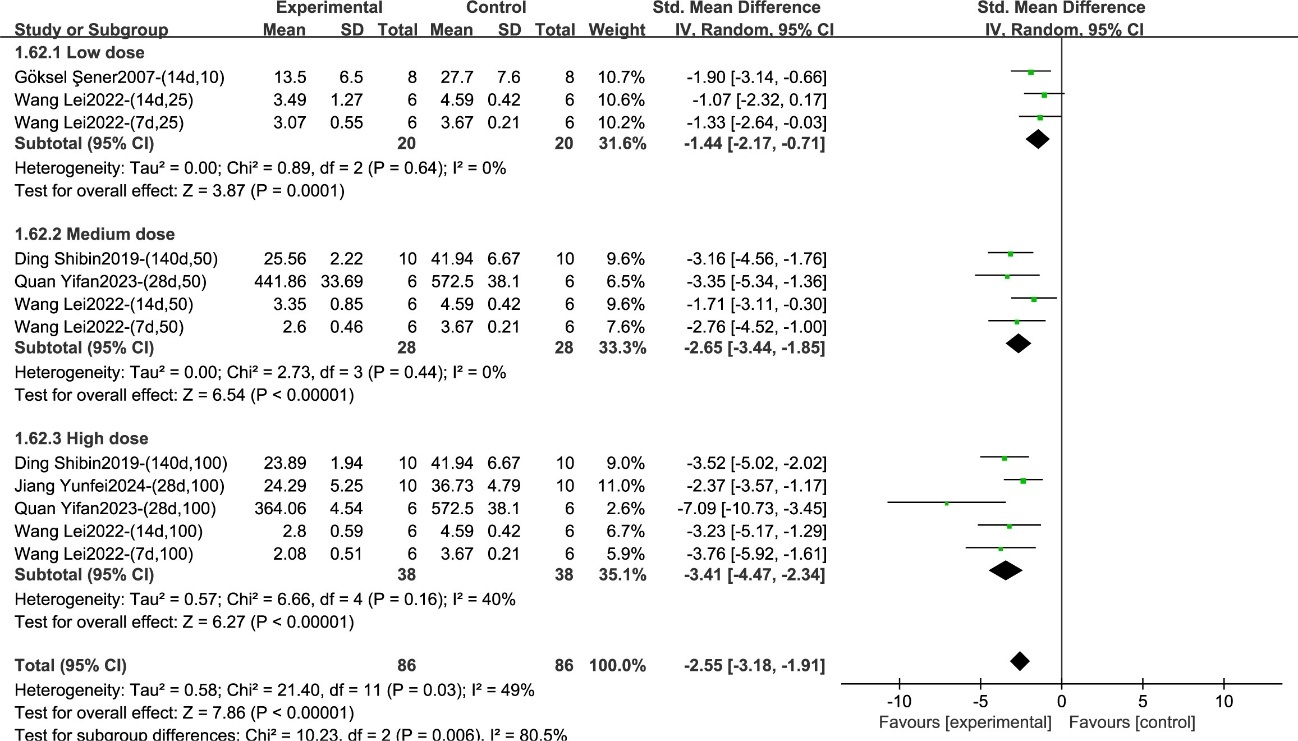


### (B) According to pulmonary fibrosis modeling method


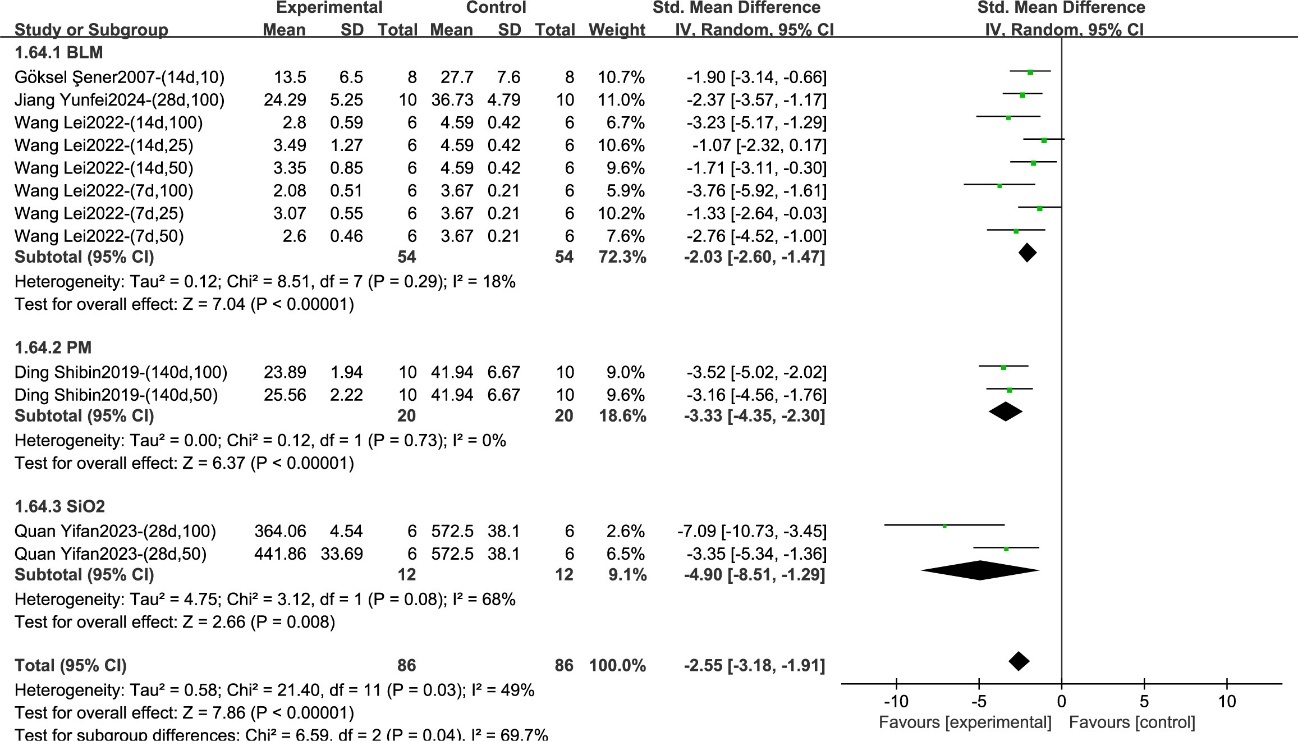


## Supplementary Figure 9 Subgroup analysis of IL-6 content

### (A) According to RES drug source


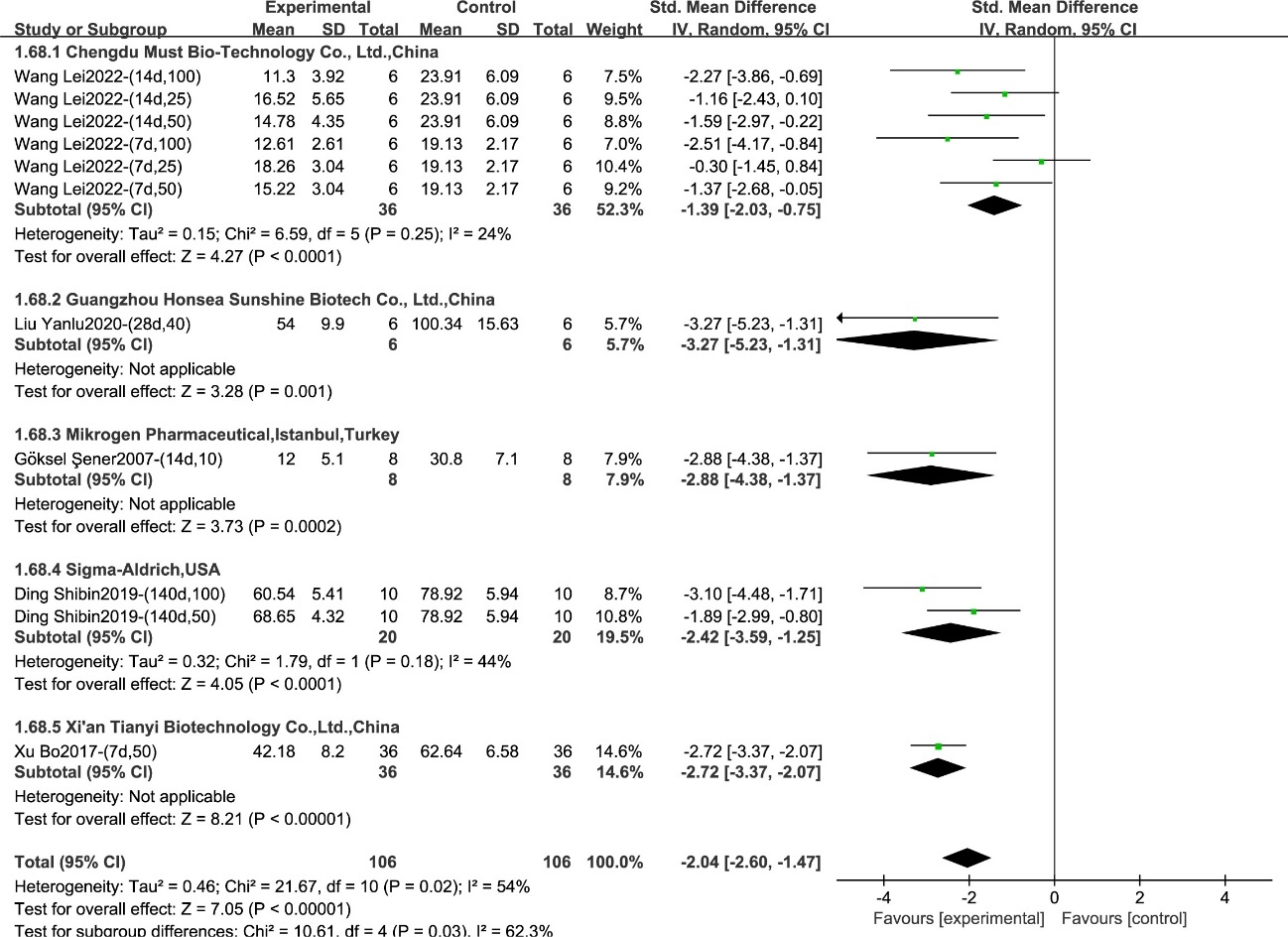


### (B) According to RES administration route


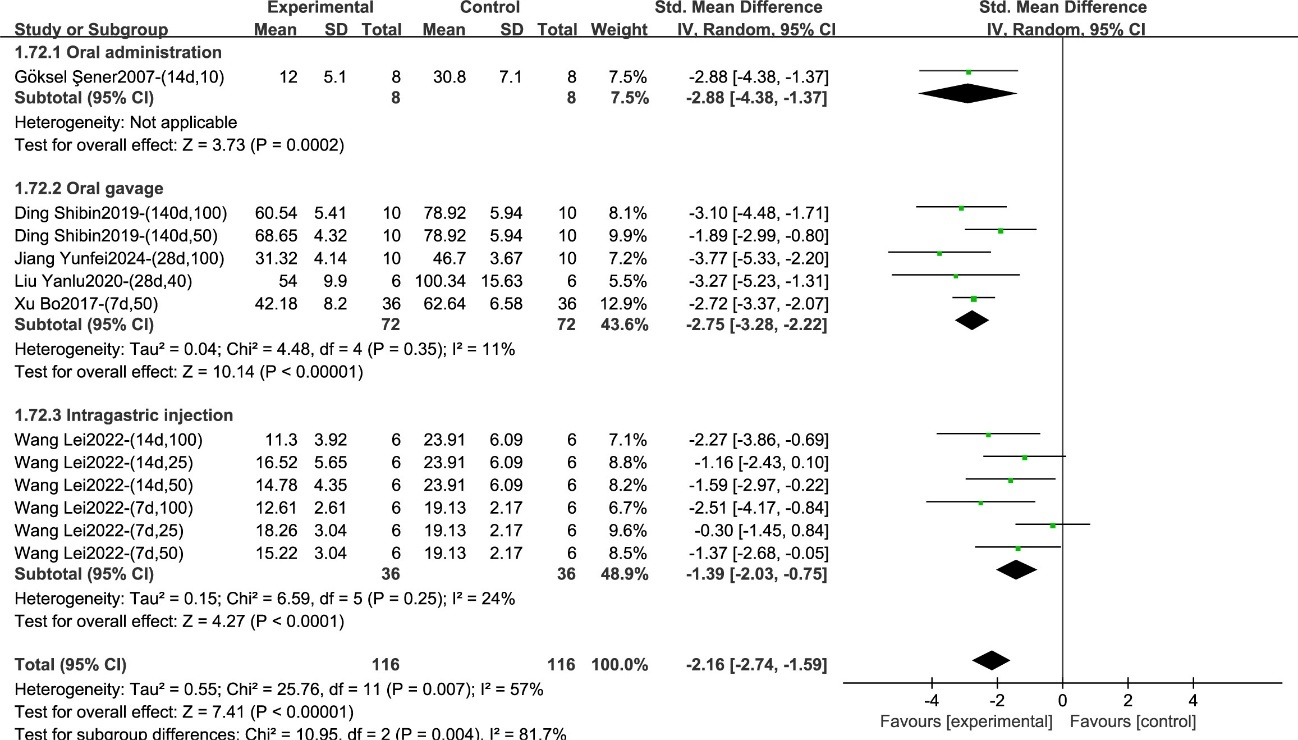


## Supplementary Figure 10 Effect of RES on MDA content in lung tissues of PF animals

### (A) Forest plot of MDA content


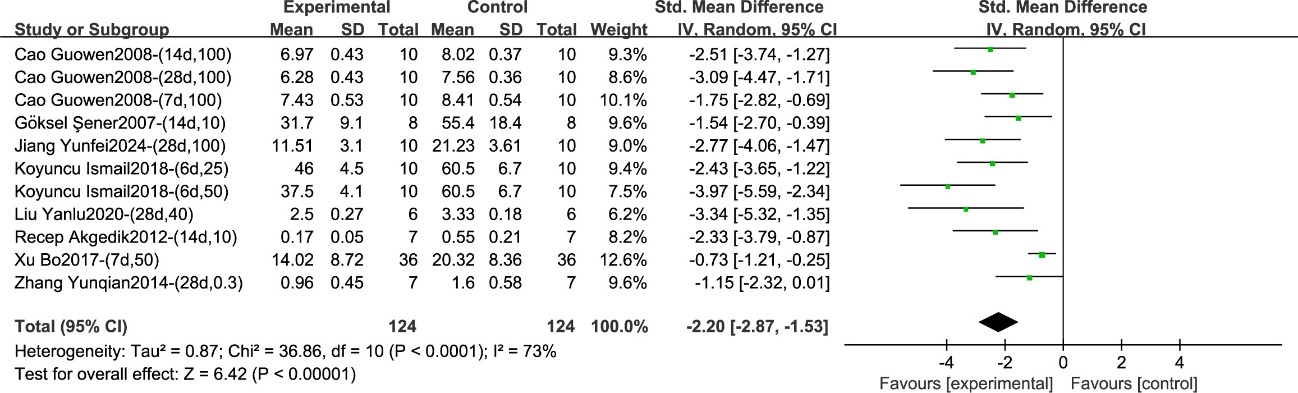


### (B) Sensitivity analysis of MDA content


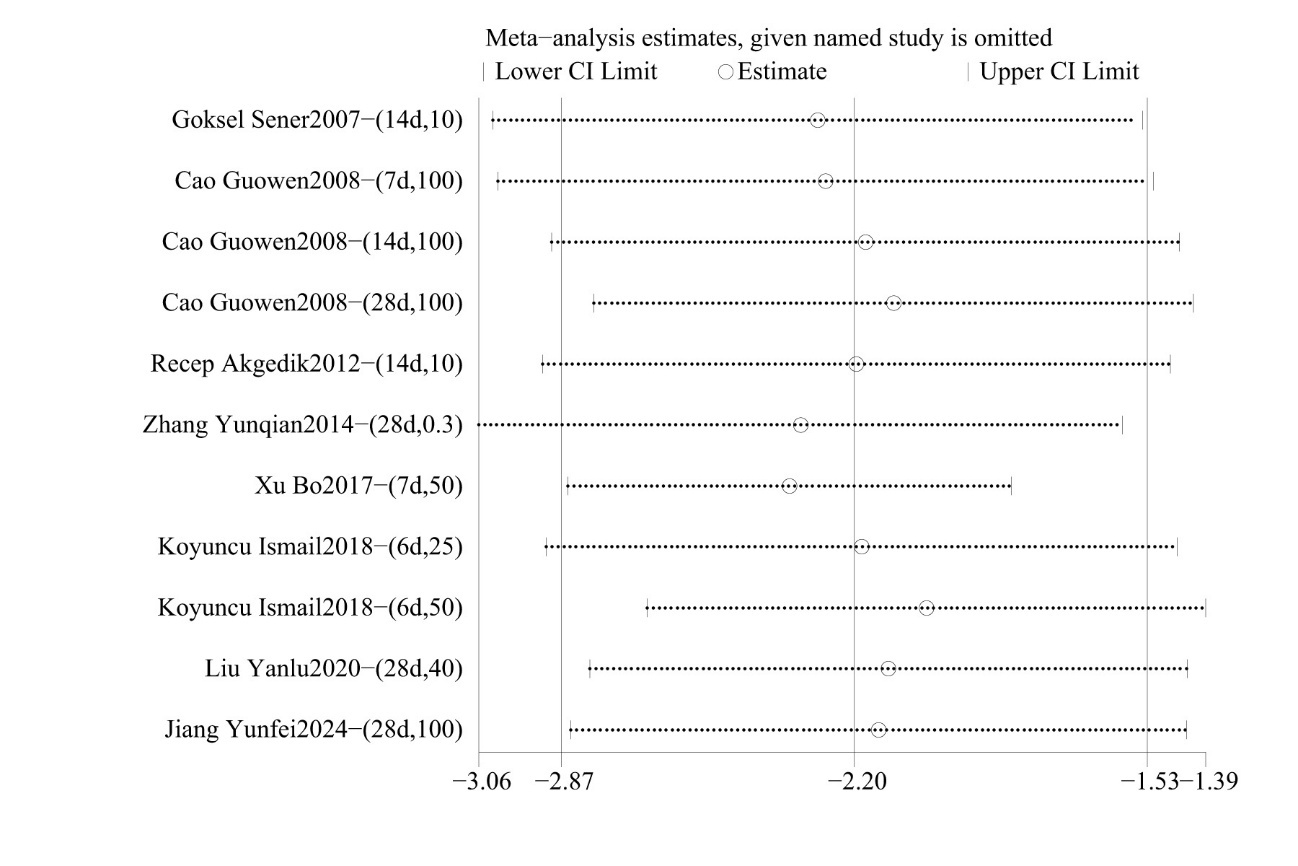


### (C) Funnel plot of MDA content


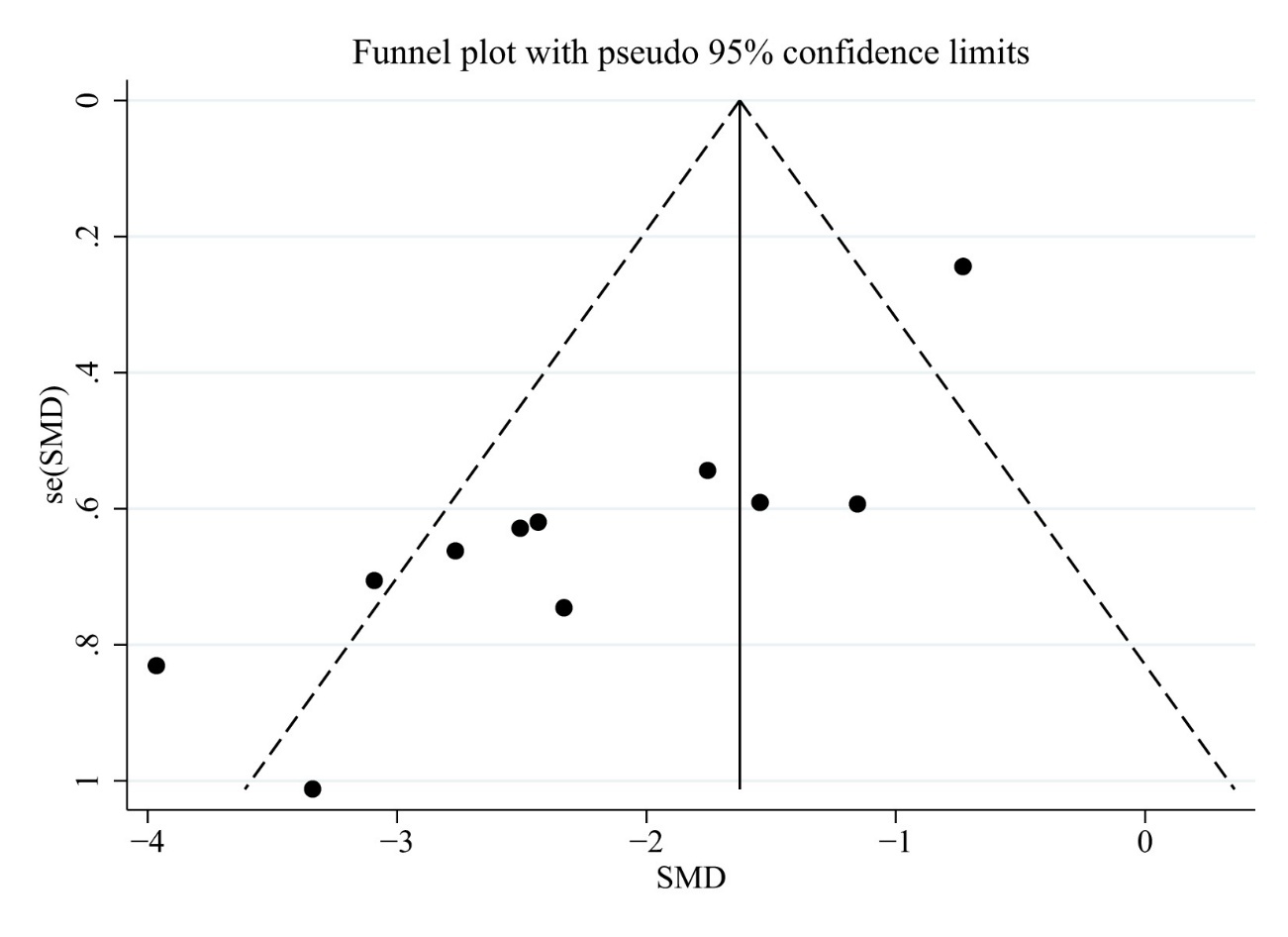


### (D) Egger test for MDA content


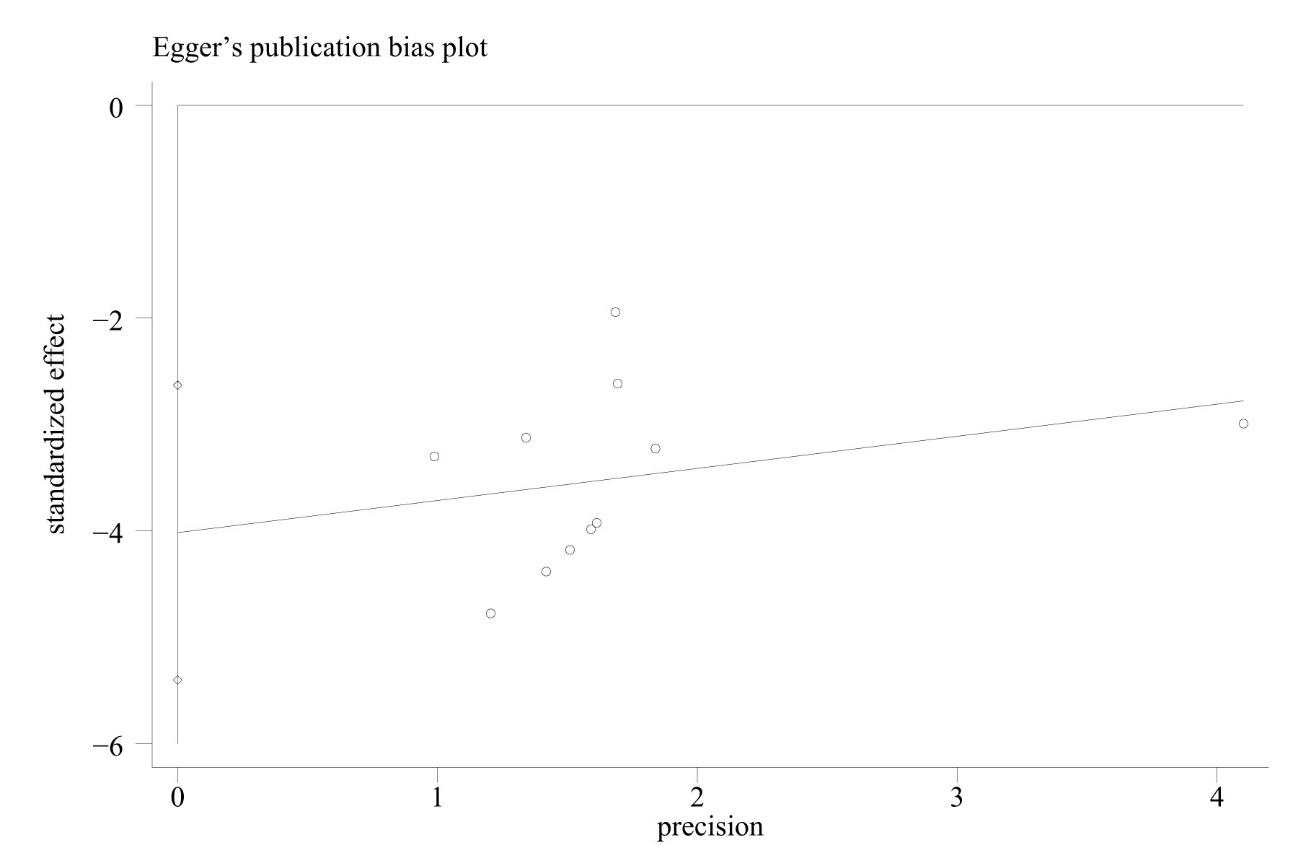


## Supplementary Figure 11 Effect of RES on MPO content in lung tissues of PF animals.

### (A) Forest plot of MPO content


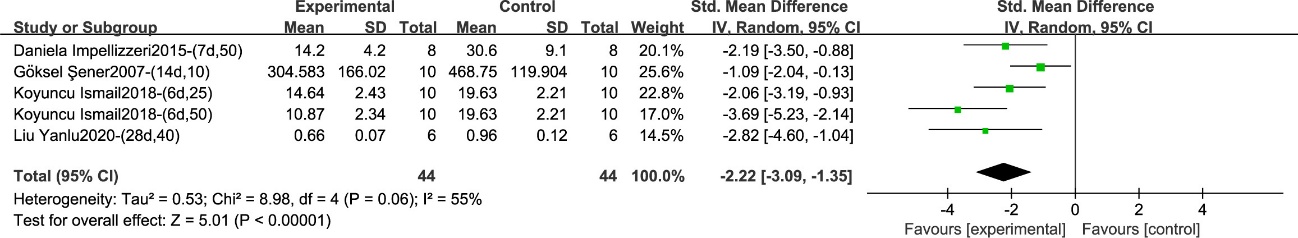


### (B) Sensitivity analysis of MPO content


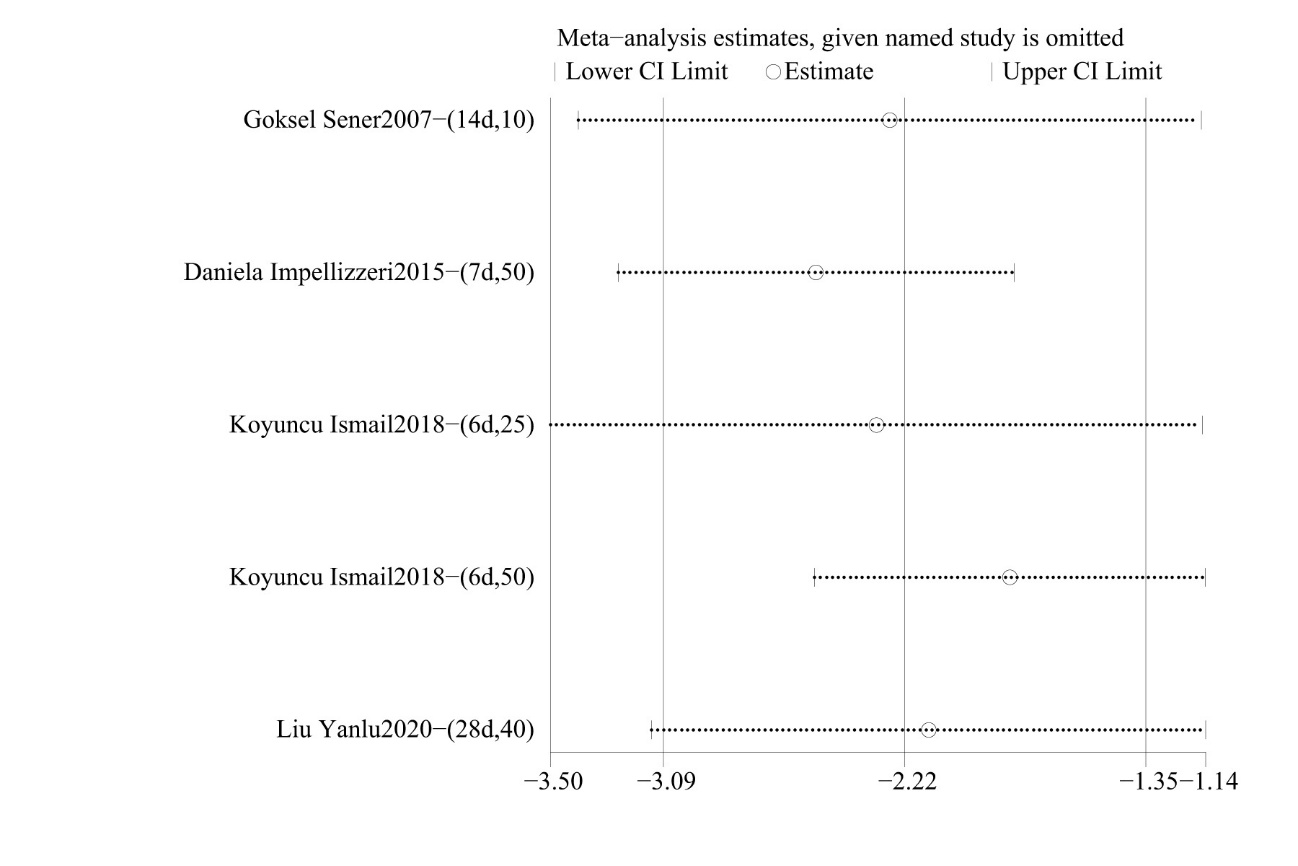


## Supplementary Figure 12 Subgroup analysis of SOD content

### (A) According to RES drug source


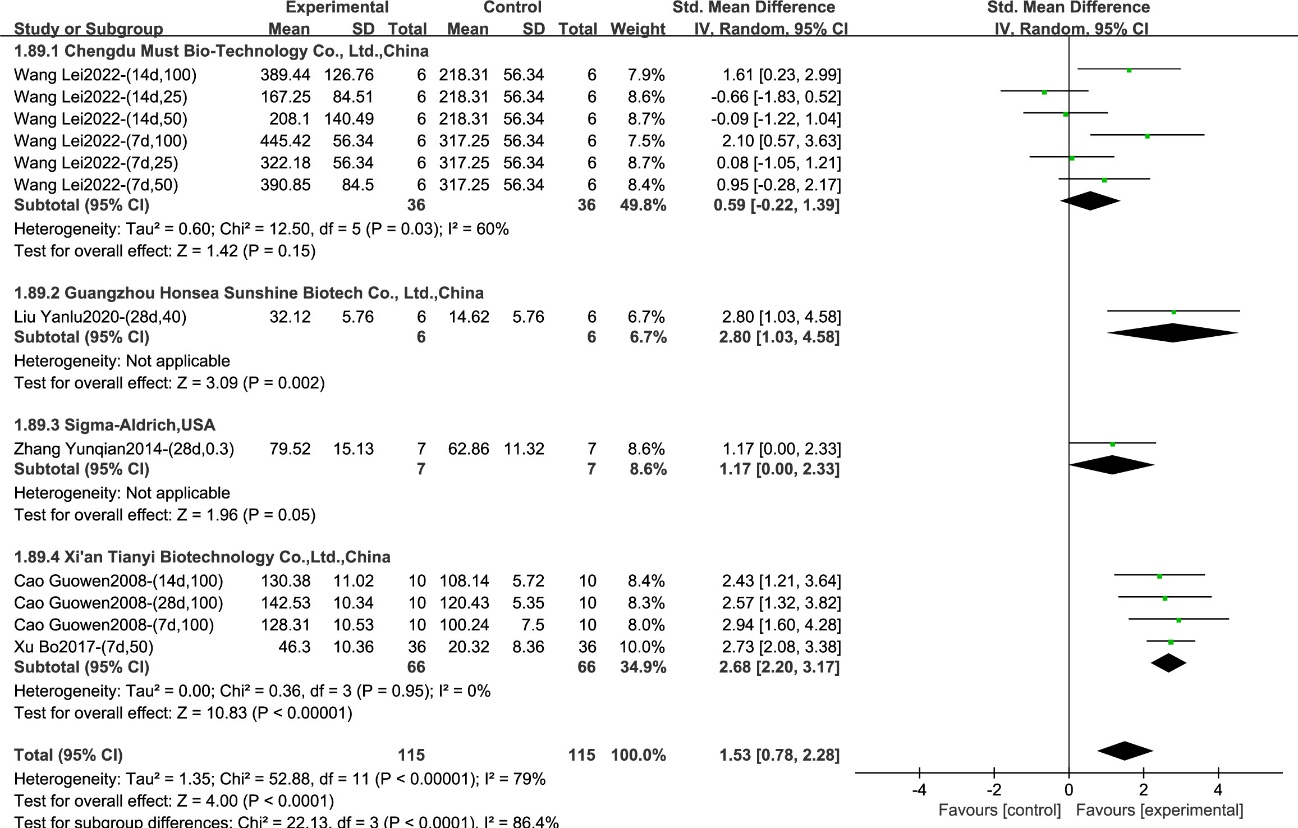


### (B) According to RES dosage


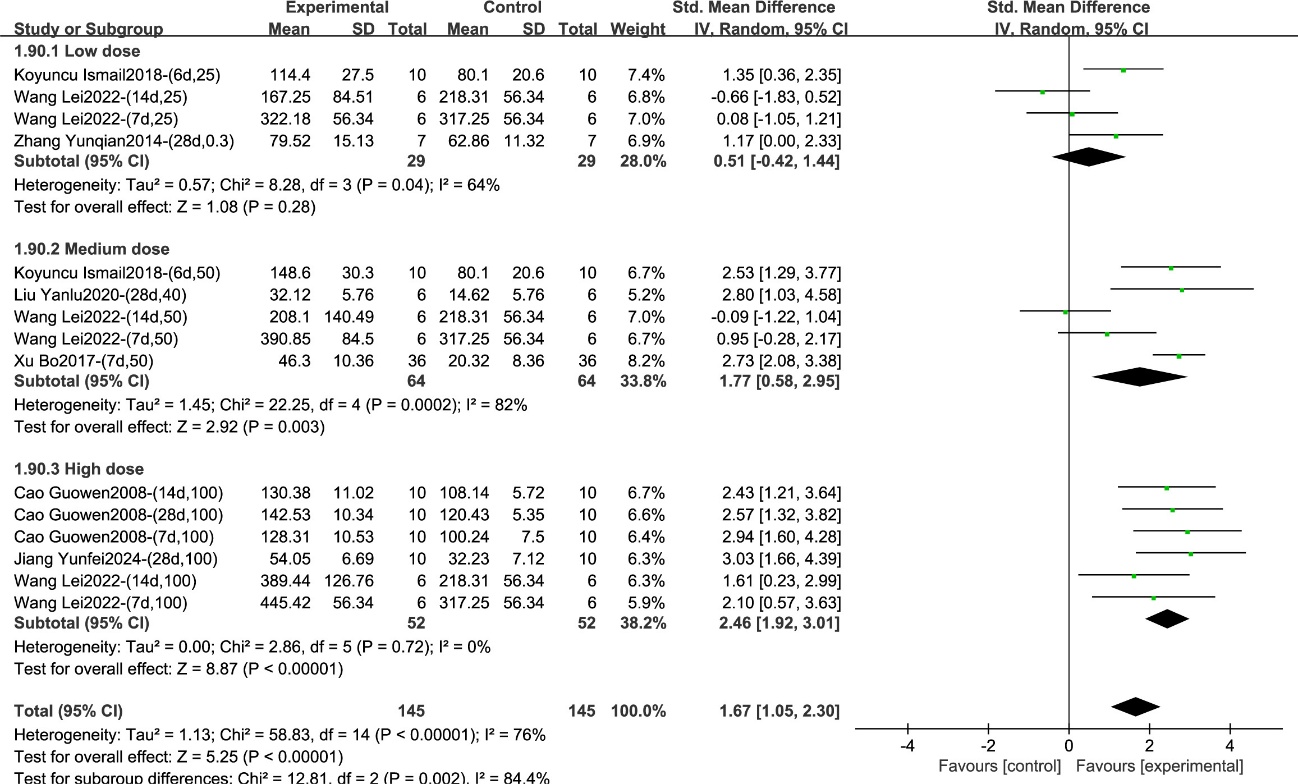


### (C) According to RES administration route


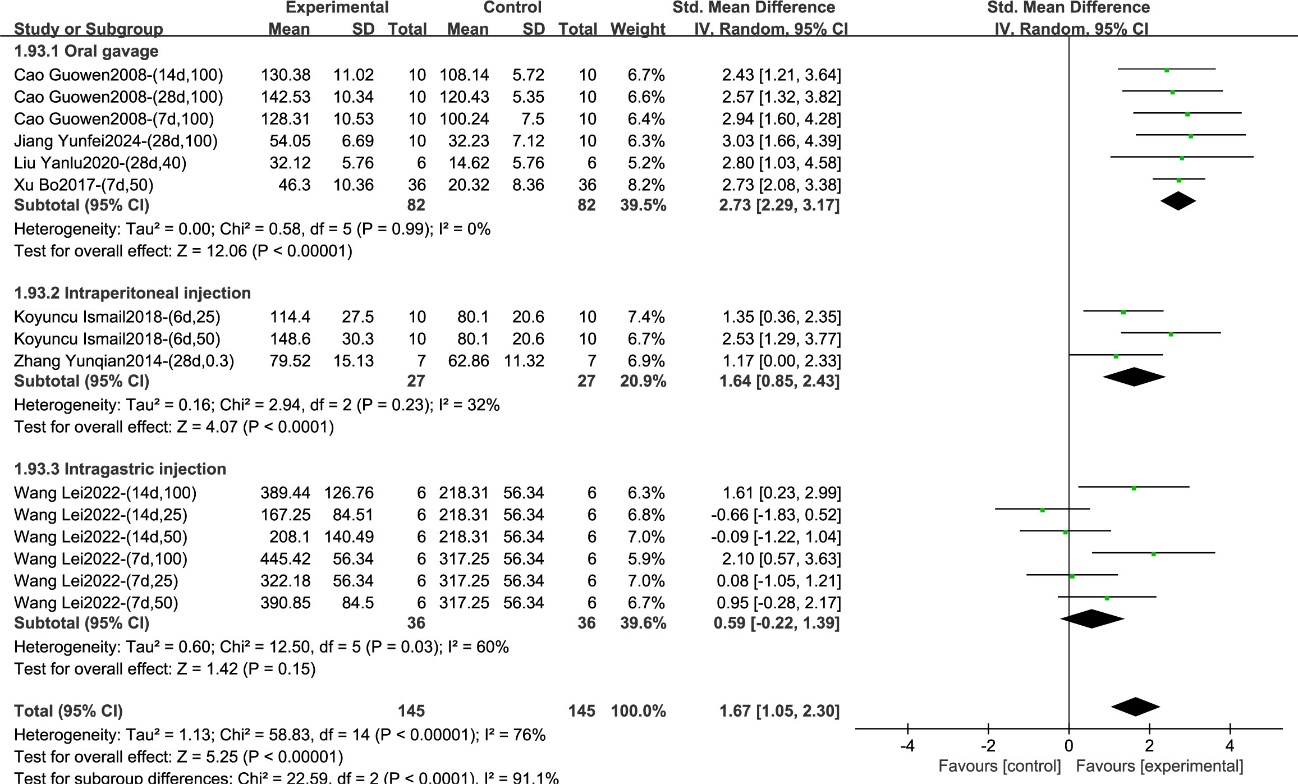

Supplement: Supplementary file 1 [file Supplementaryfile1.docx]
